# Thin and ephemeral snow shapes melt and runoff dynamics in the Peruvian Andes

**DOI:** 10.1038/s43247-025-02379-x

**Published:** 2025-06-05

**Authors:** Catriona L. Fyffe, Emily Potter, Evan Miles, Thomas E. Shaw, Michael McCarthy, Andrew Orr, Edwin Loarte, Katy Medina, Simone Fatichi, Rob Hellström, Michel Baraer, Emilio Mateo, Alejo Cochachin, Matthew Westoby, Francesca Pellicciotti

**Affiliations:** 1 Department of Geography and Environmental Sciences, Northumbria University, Newcastle upon Tyne, UK; 2 Earth Science, Institute of Science and Technology Austria, Klosterneuburg, Austria; 3 Department of Atmospheric and Cryospheric Sciences, University of Innsbruck, Innsbruck, Austria; 4 School of Geography and Planning, University of Sheffield, Sheffield, UK; 5 Swiss Federal Institute for Forest, Snow and Landscape Research WSL, Birmensdorf, Switzerland; 6 University of Zürich, Zürich, Switzerland; 7 Department of Geosciences, University of Fribourg, Fribourg, Switzerland; 8 British Antarctic Survey, Cambridge, UK; 9 Universidad Nacional Santiago Antúnez de Mayolo, Huaraz, Peru; 10 Department of Civil and Environmental Engineering, National University of Singapore, Singapore, Singapore; 11 Bridgewater State University, Bridgewater, MA USA; 12 École de Technologie Supérieure, Université du Québec, Montréal, QC Canada; 13 Pacific Institute, Oakland, CA USA; 14 Autoridad Nacional del Agua, Huaraz, Peru; 15 School of Geography, Earth and Environmental Sciences, University of Plymouth, Plymouth, UK

**Keywords:** Hydrology, Cryospheric science, Climate change

## Abstract

The snow and glaciers of the Peruvian Andes provide vital water supplies in a region facing water scarcity and substantial glacier change. However, there remains a lack of understanding of snow processes and quantification of the contribution of melt to runoff. Here we apply a distributed glacio-hydrological model over the Rio Santa basin to disentangle the role of the cryosphere in the Andean water cycle. Only at the highest elevations (>5000 m a.s.l.) is the snow cover continuous; at lower elevations, the snowpack is thin and ephemeral, with rapid cycles of snowfall and melt. Due to the large catchment area affected by ephemeral snow, its contribution to catchment inputs is substantial (23% and 38% in the wet and dry season, respectively). Ice melt is crucial in the mid-dry season (up to 44% of inputs). Our results improve estimates of water fluxes and call for further process-based modelling across the Andes.

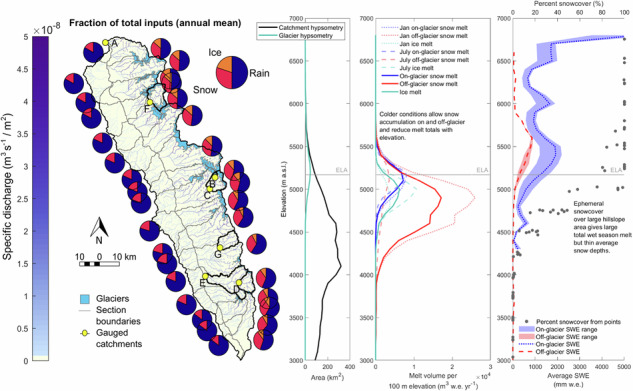

## Abstract (web summary)

Ephemeral snow and ice melt contribute substantially to dry season runoff in the Rio Santa basin, with snowmelt accounting for up to 55% and ice melt up to 44% of inputs, according to results from a distributed glacio-hydrological model applied to quantify cryospheric contributions to the Andean water cycle.

## Introduction

In the tropical Andes of Peru snow and glaciers provide meltwater, influence seasonal and long-term discharge patterns, balance dry season runoff variability, and compensate for reduced runoff from non-glacierised areas during warm and dry conditions^1–4^. This water resource is vital for municipal water use, hydropower, mining, large-scale industrial agriculture, and small-scale highland farming^5,6^. Constructive management of water resources is challenging due to the combination of glacier shrinkage, competing water demands, and unstable water governance, leading to water conflicts in the region^5^.

**Fig. 1:**
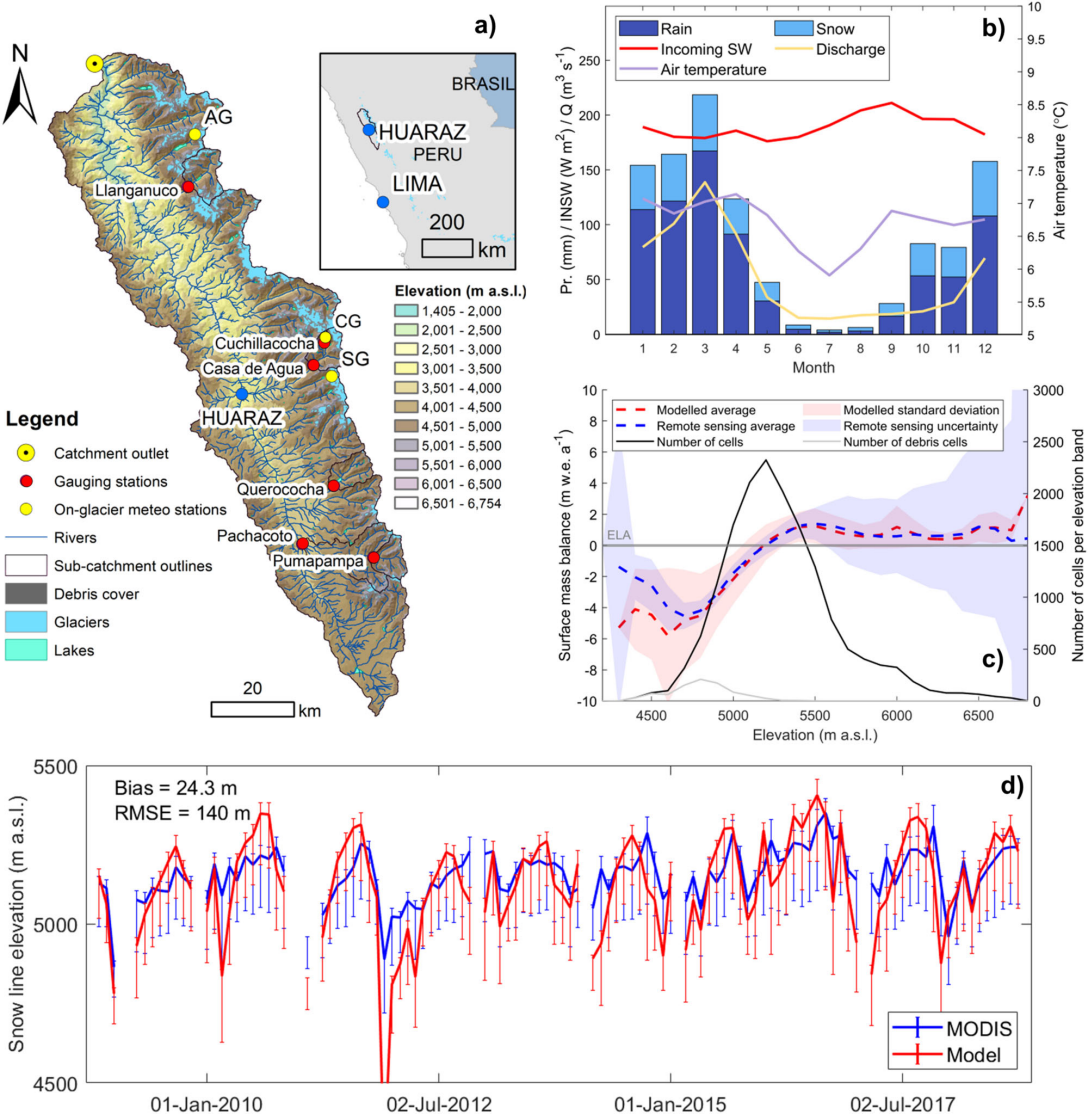
Study area, its climatology, and evaluation of the model outputs. **a** Upper Rio Santa catchment and its topography, including the hydrological (red dots) and meteorological stations (yellow dots) used in this study (AG is Artesonraju Glacier, CG is Cuchillacocha Glacier, and SG is Shallap Glacier). The inset map shows the location of the catchment in Peru. Glacier (blue) and debris cover (grey) areas are from ref. ^66^, lake areas (turquoise) were provided by INAIGEM, and the digital elevation model is an ALOS PALSAR from 2000. **b** Modelled monthly averaged meteorological conditions and discharge (Q, yellow line) for the catchment, including near-surface air temperature (purple line), precipitation (Pr., dark blue bars for rain and light blue bars for snow), and incoming shortwave radiation (INSW, red line). Conditions are an average over the 2008-2018 modelling period. **c** Comparison of modelled (2015-2018, red dashes) and remote sensing (2015-2019, blue dashes) derived altitudinal glacier mass balance profile for the entire catchment. The dashed lines are the average mass balance for all the cells in each 100 m elevation band. The shading for the model results (pink) represents +/− the standard deviation of the mass balance per elevation band, whereas the shading for the remote sensing data (light blue) represents the uncertainty of the average. **d** Comparison of monthly averaged snowline elevations between MODIS (blue lines) and the model (red lines), calculated using an approach following Meier^67^. Modelled cells are defined as snow-covered when the snow water equivalent is > 10 mm w.e., with the error bars representing when this is varied from >1 mm w.e. (minimum) to >20 mm w.e. (maximum). The MODIS error bars represent the snowline derived using alternate Normalised Difference Snow Index threshold values of 0.1 (minimum) and 0.45 (maximum).

In the Cordillera Blanca, the world’s largest tropical glacier mountain range, glacier melt has been assumed to be an important contributor to runoff, especially in the dry season and during droughts^7–9^, while much less clarity exists as to the role, quantities and spatio-temporal dynamics of snow. There is emerging evidence that the snowpack in the Cordillera Blanca is particularly dynamic^10^, likely because of the summer-accumulation regime where air temperatures remain close to melting conditions during the wet season^11^. The region is defined by its strong precipitation, rather than temperature seasonality, with a wet austral summer and dry austral winter, and larger diurnal than seasonal variations in air temperature (Fig. 1b)^12,13^.

On glaciers, wet season precipitation patterns strongly control melt rates, as snow increases the albedo compared to a bare ice surface, reducing melt rates and protecting the ice beneath. The relationship between precipitation and glacier melt is especially strong because the wet season snowpack over glacier ablation zones is ephemeral, forming and melting over periods of days to weeks^10,14^. This is in contrast to the continuous winter snowpacks found on European glaciers. However, such insights are limited to glaciological studies, and we currently lack understanding of the importance and dynamics of off-glacier snow, its hydrological significance, and future changes.

The fragile Andean cryosphere is threatened by a warming climate. The Cordillera Blanca has already faced substantial glacier loss, with 46% of glacier area lost between 1930 and 2016^15^ and mass losses of −205+/−107 kg m^-2^ a^-1^ between 2000 and 2016^16^ (for subregion R1, which includes the Cordillera Blanca). Future climate change will considerably influence the region, with air temperature and precipitation projected to increase within the Rio Santa catchment, Peru, by around +3.6 °C and +12%, respectively, by the late 21^st^ century under the high-emission Representative Concentration Pathway 8.5^17^. This would likely result in a large proportion of the snow cover and glaciers disappearing^11^. Importantly, an increase in climatic extremes, notably meteorological droughts and extreme precipitation events, is predicted to impact the region by the late 21^st^ century^17^.

The hydrologic importance and climatic sensitivity of the region’s snow and ice cover have led to a number of important studies in the Cordillera Blanca. These, however, have not been able to quantify the snowfall dynamics at the catchment scale, nor differentiate the contributions of snow (both on and off-glacier) and ice melt to runoff. Water balance modelling has assumed that the change in catchment storage equals the glacier melt contribution, with changes in on-glacier snow storage implicitly included within the glacier contribution, and off-glacier snow neglected^7,8,18,19^. Hydrochemistry analysis has quantified the overall contribution of meltwater to runoff at a given point in time, but has been unable to separate the glacier ice and snow components^7,8,20,21^.

Previous catchment modelling in the Peruvian Andes has also made simplifying assumptions in the calculation of glacier mass balance due to lack of data or model limitations^9,22,23^, with some approaches neglecting treatment of off-glacier snow entirely^9,18,22^. Most models rely only on discharge for calibration and validation, with no evaluation of model skill against independent observations of snow or glacier processes^9,24–26^. Model calibration against discharge alone introduces a high risk of equifinality, where similar modelled discharge can result from different parameter sets^25^. Exceptions include Condom et al.^27^, who compared glacier area evolution for the Rio Santa catchment, and Aubry-Wake et al.^28^, who compared modelled glacier mass balances against published values in the Quilcayhuanca Basin, a sub-catchment of the Rio Santa. Condom et al.^27^’s application of the Water Evaluation and Planning model to the Rio Santa catchment successfully allowed the testing of climate change scenarios on flows available for hydropower, although, since this model is semi-distributed and operates on a monthly timestep, it is less useful for understanding finer resolution processes. Aubry-Wake et al.^28^’s simulations with the Cold Regions Hydrological Modelling Platform explored the importance of the groundwater contribution to streamflow, which was higher in the dry (37%) compared to the wet (10%) seasons. They also investigated future runoff scenarios, finding that the future glacier cover substantially influenced the catchment response to climate.

Here, we provide a spatially and temporally resolved understanding of cryospheric processes and their hydrological role in the Peruvian Andes. We apply the physically oriented glacio-hydrological model TOPKAPI-ETH (e.g., refs. ^29–33^) to the upper Rio Santa catchment, Cordillera Blanca, Peru (Fig. 1a). The Cordillera Blanca is the world’s largest tropical glacier mountain range^5^ and serves as an ideal case study given the abundance of ground observations. The similarity of its hypsometry to other high-elevation basins across the Peruvian Andes (see Discussion) also allows us to draw wider conclusions relevant to Peru. The model is fully distributed and applied for a ten-year period (1st November 2008 to 31st October 2018) at an hourly timestep, with model forcing provided by Weather Research and Forecasting (WRF) atmospheric model simulations (see Methods). We calibrate model parameters against in-situ and remotely sensed data and evaluate model skill using independent discharge records and remotely sensed datasets of snow cover and glacier mass balance. We find that seasonal snowpacks exist only at the highest elevations, with snowpacks below ~5000 m a.s.l. thin and short-lived, although they contribute substantially to inputs into the catchment. We also determined that snow melt is important all year and especially at the beginning of the dry season, whereas ice melt is crucial in the mid-dry season. Using our model results as a basis, we then explore the potential importance of snow melt across the entire Peruvian Andes.

## Results

### Lower elevation snow cover is thin and ephemeral with seasonal snowpacks confined to high elevations

**Fig. 2:**
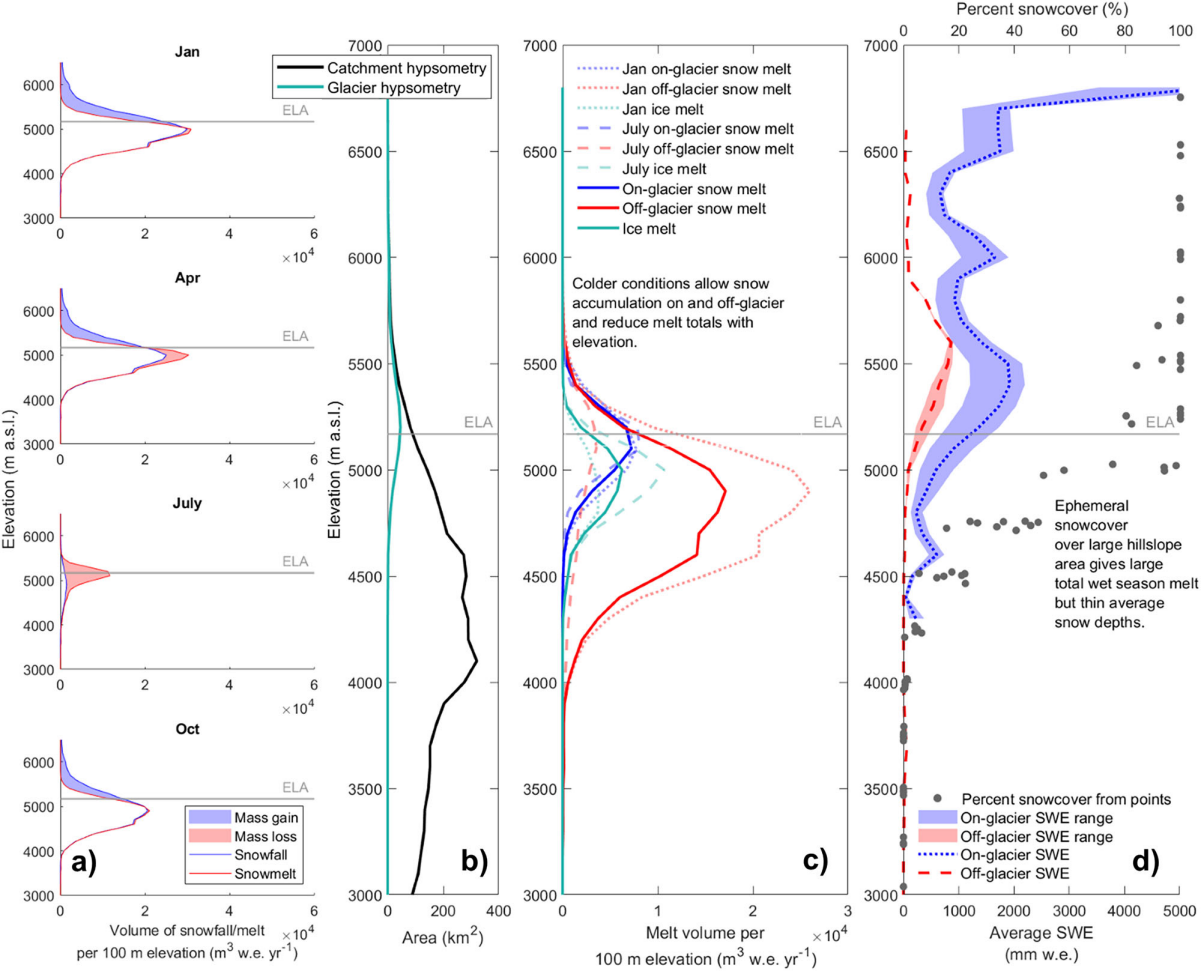
Snowfall and snowmelt dynamics in the upper Rio Santa catchment. **a** Comparison of snowfall (blue lines) and snowmelt (red lines) per elevation band for selected months, with the equilibrium-line altitude (ELA, grey line), the median modelled value across all glaciers. Shaded areas represent mass gain (blue) and mass loss (red). **b** Catchment (black line) and glacier (green line) hypsometry. **c** Total melt volume per elevation band for on- (blue) and off- (red) glacier snow melt and ice melt (green), the solid lines are the average over all months, with small dashes representing mean total melt in January and large dashes representing total melt in July. **d** Average per cell on (blue small dashes) and off (red big dashes) glacier snow water equivalent (SWE), with the shading representing the seasonal range (blue for on-glacier and red off-glacier), and on the top *x*-axis the percentage of time with snow cover at a range of modelled points across the catchment (grey dots). All values are based on model outputs averaged over the 2008−2018 analysis period.

Our modelling demonstrates that seasonal snowpacks exist only at high elevations (above ~5000 m a.s.l.) in the upper Rio Santa catchment (Fig. 2). At lower elevations, snow is ephemeral and confined mainly to the wet season (October to March). At elevations between ~4000 m a.s.l. and ~5000 m a.s.l. wet season snow remains thin and the snow cover is short-lived, lasting hours to days (Fig. 2d). To illustrate this we show an example period between the 21st and 28th of January 2018 (SI Fig. 15). Within this period, snow persists less than a day at 4511 m a.s.l. and for several days at 4758 m a.s.l.. Median snow water equivalent of snow-covered periods from selected points between 4000 and 5000 m a.s.l. is only 2.6 mm w.e. in January, with a median snow cover duration of 16 hours (SI Fig. 16). The annual percentage of time with snow cover is usually <50% at elevations <5000 m a.s.l. (Fig. 2d). These rapid variations in snow cover lead to quick changes in the wet season snow-line elevation (SI Fig. 17). Importantly, the continual snowfall and melt cycles, when combined with the large catchment area between 4000 and 5000 m a.s.l., results in a substantial contribution of snowmelt to catchment inputs (Fig. 2c). In total, off-glacier snow melt contributes 1.26 × 10^5^ m^3^ w.e. a^-1^, compared to 0.30 × 10^5^ m^3^ w.e. a^-1^ for on-glacier snow melt and 0.28 × 10^5^ m^3^ w.e. a^-1^ for ice melt. In the wet season, most of the snowfall and snow melt occurs outside the glaciers due to their larger area compared to on-glacier.

Above ~5000 m a.s.l. snow accumulates in the wet season, forming an increasingly thick snowpack both on and off-glacier (Fig. 2). With the onset of the dry season (from April to September) this high-elevation snow reservoir is depleted and the snowline increases in elevation (SI Fig. 17). During this period, the snow gradually becomes confined to on-glacier areas, so that in the dry season it is the snow on glacier that provides the bulk of melt volumes from elevations 5000–5300 m a.s.l.. This high-elevation snowpack then contributes to catchment inputs in the dry season (shown as mass loss in Fig. 2a). The snow dynamics in the catchment are therefore characterised by (i) a highly ephemeral, thin snowpack below 5000 m a.s.l., which contributes melt in the wet season from both on- and off-glacier areas, (ii) and a higher elevation snowpack, mostly confined to glaciers, which provides a contribution to catchment inputs in the dry season.

### Snow melt is important all year but ice melt is crucial in the dry season

**Fig. 3:**
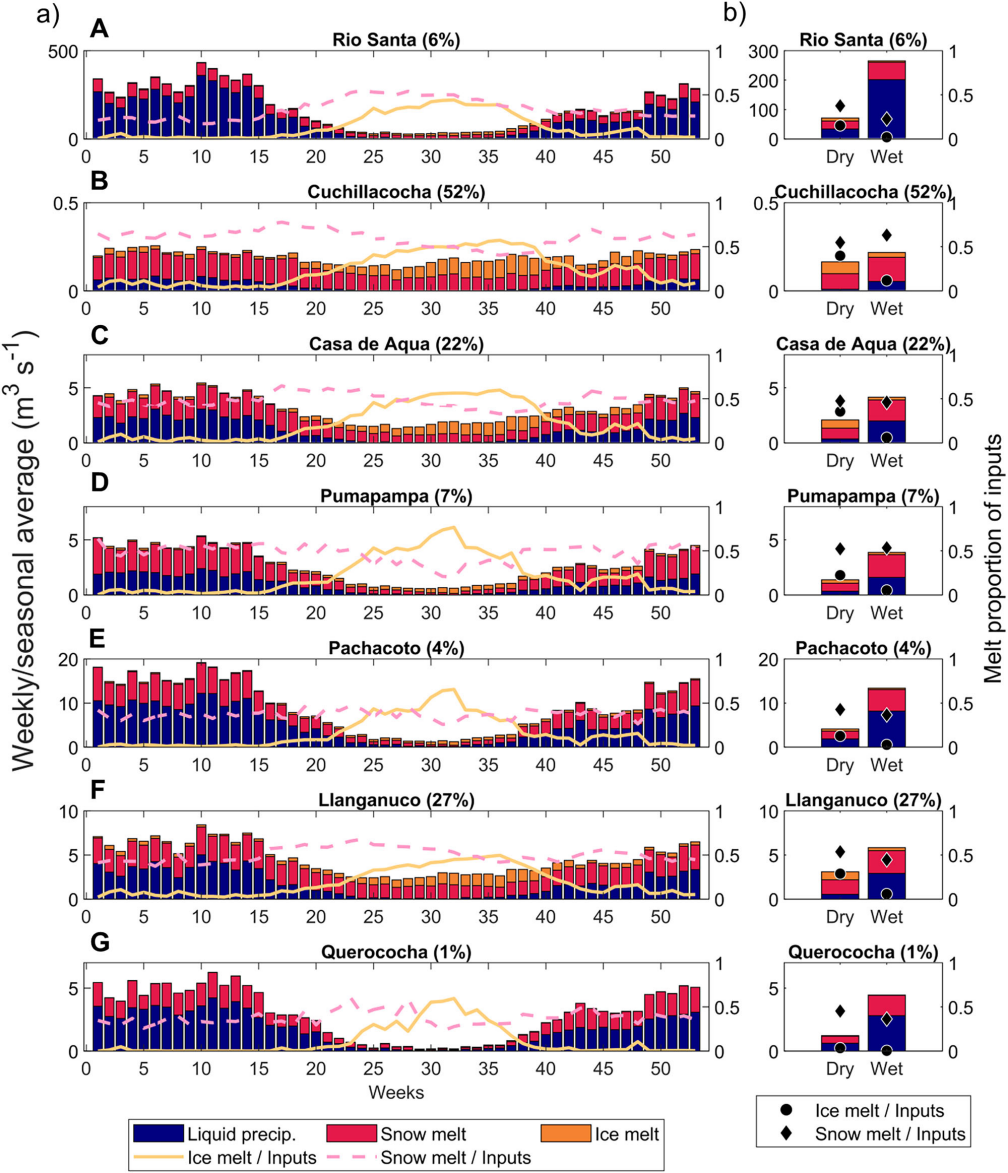
Seasonal contribution of melt to catchment inputs in the upper Rio Santa catchment. **a** Weekly averaged rain (blue bars), snow (cerise bars), and ice melt inputs (orange bars) for the upper Rio Santa and within the six gauged catchments. The secondary *y*-axis values show the fractional contributions of snow (pink dashes) and ice melt (orange line) of the total inputs (sum of liquid rain, snow, and ice melt). **b** As for (**a**), but seasonally averaged and with dots to represent the ice melt proportion of inputs, and diamonds to represent the snowmelt proportion of inputs. Seasonal values are calculated based on the mean of each dry/wet season over all years. The catchment glacier cover is shown in brackets in the title headings. The letters on each panel correspond to the catchment labels in Fig. 4. All values are based on model outputs averaged over the 2008-2018 analysis period.

**Fig. 4:**
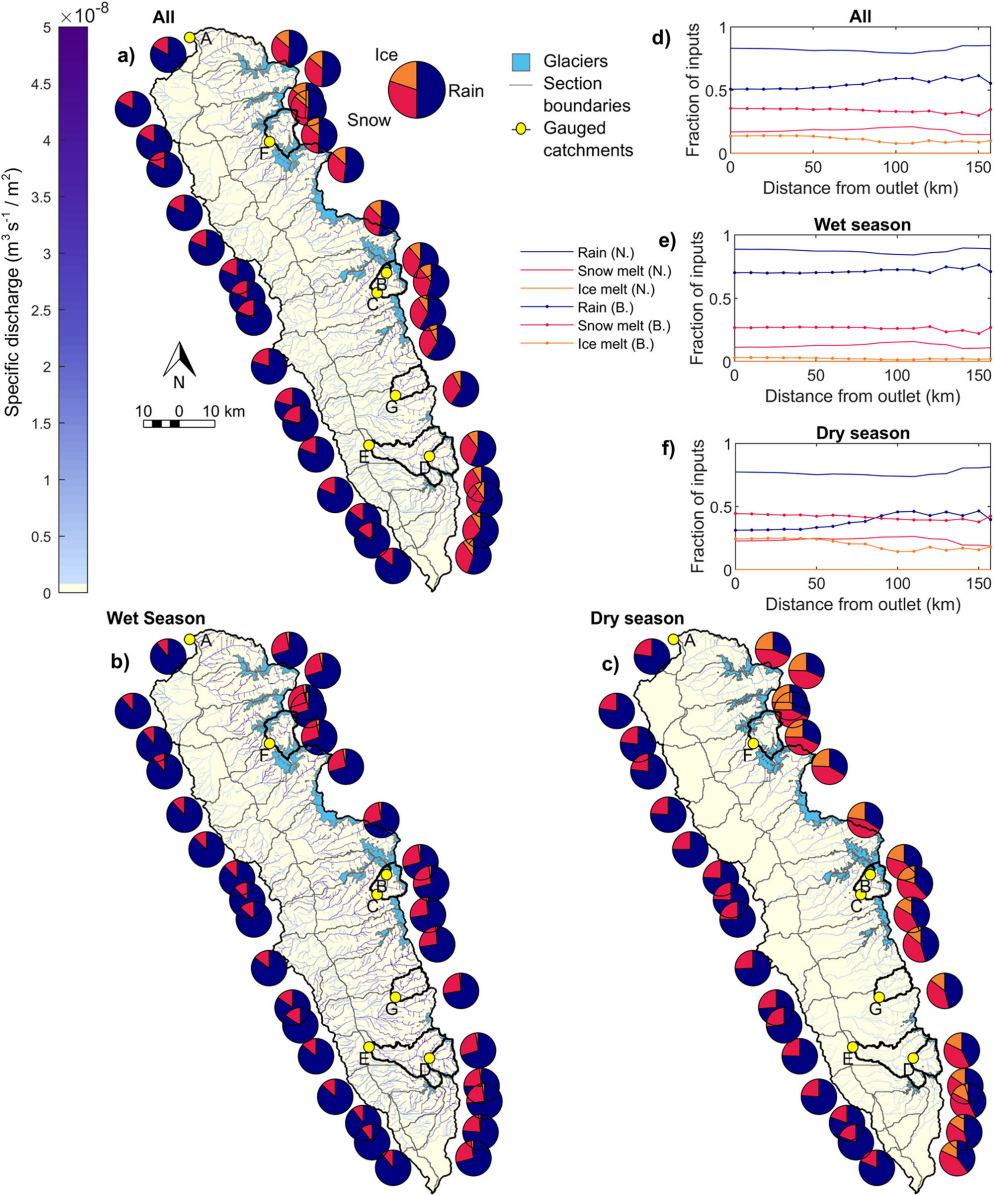
Spatial contribution of melt to catchment inputs. **a** to **c** The stream network (blue river network) shaded to represent specific discharge (scale limited to 0.001 m^3^ s^−1^/m^2^). The pie charts show the proportional contribution of rain (blue), snow melt (cerise) and ice melt (orange) to liquid water inputs (before accounting for evapotranspiration or infiltration) within the section of the catchment they are next to (outlined in grey) and all sections upstream on the same side (the catchment was split into 10 km sections and then by east and west sides). Glacier areas (light blue) are from ref. ^66^, with the gauged catchments shown in black, with the outlet points yellow dots. The letters correspond to the panels in Fig. 3. **d** to **f** The fractional contribution of the inputs in relation to distance to the outlet, with blue lines for rain, cerise lines for snow melt and orange lines for ice melt, lines with no markers represent the Negra (west) side of the catchment, and lines with dot markers represent the Blanca (east) side. The seasonal contributions are calculated here as the mean of the month values in each season. All values are based on model outputs averaged over the 2008–2018 analysis period.

Snow melt forms a substantial contribution to the water inputs into the upper Rio Santa, composing 17% to 55% of inputs on an average weekly basis (from on and off-glacier areas) (Fig. 3). Its largest contribution occurs in mid-June (55%), decreasing as the dry season progresses, while remaining relatively stable over the wet season (23% on average) (Fig. 3A). On the Blanca (eastern) side of the catchment, snowmelt is the second highest contributor to catchment inputs (36% of Blanca flows at the outlet) after rainfall (51%) over the year. In the dry season, however, it becomes more important than rainfall (44% and 31% of Blanca flows at the outlet are from snow melt and rain, respectively) (Fig. 4). Contributions change with distance downstream: dry season snow melt is more important than rainfall on the Blanca side between 0 and 80 km from the outlet, while higher upstream snow melt totals are smaller than rain inputs (Fig. 4). On the Negra (western) side of the catchment, snow melt provides less water than rain in both seasons (11% and 23% of Negra flows at the outlet in the wet and dry season, respectively), due to lower maximum elevations. Snowmelt remains important with distance down the main stem of the Rio Santa due to the basin configuration, since high-elevation tributary catchments continue to provide meltwater to the main stream.

The contribution of ice melt to the Rio Santa catchment inputs peaks in early August, reaching 44% of the inputs, and remaining at >30% during most of the dry season (from early July to mid September) (Fig. 3A). Crucially, ice melt is important in the dry season in all glacierised catchments, since those with a smaller glacier area have lower overall dry season flows. For instance, the Querococha catchment has 1% glacier cover, but the ice melt contribution is >40% for 6 weeks in the mid-dry season (Fig. 3G). However, ice melt is a minor contributor in the wet season (2% on average over the Rio Santa catchment) due to the relatively large quantity of rainfall and because glaciers are often snow-covered. For these reasons, ice melt remains a small contributor in the wet season even in the most heavily glacierised catchments (e.g., the wet season contribution is 12% for Cuchillacocha with 52% glacier cover, Fig. 3B). Spatially, ice melt is a substantial proportion of the inputs into the Blanca side of the catchment in the dry season (24% at the outlet) (Fig. 4c, f). Similar to the pattern for snowmelt, the importance of ice melt on the eastern (Blanca) side increases with distance downstream due to the continued joining of glacierised catchments to the main stream.

In the wet season, runoff inputs originate from both sides of the catchment, although the highest specific discharges are from the Blanca side (Fig. 4b). In the dry season, however, there is a clear divide between the lack of runoff on the non-glacierised Negra side and the much higher contribution from the glacierised Blanca side (Fig. 4c), demonstrating that snow and ice melt from high elevations is vital for dry season discharge.

## Discussion

Our physically based and spatially resolved modelling approach has allowed us to quantify the snow dynamics in the upper Rio Santa catchment and contrast their role and importance with those of ice melt. Above ~5000 m a.s.l. (10.5% of the catchment area) the snowpack plays a seasonal storage role, providing meltwater in the early dry season (see Results). Crucially, however, a large proportion of the snowpack <5000 m a.s.l. is thin and ephemeral, often with evening snowfall being melted in the next day or two (SI Fig. 15). Ephemeral snow has been classified by refs. ^34–36^ as having a continuous duration of less than 60 days and a depth <0.5 m. Analysis of modelled daily snow cover (SI Fig. 18) shows that the majority of the snow cover in the catchment is ephemeral by this definition, with substantial areas of seasonal snow ( > 120 days, >1.5 m^37^) only existing above 5000 m a.s.l. There is a clear transitional area between 4800 m a.s.l. and 5400 m a.s.l., where the snow depths and durations increase sharply. Importantly, the majority of the ephemeral snow in the catchment (below 4700 m a.s.l.) has less than half the duration and depth of the definition, suggesting that snow packs in the Peruvian Andes are particularly dynamic, and that a finer differentiation of ephemeral snow types may be required. Additional observations of such processes with at least a daily temporal resolution (e.g., timelapse photography^38^ or near-surface temperature measurements^39^) would be beneficial in confirming the existence and characteristics of ephemeral snow in tropical regions.

In previous studies, the off-glacier snowpack was not thought to be important^18^, but we show here that it provides a substantial contribution to the water inputs into the catchment (Fig. 2). Given the large catchment area over which this temporary snowpack is formed and melted (52% of the catchment lies between 4000 and 5000 m a.s.l.), it might alter the catchment energy and water budget. For instance, snow cover controls surface albedo and acts to protect glacier ice and soil, with snowmelt consuming energy that would otherwise be used for other processes (e.g., ground surface heating, evapotranspiration). These snow-atmosphere interactions can result in complex feedbacks on climate and hydrological processes^40–42^. For instance, the direct impact of snow on surface albedo changes the surface energy balance, in turn altering the air temperature^40,41^.

Differences in infiltration of snow melt compared to rain can also lead to impacts on soil moisture and hence on the partitioning of water into runoff and evapotranspiration^42^, as well as their subsequent hydrological pathways. Previous work has suggested that snow melt is more effective at recharging groundwater than rainfall^43,44^, but understanding of snowmelt recharge is concentrated in regions with seasonal snowpacks. Current findings specific to ephemeral snow are contradictory: in a Mediterranean climate Nadal-Romero and López-Moreno^35^ found infiltration of ephemeral snow events was lower than from rain, with shorter flood hydrograph lag times, related to frozen soil and impermeable substrates; whereas in Arizona, Dwivedi et al.^45^ found increased soil percolation at ephemeral snow sites, compared to seasonal sites, due to melt occurring in mid-winter when evapotranspiration is lower. Disentangling the impact of ephemeral snow on the hydrograph from our modelling in the Rio Santa catchment is complicated, since the large catchment elevation range results in precipitation events being composed of both snow and rain simultaneously at different elevations. Understanding the impact of snow melt on soil moisture and recharge in regions of intermittent snowpacks and warm-season snow accumulation is lacking^46^, and this calls for more research to investigate these processes.

We have also isolated the contributions of ice and snow melt to total inputs into the Rio Santa catchment, going beyond previous water balance modelling and hydrochemical mixing model studies (e.g., refs. ^7,8,19^). It is therefore pertinent to compare our results to previous studies to assess the similarity of melt contributions from physically based modelling compared to other methods. Within these comparisons, our modelled cryospheric contributions are calculated as a fraction of the inputs into the hydrological system, including either all ice and snow melt over a given catchment area, or only the ice and snow melt over glacier areas within that catchment (in parentheses).

Crucially, we find that our estimates of ice and snow melt as a fraction of total inputs are higher than previous water balance modelling and hydrochemical mixing model studies. We find that 25% (6% on-glacier) of the upper Rio Santa wet season discharge is composed of ice and snow melt, and 65% (40% on-glacier) in the dry season, with the annual inputs from ice and snow melt equating to 45% (23% on-glacier). This compares with an estimate of ~12% glacier contribution annually in 1998/99 by Mark and Seltzer^7^ and ~6–10% over 1971–2000 by Buytaert et al.^9^. Our on-glacier estimates are more similar to values from conventional water balance modelling since off-glacier snow melt would tend to be included within precipitation for those methods^7^. Our higher values compared to Buytaert et al.^9^ may be because their study assumed glaciers to be in equilibrium, whereas we simulate glacier mass loss and corresponding glacier area change, reflecting the current imbalance of glaciers in the region^16^. However, our results are much more similar to the only other physically based modelling approach in the region. Recently, Aubry-Wake et al.^28^ simulated the runoff within the Casa de Aqua sub-catchment using the Cold Regions Hydrological Modelling Platform, and the contribution of snow and ice melt as a proportion of inputs equated to 67% annually, almost the same as found in our model for this domain (69%). This confirms that our higher cryospheric contribution to catchment inputs compared to past approaches across the Rio Santa catchment is likely more realistic. This improves our perspective on the importance of ice and snow melt to runoff in the region and supports the use of advanced modelling tools appropriate to its complexity.

Despite the agreement of our modelling results with those of other physically based models, and a thorough model evaluation (see ‘Methods’), there are processes that TOPKAPI-ETH does not fully resolve and which may be important in the representation of cryospheric processes in tropical regions. In particular, the discrimination of the precipitation phase is based on a single constant threshold. We calibrated this threshold against a dynamic scheme which takes into account elevation and humidity^47^ at all the WRF grid cells, with the catchment median value applied in the model (SI Section 1.3.1). Since modelled snow melt amounts are sensitive to this parameter (see Methods) we conducted additional model runs with the extremes of the calibrated values (SI Fig. 14). This variation in the precipitation threshold temperature (between 1.2 °C and 4.9 °C) results in the fraction of snow melt of total inputs for the upper Rio Santa varying by −6% to +18% compared to the standard run. Differences are greater in the wet compared to the dry season since the magnitude of dry season snow melt is determined by melt rates rather than the amount of snowfall. The fraction of ice melt of total inputs is insensitive to variations in the precipitation threshold temperature (fractions differ by −0.05% to +0.07% on average for the upper Rio Santa) since the magnitude of ice melt is affected mostly during the wet season when it is a small proportion of catchment inputs.

TOPKAPI-ETH uses an Enhanced Temperature Index (ETI) approach^48^ to modelling snow and ice melt, with the parameters calibrated based on comparison with energy balance modelling at the point scale (see Methods and SI Section 1.3.2). Given the good transferability of these parameters across sites in the ablation zone and the good correspondence of modelled and measured glacier mass balances (Fig. 1c) we have confidence that the melt amounts are accurate in glacier ablation zones. However, the model does not include mass loss by sublimation, which is likely important at the highest elevations^10,49,50^ nor can it explicitly account for the variable impact of the snow or ice pack cold content on melt rates^29,50^, although these processes were included in the energy balance model used for calibration. It also cannot represent processes such as the effect of the ground heat flux and horizontal energy transfers between bare ground and snow on snow melt rates, which may be important in off-glacier areas if snow is patchy^51^. Nevertheless, the balance in TOPKAPI-ETH of including most cryospheric processes while remaining parsimonious allowed modelling of the Rio Santa catchment at a temporal and spatial resolution that has not been attempted before.

**Fig. 5:**
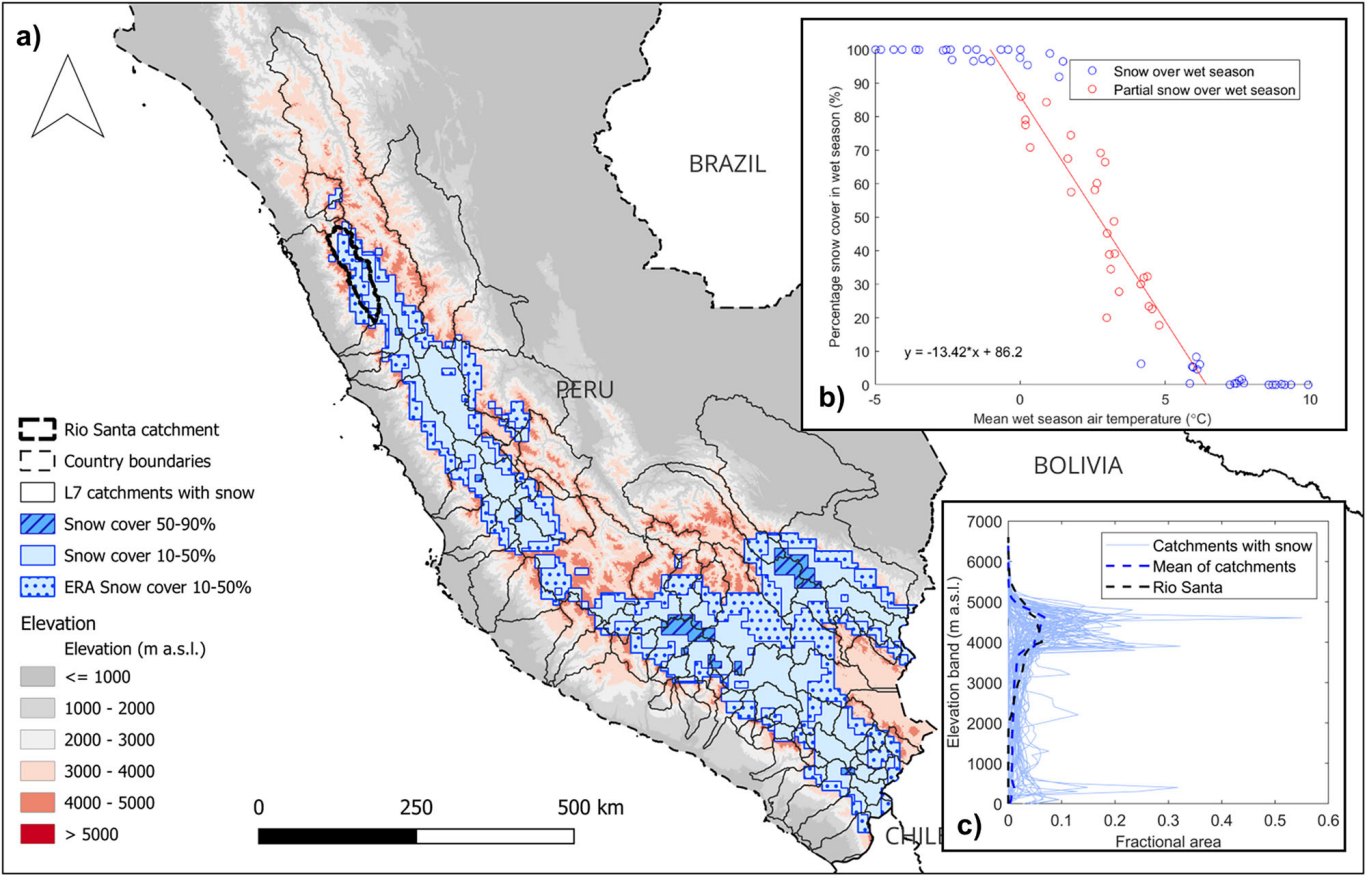
The potential importance of snow across the Peruvian Andes. **a** Areas of snow cover (light blue for 10–50%, dark blue diagonal hatch for 50–90%) as determined from the relationship shown in b) applied to the wet season air temperatures from ERA5-Land^69^ reanalysis (see SI Section 1.6 for methods). We also show the wet season percentage snow cover directly from ERA5-Land reanalysis for comparison (light blue with dots, values are all <50%). Black solid outlines are the HydroSHEDS^68^ level 7 catchments which intersect the snow-covered areas, with the bold dashed line the upper Rio Santa catchment. The elevation data (red to grey) originates from ref. ^68^. **b** The relationship (red line) of modelled wet season air temperature to modelled wet season percentage snow cover, red dots show points used in the relationship (where snow cover was between 10 and 90%), with the blue dots showing points which were not used. **c** Catchment hypsometries, with the HydroSHEDS catchments shown in (**a**) as light blue lines, with the mean in blue dashes, and the modelled upper Rio Santa catchment in black dashes.

An important consideration is the relevance of our results across Peruvian high-elevation catchments. The upper Rio Santa catchment has a similar hypsometry to many of the high-elevation basins that descend from the Peruvian Andes (Fig. 5c). Elevation is key in determining the existence of the ephemeral snow, and the distribution of area with elevation dictates the hydrological importance of this short-lived snow. We reconstructed the area in the mountains of Peru that can sustain ephemeral snow (Fig. 5a) by deriving the temperature range corresponding to modelled ephemeral snow conditions (Fig. 5b, SI Section 1.6). This analysis shows that conditions conducive to ephemeral snow are prevalent across the Peruvian Andes, especially along the Cordillera Occidental to the west and the southern limb of the Cordillera Central. This suggests that meltwater from short-lived wet season snow cover could be important to a large proportion of Peruvian basins (covering 36% of the land area of Peru), and that high spatial and temporal resolution modelling is now required to understand exactly the contributions of snowmelt, especially given the stark precipitation gradients across the country. Detailed process studies are needed to understand the consequences of tropical ephemeral snow (as opposed to rain) for infiltration, groundwater recharge, and runoff generation.

Given the importance of ice and snow melt for runoff in the Rio Santa catchment, and potentially across the Peruvian Andes, the impact of climate change on cryospheric systems will likely alter future runoff regimes. Climate change alters the snow and ice melt dynamics in distinct but compounding ways, thus also altering the seasonal hydrographs of mountain catchments. Cryospheric processes in the Peruvian Andes are especially vulnerable to air temperature increases because of the summer-accumulation regime, where glacier ablation zones are close to melting conditions for most of the year, and feedbacks between precipitation phase, surface albedo, and melt rates are strong^11^. Warmer temperatures will shift the phase of precipitation towards more rain^52^, reducing the snowpack thickness, decreasing the surface albedo, and increasing glacier melt^10^. The very thin snow cover of elevations <5000 m a.s.l. (see ‘Results’) are especially vulnerable to increasing temperatures. An increase in the liquid fraction of precipitation, combined with the occurrence of more extreme rainfall events^17^, could also result in higher peak flows. Reduction of the high-elevation seasonal snowpack (>5000 m a.s.l., Fig. 2a) will reduce early dry season runoff when snowmelt inputs currently peak. July and August flows in glacierised catchments would be further reduced by a loss of glacier ice, a substantial contributor at this time of year (Fig. 3).

The ability for seasonal snow and glacier ice to buffer low flows in dry periods differs: seasonal snow can compensate for dry periods with a limited capacity, based on the wet season accumulation, whereas glaciers can contribute to dry period runoff on multi-annual timescales^53,54^. However, eventually this buffering ability will reduce as glaciers shrink, and quantification of when this reduction will occur is key for understanding the future of Andean water resources. Snow and ice melt recharge groundwater in this region, which is an important source of dry season flows^21,55,56^. Therefore, loss of the snow and ice contribution may also reduce discharge from high-elevation springs.

## Conclusions

The high temporal and spatial resolution of the glacio-hydrological modelling in this study has allowed a thorough understanding of the snow dynamics, and in turn, a renewed quantification of the contributions of snow and ice melt to catchment inputs within the upper Rio Santa catchment, Peruvian Andes. Surprisingly, we found that the snow dynamics are characterised by a highly ephemeral snowpack at elevations below ~5000 m a.s.l. Here, the snowpack is thin and melts in hours to days, with the snowpack duration and thickness increasing with elevation. Combined with the large catchment area at these elevations, the resulting snowmelt volume is large, highlighting a process and volumetric contribution to catchment inputs that was previously neglected. Above those areas, snow can accumulate during the wet season, providing a seasonal contribution to the hydrological system in the early dry season. Overall, we show that snow melt is a vital source of water all year (17% to 55% of the inputs to the upper Rio Santa catchment), with its contribution peaking in early June. Ice melt is an important contributor in the dry season and on the Blanca side of the catchment. Crucially, the fractional contribution of ice melt in the dry season can be substantial, even in catchments with a small glacier cover. We estimate higher melt contributions to catchment inputs than simpler modelling approaches, suggesting that accurately representing the complex dynamics of snow and ice melt in glacio-hydrological models in this region is vital. Snow and ice melt exhibit seasonally and spatially distinct dynamics, and their functioning and hydrological role will be modified in distinct ways under future climate change.

## Methods

### Overview

We apply the TOPKAPI-ETH model over the upper Rio Santa basin (4953 km^2^, Fig. 1a) comprising the glacier-covered Cordillera Blanca to the east and the glacier-free Cordillera Negra to the west. The model is run at an hourly timestep, with a grid size of 100 m, over a 3-year spin-up period (1st November 2005 to 31st October 2008) followed by a 10-year analysis period (1st November 2008 to 31st October 2018). The model includes an ETI approach for snow and ice melt^48^, and a Debris Temperature Index approach^57^ for ice melt beneath debris. The evolution of snow albedo^58^ and snow avalanche processes^59^ are also included. Melt and precipitation occurring within glacierised grid cells are routed using a linear reservoir approach^60^. Glacier geometry evolves over time following the Huss et al.^61^ empirical parameterisation calibrated using remotely sensed glacier elevation change data. Evapotranspiration is calculated using the Priestly-Taylor approach and applied to different land covers based on crop coefficients. Water is routed over the surface, within the soil, and through the groundwater layers to generate stream runoff^29^. An overview of the model forcing, calibration and evaluation is given in SI Fig. 1. We calculate the importance of ice and snow melt to catchment inputs as fractions of the total inputs into the system, with the inputs defined as the sum of the ice melt, snow melt (on and off-glacier, unless otherwise stated) and rain amounts over a given area.

### Model inputs

TOPKAPI-ETH requires inputs of near-surface air temperature, precipitation, and cloud cover transmissivity. These were derived from hourly outputs from a 4 km WRF atmospheric model simulation forced by ERA5 reanalysis, provided by Potter et al.^17^. Quality-checked data from 35 precipitation and 26 temperature stations were used to bias-correct the WRF outputs (see Potter et al.^17^ and Fyffe et al.^10^ for details). Incoming shortwave measurements at five sites were used to bias-correct the cloud cover transmissivity values. The downscaling of the WRF inputs to the 100 m grid cells and details of the spatial inputs are documented in SI Section 1.2 and SI Table 1.

### Parameter derivation

TOPKAPI-ETH parameter values were determined using a multi-step, multi-variable approach to reduce equifinality, similar to Ragettli and Pellicciotti^29^, Ragettli et al.^32^, and Ayala et al.^31^ (SI Section 1.3). The air temperature threshold for partitioning precipitation into solid and liquid form was established via comparison against values calculated with the more sophisticated Ding et al.^47^ approach (SI Section 1.3.1). ETI melt parameters were derived from comparison with energy balance simulations^10^ at five on-glacier weather stations (SI Section 1.3.2), with albedo measurements on Artesonraju Glacier used to calibrate the albedo parameterisation of Brock et al.^58^ (SI Section 1.3.3). The retention factors for the snow and ice reservoirs were calibrated using discharge measurements from Casa de Aqua gauging station (Fig. 1a, SI Section 1.3.4). The glacier evolution parameters were derived from geodetic measurements of elevation change from 2000–2020 by Hugonnet et al.^62^ (SI Section 1.3.5). After the derivation of all other snow and glacier parameters, we then calibrated the air temperature reduction over clean glacier surfaces. This was achieved by calibrating the modelled sub-catchment wide glacier mass balances against the Hugonnet et al.^62^ dataset from 2010-2020 (SI Section 1.3.6). Details of the parameterisation of evapotranspiration, soil properties, and lakes are provided in SI Section 1.3.

### Observations used for model evaluation

The model was evaluated against ground and remotely sensed data and stream discharge. Snow cover was assessed against snow-line altitudes from MODIS (Moderate Resolution Imaging Spectroradiometer) and snow cover derived at three point locations from albedo measurements (two on- and one off-glacier) (SI Section 1.4.1). Glacier mass balance was compared against altitudinally resolved mass balances for each glacier (following Miles et al., 2021) for the period 2015-2019 (SI Section 1.4.2), allowing comparison of measured and modelled mass balance profiles and equilibrium-line altitudes. Field data of ablation and snow accumulation at Artesonraju, Shallap, and Gueshgue glaciers were also compared with the model. We additionally show the comparison of glacier mass balances from geodetic elevation change data with the model outputs, for the periods 2010–2015, 2015–2020, and 2010–2020, although the 2010-2020 data were used to calibrate the temperature decrease applied over glacier ice^62^. The modelled runoff was compared with discharge data from five gauging stations^63^ (SI Table 5).

### Model skill in simulating snow and glacier processes

The modelled snowlines are verified through good correspondence with MODIS data, with a small bias of 24.3 m and an RMSE of 140 m (Fig. 1d and SI Section 2.1.3). The model does tend to overestimate the snowline elevation in the dry season and underestimate it in the wet season, but it replicates the multi-annual patterns of snowline evolution well. Modelled snowlines are influenced by the choice of SWE threshold used to define a cell as snow-covered (represented by the error bars in Fig. 1d), but the magnitude of the uncertainty is similar to that in the MODIS data itself (defined by the Normalised Difference Snow Index threshold). We tested the influence of the threshold temperature between snow and rain on the modelled snowlines (SI Fig. 11), finding that differences were substantial only in wet season months and that the chosen threshold of 2.2 °C performed best. The model is also able to replicate the snow cover behaviour shown by the albedo observations at three locations (SI Fig. 12 and SI Table 7). The ability of the model to replicate melt processes sufficiently is demonstrated by its close replication of the catchment-scale mass balance profile, derived from remotely sensed glacier elevation change and velocity data (Fig. 1c). This is especially true >4700 m a.s.l., where the bias in the comparison of the measured and modelled mass balance profiles is only 0.07 m w.e. a^-1^. The bias over the whole profile is larger (−0.31 m w.e. a^-1^), likely due to uncertainties in modelling sub-debris ablation. The correspondence of modelled and measured sub-catchment mass balance profiles as well as with field data from three glaciers (SI Figs. 7 and 9) further confirm that modelled melt amounts are reasonable across the catchment. Comparison with independent discharge measurements lends confidence to the model’s ability to replicate all the main hydrological processes (SI Fig. 10 and SI Section 2.1.2).

### Assessing model sensitivity

We assess the model sensitivity to the snow and ice parameters for one hydrological year, changing one parameter at a time, following Shaw et al.^64^ (SI Section 1.5). The modelled discharge and melt values are most sensitive to variations in the threshold temperature to allow melt (*TT*), the threshold temperature between snow and rain (*PrecSF*), and the temperature decrease over clean glacier areas (*Tmod*) (SI Section 2.1.4 and SI Fig. 13). The *TT* value was calibrated alongside the other melt parameters through comparison with an energy balance melt model, with the chosen parameter set shown to be the most transferable across four of the sites (SI Section 1.3.2). The calibrated *PrecSF* value used for the model runs shown (2.2 °C) is suitable for elevations ~4500 m a.s.l. (SI Fig. 5). Due to the lower air pressure and thinner air at higher elevations, the threshold temperature for snowfall increases^47^, therefore, at higher elevations we would model slightly lower than expected snowfall and vice versa at lower elevations. Given that most snowfall occurs above 4500 m a.s.l. our snowfall estimates are likely conservative overall. Since snow melt amounts were particularly sensitive to *PrecSF* we conducted additional full model runs with the maximum and minimum calibrated values (4.9 °C and 1.2 °C), with the impact of this parameter range on modelled melt and rain contributions to catchment inputs considered in the Discussion. Melt amounts are sensitive to *Tmod* since it controls air temperatures over glacier areas. *Tmod* was calibrated for each sub-catchment after all the other snow and ice parameters were defined (SI Section 1.3.6). Given its importance, we would welcome further work to quantify and parameterise glacier cooling effects^65^.

## Supplementary information

Transparent Peer Review file

Supplementary Material

## Data Availability

The outputs of the TOPKAPI-ETH glacio-hydrological model generated during this study are available at https://zenodo.org/records/15297284.

## Code availability

The forcing data and TOPAKPI-ETH model code required to generate the modelled outputs are available at https://zenodo.org/records/15301957, and the MATLAB code (version R2022b) required to analyse the model outputs and produce the figures in the paper is available at https://zenodo.org/records/15341457.
