# Supplementary material for: Thin and ephemeral snow shapes melt and runoff dynamics in the Peruvian Andes
Source: Commun Earth Environ. 2025 Jun 5;6(1):434. doi: 10.1038/s43247-025-02379-x (PMC12141031; doi:10.1038/s43247-025-02379-x)
Supplement: Supplementary file 2 — Supplementary Material [file 43247_2025_2379_MOESM2_ESM.pdf]

# Supplementary Information

## Thin and ephemeral snow shapes melt and runoff dynamics in the Peruvian Andes

Catriona L. Fyffe[1,2], Emily Potter[3,4], Evan Miles[5,6,7], Thomas E. Shaw[2], Michael McCarthy[2,5], Andrew Orr[8], Edwin Loarte[9], Katy Medina[9], Simone Fatichi[10], Rob Hellström[11], Michel Baraer[12], Emilio Mateo[13], Alejo Cochachin[14], Matthew Westoby[15], Francesca Pellicciotti[2]

1 Department of Geography and Environmental Sciences, Northumbria University, Newcastle upon Tyne, UK

2 Earth Science, Institute of Science and Technology Austria, Klosterneuburg, Austria

3 Department of Atmospheric and Cryospheric Sciences, University of Innsbruck, Innsbruck, Austria

4 School of Geography and Planning, University of Sheffield, Sheffield, UK

5 Swiss Federal Institute for Forest, Snow and Landscape Research, Zürich, Switzerland

6 University of Zürich, Zürich, Switzerland

7 Department of Geosciences, University of Fribourg, Fribourg, Switzerland

8 British Antarctic Survey, Cambridge, UK

9 Universidad Nacional Santiago Antúnez de Mayolo, Huaraz, Peru

10 Department of Civil and Environmental Engineering, National University of Singapore, Singapore

11 Bridgewater State University, Bridgewater, MA, USA

12 École de technologie supérieure, Université du Québec, Montréal, Canada

13 Pacific Institute, Oakland, CA, USA

14 Autoridad Nacional del Agua, Huaraz, Peru

15 School of Geography, Earth and Environmental Sciences, University of Plymouth, Plymouth, UK

# 1 Supplementary Methods

## 1.1 Modelling overview

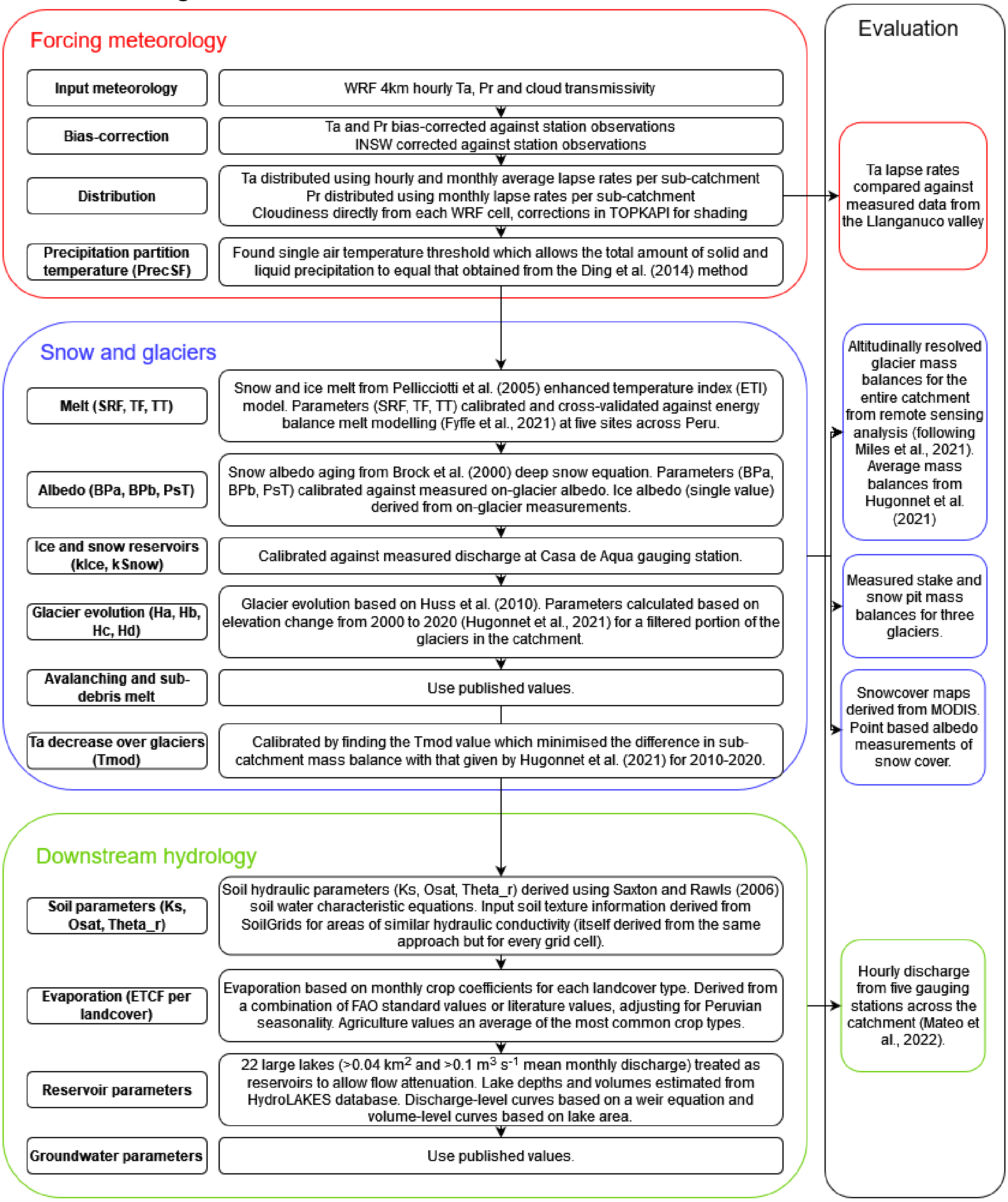


*Supplementary Figure 1 Modelling workflow.*

## 1.2 Model inputs

### 1.2.1 Air temperature, precipitation and cloud transmissivity downscaling

The WRF-based meteorological inputs (4 km resolution) require further downscaling to the 100 m TOPKAPI-ETH model grid cells. The catchment is split into 28 sub-catchments (see Figure 2), and the WRF grid cells within each are used to calculate the mean near-surface air temperature and precipitation lapse rates with elevation. Air temperature lapse rates are calculated for every hour and month and are based on the gradient of the linear relationship between the mean air temperature at each of the WRF cells within the sub-catchment and elevation, excluding WRF grid cells assigned as on-glacier (since the influence of glaciers on air temperature is dealt with separately). Hourly air temperature lapse rates from WRF compared well with those calculated from eight temperature sensors within the Llanganuco sub-catchment (Section 1.2.2). Precipitation lapse rates are calculated monthly and based on the precipitation sum at each WRF grid cell divided by the precipitation sum at the main station in each sub-catchment. This gives a multiplier which can be related to the difference in the cell elevation and then the correct multiplier for each TOPKAPI-ETH cell can be calculated. Sub-catchments were delimited to be hydrologically correct (boundaries follow hydrological watersheds), include at least 5 WRF cells to allow the calculation of lapse rates and as far as possible to separate the eastern (Blanca) and western (Negra) sides of the catchment.

The hourly forcing for air temperature and precipitation for TOPAKPI-ETH is taken from the WRF grid cell closest to the sub-catchment centroid, with the appropriate lapse rates applied to derive the air temperature and precipitation for each model grid cell within the sub-catchment. Cloud transmissivities (see Pellicciotti et al., 2005) were calculated as the ratio between the WRF incoming shortwave radiation at the top of the atmosphere and that near the surface. Comparison with measured incoming shortwave radiation values from five in-situ stations within the catchment suggested that WRF overestimated the cloud transmissivity, especially in the morning, and so mean correction factors were applied depending on the time of day and whether precipitation occurred or not (Section 1.2.3). WRF-based cloud transmissivities are not downscaled further since the main local controls on the incoming shortwave radiation at a given grid cell are the aspect and shading of each cell, which is calculated using algorithms within TOPKAPI-ETH.

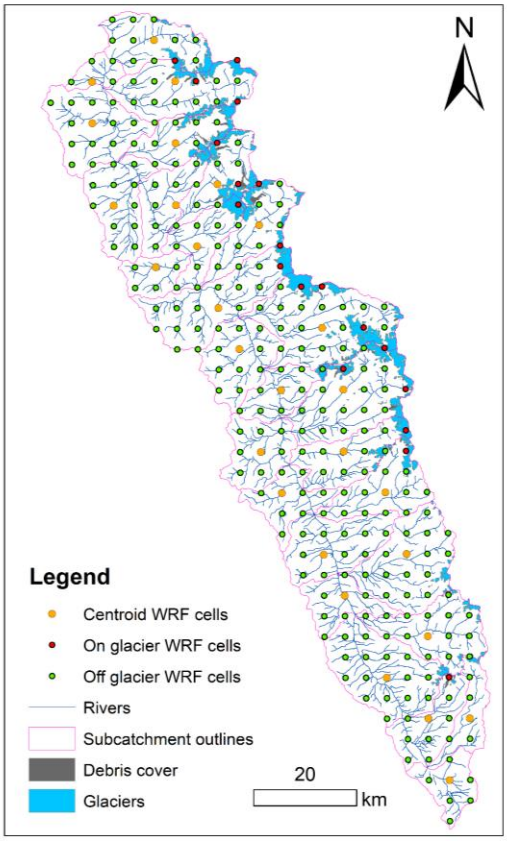


*Supplementary Figure 2 Distribution of meteorological variables using sub-catchments for the upper Rio Santa catchment. The points represent the centre of the WRF grid cells, with the value from the centroid cell of each sub-catchment used for the forcing of air temperature and precipitation for the TOPKAPI-ETH model. Note that the WRF cells were defined as being on glacier if they were within the RGI glacier outlines at the WRF (4 km) resolution so this may not correspond with the glacier areas in TOPKAPI-ETH, but this was accounted for in the forcing methodology.*

### 1.2.2 Comparison of WRF and measured air temperature lapse rates

To check that the WRF near-surface air temperature lapse rates are reasonable they are compared with lapse rates calculated from data measured within the Llanganuco sub-catchment. Here Hellström et al. (2017) installed a network of eight air temperature sensors at varying elevations within the catchment. The sensors have complete records for around 25 days in June/July and December 2005. The hourly average lapse rates within these periods were calculated using both the data from the sensors and the WRF points within this sub-catchment (off-glacier only) and compared.

In the wet season the air temperature lapse rates measured by the sensors in the Llanganuco valley compare very well with the WRF lapse rates (Figure 3), with almost no difference in the mean lapse rate (both equal -0.0059°C $m^{-1}$) and a close similarity in the diurnal amplitude of hourly lapse rates (the RMSE between the hourly average lapse rates is 0.0012°C $m^{-1}$). In the dry season the mean lapse rate is slightly steeper in WRF compared to measured (-0.0061°C $m^{-1}$ for WRF compared to -0.0053°C $m^{-1}$ measured), although the main difference is that the diurnal amplitude of the lapse rate signal is smaller in WRF than measured. These differences could be explained by the spatial distribution of the measured sensor network, or because the coarser representation of topography in WRF (with a resolution of 4 km) means it cannot represent well the near surface up-valley wind which

likely occurs in this valley (Hellström et al., 2017). Overall though this suggests that WRF is generally able to replicate the diurnal and seasonal pattern of air temperature lapse rates.

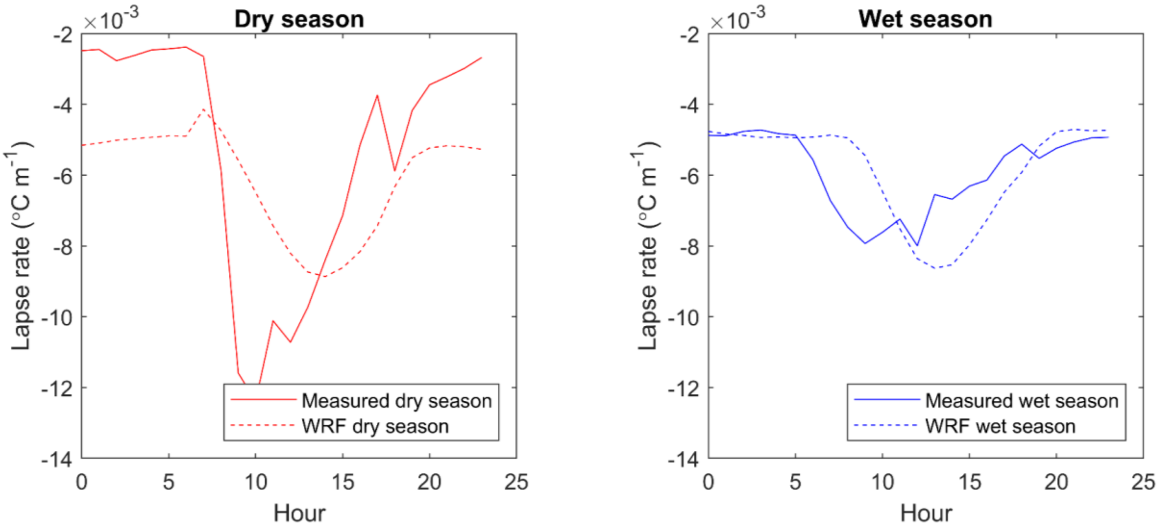


*Supplementary Figure 3 Comparison of near-surface air temperature lapse rates calculated from measurements (Hellström et al., 2017) and from WRF within the Llanganuco valley.*

### 1.2.3 Correction of cloud transmissivity using in-situ data

In TOPKAPI-ETH the cloud cover transmissivity (or cloud factor, Pellicciotti et al., 2005) ($\tau_c$) is applied as a multiplier to the calculated clear sky global irradiance to give the actual global irradiance for each grid cell, where $\tau_c$ can theoretically vary between 0 (completely overcast) and 1 (clear-sky conditions). $\tau_c$ is calculated as the actual incoming radiation at the surface divided by the global clear sky radiation. Values of $\tau_c$ were calculated from the WRF outputs as the ratio between the WRF incoming shortwave radiation near the surface and that at the top of the atmosphere. We compared the WRF cloud cover transmissivity values to those calculated from incoming shortwave measurements at five stations across the catchment (Artesonraju Glacier (AG), Artesonraju Carac Moraine (ACM), Shallap Glacier (SG), Shallap Moraine New (SMN) and Cuchillacocha Quilcay (CQ)). Details of all the stations are given in Fyffe et al. (2021). Measured $\tau_c$ was calculated as the measured incoming shortwave radiation at these stations divided by the global irradiance calculated for the TOPKAPI-ETH grid cell occupied by the station. We also calculated the relationship between incoming longwave radiation and $\tau_c$ values at the three stations with available data (Artesonraju Glacier, Shallap Glacier and Shallap Moraine New). Between 9 am and 4 pm there were strong relationships between incoming longwave radiation and $\tau_c$, suggesting that within these times the $\tau_c$ values should be representing cloudiness rather than shading or low sun angles at the stations.

Upon comparing the WRF and measured values of $\tau_c$ it was apparent that the WRF values were higher compared to the measurements, especially in the morning, indicating that WRF simulates not enough cloud or cloud that is optically too thin. To correct this we calculated the average hourly difference between the WRF and station values between 9 am and 4 pm, and separately for hours with and without precipitation (using the WRF modelled precipitation for consistency). We then took the average of the differences across the five stations, applying the value at 9 am to the hours before, and the value at 4 pm to the hours after (Figure 4). These average differences were then used to correct all the WRF values before they were input into TOPKAPI-ETH, based on the hour and whether there was precipitation or not. Although there are differences in the correction values calculated at

each station the diurnal pattern was consistent across all the sites, giving us confidence that the corrections were suitable to be applied across the catchment.

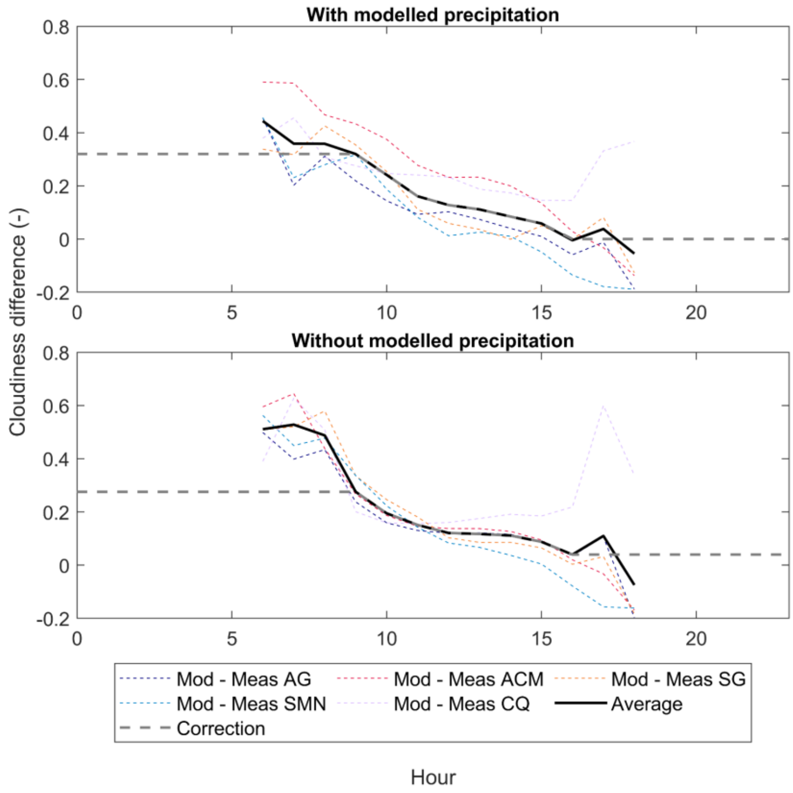


*Supplementary Figure 4 Differences in cloud cover transmissivity ($\tau_c$) between WRF and measurements at five meteorological stations within the Rio Santa catchment. The mean of the differences at all the stations between 9 am and 4 pm (grey dashed) was used to correct the WRF values.*

### 1.2.4 Input gridded data

*Supplementary Table 1 Source of data used for TOPKAPI-ETH input grids.*

| **Data type** | **Source** |
|---|---|
| Digital elevation model | ALOS PALSAR from 2000 with 12 m native resolution, resampled to 100 x 100 m grid cell size. |
| Glacier and debris-cover outlines | INAIGEM (2018) Peruvian Glacier inventory. |
| Ice thickness | Farinotti et al. (2019) with extrapolation where necessary to fill glacier outlines. The extrapolation is based on a minimum annulus approach with gradually increasing size so that the glacier thickness does not increase around the glacier edges. |
| Debris thickness | Derived from thermal satellite imagery (Landsat 8) and a nonlinear energy balance model (following the method of Rounce and McKinney, 2014), with ERA5 used as forcing. |
| Landcover | Based on Mapa Nacional de Cobertura Vegetal (Ministerio del Ambiente, 2015) but with the glacier areas replaced by the 'Little to no vegetation' category since they are dealt with separately. |
| Soil classes | Soil classes are based on the SoilGrids 250 m v2.0 dataset (Poggio et al., 2021). To derive the classes the sand, clay and soil organic carbon layers were downloaded for all depths and then averaged vertically into two layers (0-0.3 m and 0.3-2.0 m). The soil hydraulic properties were calculated following soil water characteristic equations (e.g. Saxton and |

| | |
|---|---|
| | Rawls, 2006). The calculated soil hydraulic conductivity values for the upper layer were then reclassified into 10 classes using a quantile method and these were used as the soil classes for the model. |
| Soil depth | SoilGrids 250 m v2.0 Depth to Bedrock layer (Poggio et al., 2021). Two soil layers are used in the model, the top layer was 0.3 m deep, and the lower layer was the total soil depth – 0.3 m. |
| Initial snow depth | Initial snow depths were derived from measured stake and snow pit data from four glaciers (Artesonraju, Shallap, Yanamarey and Guesgue) collected by Autoridad Nacional del Agua (ANA). Annual mass balance profiles were constructed for each year of available data, which were then averaged for each glacier and again to give an average profile for the catchment. The mass balance gradient above the equilibrium line altitude (ELA) was extrapolated to allow the snow depths to be calculated for all elevations above the mean ELA (4975 m a.s.l.). Off glacier snow was only included above 5731 m a.s.l., based on remote sensing analysis of off-glacier dry season snow line elevations (Fyffe et al., 2021) and snow depths were reduced by a reduction factor on steep slopes (0% reduction at 30° to 100% reduction at 60°). |

## 1.3 Parameter calibration

*Supplementary Table 2 Overview of the parameters used within TOPAKPI-ETH.*

| Parameter type | Name | Value | Description | Derivation |
|---|---|---|---|---|
| Temperature | Tmod | Calibrated per subcatchment | Air temperature decrease over clean ice | See Section 1.3.6 |
| Temperature | Tmod debris | 0.3°C | Air temperature increase over debris-covered ice | Ayala et al. (2016) |
| Precipitation | PrecSF | 2.2°C | Threshold air temperature to differentiate between solid and liquid precipitation. | See Section 1.3.1 |
| ETI melt | SRF, TF, TT | 0.0067 $m^2$ mm $W^{-1}$ $h^{-1}$ °C, 0.0181 mm $h^{-1}$ °C, 1.2547°C | Shortwave radiation factor, temperature factor, temperature threshold (Pellicciotti et al., 2005) | See Section 1.3.2 |
| DETI melt | TFd1, TFd2, SRFd1, SRFd2, lag1, lag 2 | 0.0170 mm $h^{-1}$ °$C^{-1}$, 0.4043, 0.0114 mm $m^2$ $h^{-1}$ $W^{-1}$, 44.2 $m^{-1}$, 36 h $m^{-1}$, 1 h | Temperature factor for debris 1, temperature factor for debris 2, shortwave radiation factor for debris 1, shortwave radiation factor for debris 2, lag 1 and lag 2 (Carenzo et al., 2016) | Parameters as calibrated by Ayala et al. (2016). |
| Snow albedo | $BP_a$, $BP_b$, PsT | 0.75, 0.115, 1 mm $h^{-1}$ | Fresh snow albedo, snow albedo decay, threshold precipitation to reset albedo (Brock et al., 2000) | See Section 1.3.3 |
| Clean ice albedo | $\alpha_{glacier}$ | 0.3007 | Clean ice albedo | From measured albedo at Artesonjaru Glacier |
| Debris albedo | $\alpha_{debris}$ | 0.18 | Debris-covered ice albedo | Unpublished albedo measurements at Pirámide glacier, Chile. |
| Glacier reservoirs | $k_s$, $k_g$ | 1184.2, 33.86 | Snow retention factor, ice retention factor | See Section 1.3.4 |
| Glacier evolution | $H_a$, $H_b$, $H_c$, $H_d$ | -0.1307, 0.1258, 0.1483, 2 | Parameters a to d in Δh parameterisation of Huss et al. (2010). | See Section 1.3.5 |
| Avalanching | $SGR_a$, $SGR_C$ | 0.17245, 250 m | Coefficients α (snow holding depth dependant on the slope angle) and C (exponential regression function) within the | Parameters as calibrated by Ragettli et al. (2015). |

| | | | | |
|---|---|---|---|---|
| | | | SnowSlide method of Bernhardt and Schulz (2010): $S_{max} = C\ exp(-\alpha\beta_{SP})$, where $S_{max}$ is the maximum storage capacity of the snowpack (m) and $\beta_{SP}$ is the slope given by the absolute elevation difference between a grid cell and the one downstream. | |
| Monthly crop coefficients | $K_c$ per month and land cover | See Table 3. | Crop coefficients. | See Section 1.3.7 |
| Soil properties | Ks, Θr, Θs, L, Exph, Expv | Derived per soil layer and class. | Hydraulic conductivity at saturation, water content ratio residual at 1500 kPa, water content ratio residual at saturation, slope of the logarithmic tension-moisture curve, Brooks-Corey exponent for the permeability-saturation curve (horizontal), Clapp-Hornberger exponent for the percolation equation (Expv) | See Section 1.3.8 |
| Groundwater | $K_{sH}$, $K_{sV}$, $\Theta_E$ | 0.0000003, 0.000003, 0.54 | Hydraulic conductivity (horizontal), hydraulic conductivity (vertical), water content ratio residual | As previously applied in TOPKAPI-ETH. |

### 1.3.1 Precipitation partition

Precipitation is partitioned into solid (snow) and liquid (rain) using a single temperature threshold in TOPKAPI-ETH. In the absence of precipitation type measurements in the catchment we apply the Ding et al. (2014) method which considers the elevation, relative humidity and wet-bulb temperature in the precipitation partition at all the WRF input grid cells. Specifically the input data used were the bias-corrected precipitation, bias-corrected air temperature and non-bias corrected vapour pressure (calculated from the non-bias corrected air temperature and relative humidity data) at each of the grid cells, from which the necessary variables were calculated to apply the Ding et al. (2014) approach. The sum of liquid and solid precipitation (sleet is apportioned to both) for a range of single temperature threshold partitions was compared against that derived from the Ding et al. (2014) method. The temperature threshold chosen was that which minimises the difference with the Ding et al. (2014) method. Due to the range in climate and elevation characteristics across all of the WRF points in the catchment there is a range in the calibrated threshold temperature (between 1.2°C and 4.9°C), with the threshold temperature increasing with elevation and decreasing with vapour pressure and air temperature (Figure 5). Since the model can only include a single temperature threshold we apply the median value of 2.2°C within the model.

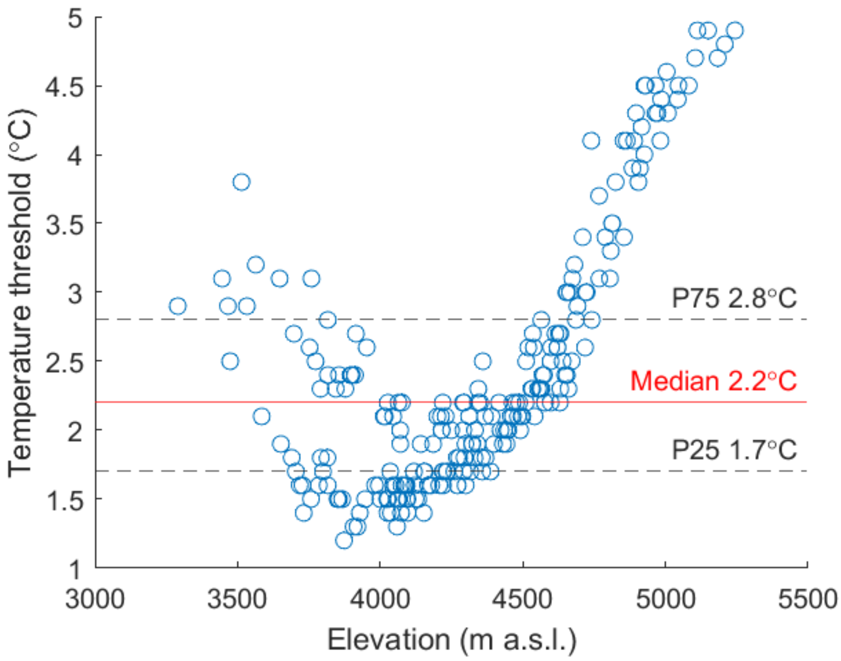


*Supplementary Figure 5 Calibrated precipitation phase temperature threshold for all WRF grid cells, against the elevation. Horizontal lines show the 75th percentile, median and 25th percentile of the calibrated temperature thresholds.*

### 1.3.2 Melt parameters

Glacier ice and snow melt is calculated in TOPKAPI-ETH using an Enhanced Temperature Index (ETI) approach given in equation 1 (Pellicciotti et al., 2005).

$$M = \begin{cases} TF\,T + SRF(1-\alpha)G & T > \mathrm{TT} \\ 0 & T \leq \mathrm{TT} \end{cases}, \quad (1)$$

Where M is the melt rate (mm h$^{-1}$), T is near-surface air temperature (°C), G is incoming shortwave radiation (W m$^{-2}$), α is albedo, TF is the temperature factor (mm h$^{-1}$ °C), SRF the shortwave radiation factor (m$^{2}$ mm W$^{-1}$ h$^{-1}$ °C) and TT the threshold air temperature (°C) above which melt occurs. The three parameters in this equation (TF, SRF and TT) require calibration. To calibrate these for Peruvian glaciers, a full energy balance melt model, Tethys-Chloris (T&C) was run at five on-glacier weather stations located across Peru, three in the Rio Santa catchment (Artesonraju, Shallap and Cuchillacocha glaciers) and two in the Cordillera Vilcanota, near Cusco (Quisoquipina Glacier and Quelccaya Ice Cap). The melt outputs of these runs were validated against a combination of measured ablation stake data, surface height change and albedo measurements. Full details of the data used, model equations and validation of the outputs is explained in Fyffe et al. (2021).

To find the optimum ETI parameters the ETI model was run using 1000 sets of randomly chosen parameters (within reasonable ranges), with the ETI melt evaluated against the T&C melt using an objective function (following Ayala et al. 2017). In this case the objective function was a combination of the absolute percentage bias (scaled to be within 0 and 1) and the Nash Sutcliffe efficiency criterion. This combined index was used so that the magnitude of melt matched well, along with the form of the diurnal melt cycle. The best parameter sets for the full record at each site were then cross-validated by applying these parameters to calculate the ETI melt at all other sites. The highest combined index across four of the five sites (excluding Quelccaya Ice Cap) was found at Artesonraju Glacier, where the best parameters were found to be SRF = 0.0067 m$^{2}$ mm W$^{-1}$ h$^{-1}$ °C, TF = 0.0181 mm h$^{-1}$ °C and

TT = 1.2547°C. We therefore applied the Artesonraju ETI parameters within the TOPKAPI-ETH model. The percentage bias in melt derived from the ETI model using the Artesonraju Glacier parameters ranged from 5.5% to 3.7% across the four sites. The ETI melt model did not work well on Quelccaya Ice Cap since the station here is at very high elevation where mass loss is dominated by sublimation, with very little melt.

#### 1.3.3 Albedo parameters

In TOPKAPI-ETH the temporal evolution of the snow albedo is parameterised by the deep snow equation derived by Brock et al. (2000), given in equation 2.

$$\alpha = B_a - B_b log_{10} PT \quad (2)$$

Where $B_a$ and $B_b$ are parameters which require calibration. An additional parameter, the threshold precipitation (PT) (mm $h^{-1}$) used to refresh the albedo back to that of new snow, is also required. The albedo of the surface can be calculated from outgoing shortwave radiation divided by incoming shortwave radiation measured over the glacier surface, with the accumulated daily albedo calculated for each day (following van den Broeke et al. (2004)) for comparison with the albedo model. These measurements were available at Artesonraju and Shallap Glaciers. The three parameters were calibrated by calculating modelled albedo based on 1000 parameter sets and comparing the modelled albedo to that measured. Due to the discontinuous snow cover during the wet season, periods of continuous snow cover were identified and the calibration was applied for each period individually, using the Nash Sutcliffe efficiency criterion as the objective function. The best parameter sets were tested against each site and period. It was found that the parameter sets of Artesonraju and Shallap were similar and showed some transferability between sites. The best parameter set was chosen to be that for the longest suitable period at Artesonraju Glacier, where $B_a$ = 0.75, $B_b$ = 0.115 and PT = 1 mm $h^{-1}$, with a Nash-Sutcliffe value of 0.495.

#### 1.3.4 Glacier reservoir parameters

Runoff from over glaciers (including melt and rain) in TOPAKPI-ETH is routed using two linear reservoirs, for the ice and snow-covered areas, using equation 3.

$$Q^t = Q^{t-\Delta t} exp\left(-\frac{\Delta t}{k}\right) + \left(1 - exp\left(-\frac{\Delta t}{k}\right)\right) Q_{in}^{\Delta t} \quad (3)$$

Where Q is discharge, t is the timestep, k is the retention factor which varies for the ice and snow melt reservoirs and $Q_{in}^{\Delta t}$ is the inflow of water into the reservoir during the current time step. The Casa de Aqua measured discharge was used as the calibration dataset since its catchment has a high glacier area (22%) and limited impact from upstream reservoirs. We did not use the discharge from Cuchillacocha Glacier, despite its higher proportional glacier area, since field observations suggested that wind acting on the lake upstream of the gauging station may influence the diurnal water level measured. The retention factors ($k_s$ and $k_g$) are calibrated by iterating through a range of values, with the objective to maximise 1 - RMSEf, where RMSEf is the root mean squared error between the average hourly discharge of the model and measurements divided by the average of the measured average hourly discharge. This method then focuses on finding the correct lag time between the melt and runoff peaks, which is the purpose of the linear reservoirs.

#### 1.3.5 Glacier evolution parameters

Glacier evolution is parameterised within TOPKAPI-ETH using the Δh-parameterisation of Huss et al. (2010). At the end of each glaciological year (31st of October) the mass change over each glacier is redistributed following a prescribed Δh-parameterisation (which relates normalised ice thickness change to the normalised elevation). This allows the glacier volume

and area in the model to be adjusted to take account of ice flow. To derive a Δh-parameterisation suitable for the Rio Santa catchment, glacier elevation change data from Hugonnet et al. (2021) from between 2000 and 2020 were used. The elevation change values (Δh) were averaged in 50 m elevation bins for each glacier in the catchment, and then the elevation change profile was smoothed by applying a running average of sets of three elevation bands. The elevation change and the elevation of each band was then normalised for each glacier following Huss et al. (2010). The dataset of all the glacier profiles was filtered to remove those where the minimum change in elevation occurred at a normalised elevation >0.6 (on the lower portion of the glacier), since this indicates an unrealistic elevation change profile. The median normalised Δh for the filtered set of glaciers was then calculated for bins of normalised elevation, with a relationship then fitted to these values following the same form as the Huss et al. (2010) parameterisations:

$$\Delta h = (h_r + a)^d + b(h_r + a) + c, \qquad (4)$$

where Δh is the normalised surface elevation change, $h_r$ is the normalised elevation range, and a to d are the coefficients which were calibrated. The fitted relationship quite closely matches the form of the small glacier Δh-parameterisation given in Huss et al. (2010), except that at higher elevations there is greater mass loss (Figure 6).

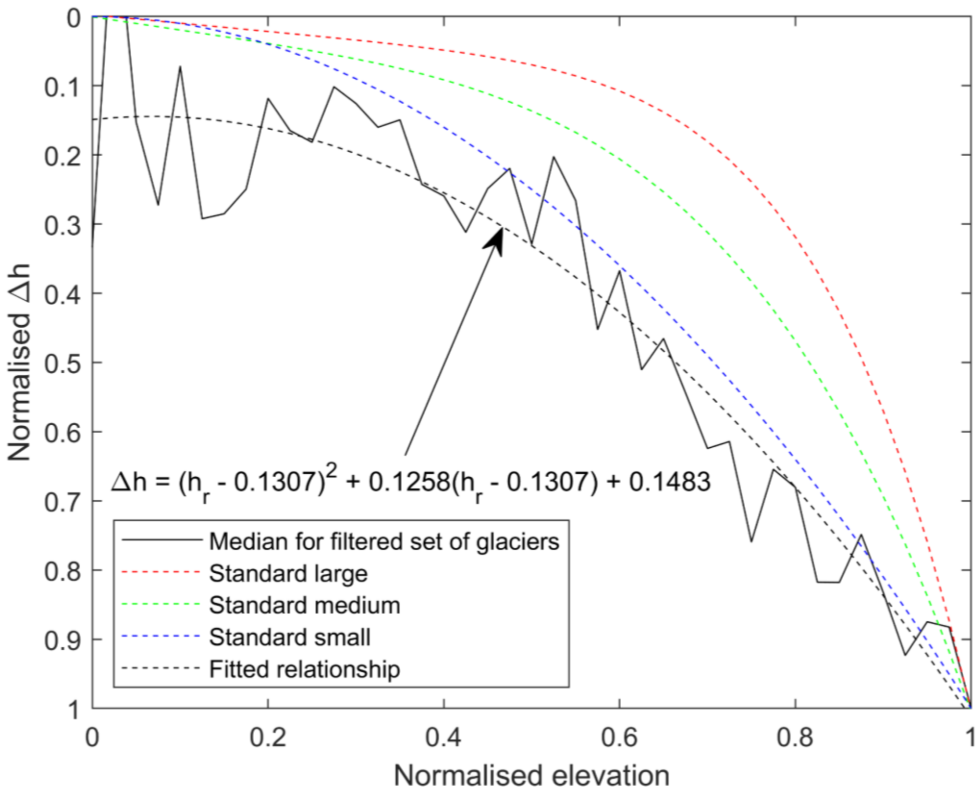


*Supplementary Figure 6 Calibrated Δh-parameterisation for the Rio Santa catchment, alongside those derived for glaciers of different sizes by Huss et al. (2010).*

### 1.3.6 Air temperature over glaciers

Air temperature is decreased over clean glaciers in TOPKAPI-ETH, due to the general cooling effect of glaciers on near surface air temperature (Greuell and Bohm, 1998; Carturan et al., 2015). Usually this value (Tmod) is found from comparison of on and off-glacier air temperature measurements adjusted to the same elevation (Ragettli et al., 2014; Ayala et al., 2016). However, Tmod calculated using stations at Artesonraju and Shallup glaciers

differed depending upon the site and off-glacier station applied, likely due to differences in the local glacier winds and the locations of the off-glacier stations (Shaw et al., 2017). We therefore calibrated the appropriate Tmod value for each sub-catchment by comparing the overall mass balance per sub-catchment with that given by Hugonnet et al. (2021) between 2010 and 2020 (compared with modelled glacier mass balances between 2010 and 2018). Sub-catchment mass balances were calculated as the average mass balance over all the glaciated cells in the sub-catchment where there were both mass balance data from Hugonnet et al. (2021) (used for calibration) and from the altitudinally resolved mass balances (used for evaluation), which excluded glaciers <2 km$^2$. We did this step after the other glacier melt and albedo parameters had already been calibrated. This was achieved by running the model with three differing Tmod values, which allowed the relationship between Tmod and glacier mass balance to be found, and consequently the Tmod value at which the sub-catchment glacier mass balance difference was zero. We allowed Tmod to vary in a range between 0.25°C and 3.46°C, as these are the range of measured values found across six South American glaciers by Shaw et al. (unpublished analysis). The calibrated Tmod values were found to be within this range for 10 of the 18 glaciated sub-catchments. For three sub-catchments the calibrated value was below the lower threshold (401, 408 and 409), and for two sub-catchments the calibrated value was above the upper threshold (415, 425). We applied the lower and upper limit of the Tmod value in these catchments, respectively. The remaining three sub-catchments had very small glaciated areas resulting in no or very limited remote sensing mass balance data (glaciers <2km$^2$ area, sub-catchments, 410, 412 and 416), for those we prescribed a Tmod value of 1°C (Ayala et al., 2016).

Air temperatures are usually warmer over debris-covered glaciers compared to the surrounding environment (Brock et al., 2010), however since we lacked on-glacier air temperature measurements above debris in the region we used a Tmod_debris value of 0.3°C as calibrated by Ayala et al. (2016).

### 1.3.7 Monthly crop coefficients

Evapotranspiration from each of the landcover types is controlled by monthly varying crop coefficients. These were derived from literature values, accounting for the Peruvian seasonality as much as possible as shown in Table 3 and justified in Table 4.

*Supplementary Table 3 Crop coefficients for each landcover type and month. For header descriptions see Table 4.*

| Month | Agri | Bo | Br-al | Br-me | Esv | Ma | Mi | PF | Pj | R | U | Lake |
|---|---|---|---|---|---|---|---|---|---|---|---|---|
| Jan | 1.05 | 1.10 | 0.91 | 0.91 | 0.21 | 0.90 | 0.21 | 0.66 | 1.06 | 1.05 | 1.00 | 1.05 |
| Feb | 0.95 | 1.10 | 0.91 | 0.91 | 0.21 | 0.96 | 0.21 | 0.69 | 1.13 | 1.05 | 1.00 | 1.05 |
| Mar | 0.65 | 1.10 | 0.91 | 0.91 | 0.21 | 1.12 | 0.21 | 0.76 | 1.30 | 1.05 | 1.00 | 1.05 |
| Apr | 0.65 | 1.10 | 0.91 | 0.91 | 0.21 | 0.70 | 0.21 | 0.56 | 0.85 | 1.05 | 1.00 | 1.05 |
| May | 0.52 | 1.10 | 0.72 | 0.72 | 0.21 | 0.37 | 0.21 | 0.41 | 0.51 | 1.05 | 1.00 | 1.05 |
| Jun | 0.52 | 1.10 | 0.72 | 0.72 | 0.21 | 0.21 | 0.21 | 0.33 | 0.35 | 1.05 | 1.00 | 1.05 |
| Jul | 0.26 | 1.10 | 0.72 | 0.72 | 0.21 | 0.20 | 0.21 | 0.33 | 0.33 | 1.05 | 1.00 | 1.05 |
| Aug | 0.23 | 1.10 | 0.72 | 0.72 | 0.21 | 0.20 | 0.21 | 0.33 | 0.33 | 1.05 | 1.00 | 1.05 |
| Sep | 0.33 | 1.10 | 0.72 | 0.72 | 0.21 | 0.33 | 0.21 | 0.39 | 0.47 | 1.05 | 1.00 | 1.05 |
| Oct | 0.54 | 1.10 | 0.72 | 0.72 | 0.21 | 0.60 | 0.21 | 0.52 | 0.75 | 1.05 | 1.00 | 1.05 |
| Nov | 0.92 | 1.10 | 0.91 | 0.91 | 0.21 | 0.59 | 0.21 | 0.51 | 0.74 | 1.05 | 1.00 | 1.05 |
| Dec | 0.84 | 1.10 | 0.91 | 0.91 | 0.21 | 0.79 | 0.21 | 0.61 | 0.95 | 1.05 | 1.00 | 1.05 |

*Supplementary Table 4 Justification for monthly crop coefficients.*

| Code | Description | Derivation of coefficients |
|---|---|---|
| Agri | Agriculture | Average of values for the main crops grown in the catchment (native and Yungay potatoes, corn and wheat). The timing of the crop stages was taken from descriptions in Gurgiser et al. (2016), with the native and Yungay potato crop coefficients from Sanabria and Lhomme (2013) and the FAO guidelines (Allen et al., 1998) for potato. Crop coefficients for corn were the FAO values for sweet corn and for wheat the FAO values for spring wheat. |
| Bo | Bofedales | FAO values for short vegetation in mid-season used since most wetlands in the Cordillera Blanca (57%) are cushion plant peatlands, followed by gramminoid wet meadows (32%) and cushion wet meadows (12%) (Chimner et al., 2019), rather than the reed swamps/cattails and bullrushes in the wetland classes in the FAO. |
| Br-al | High Andean relict forest | Applied values for evergreen broadleaved forests from Liu et al. (2017) for summer in the wet season (as values are higher then and in general crop coefficients increases with precipitation) and the value for winter in the dry season. |
| Br-me | Meso Andean relict forest | Applied values for evergreen broadleaved forests from Liu et al. (2017) for summer in the wet season (as values are higher then and in general crop coefficients increases with precipitation) and the value for winter in the dry season. |
| Esv | High Andean area with little to no vegetation | Value of 0.21 derived from analysis of crop coefficients for bare soil at high elevation (Pappas et al., 2016). |
| Ma | Shrubby thicket | Values were derived from the relationship between the open scrubland crop coefficient and precipitation given in Liu et al. (2017), based on the monthly average WRF precipitation for the Rio Santa. |
| Mi | Mining centre | Value of 0.21 derived from analysis of crop coefficients for bare soil at high elevation (Pappas et al., 2016). |
| PF | Plantation forest | Values were derived from the relationship between the evergreen needleleaf forest crop coefficient and precipitation given in Liu et al. (2017), based on the monthly average WRF precipitation for the Rio Santa. |
| Pj | Andean scrubland | Values were derived from the relationship between grassland (since the scrubland is a combination of grasses and small shrubs) and precipitation given in Liu et al. (2017), based on the monthly average WRF precipitation for the Rio Santa. |
| R | River | Using FAO value for open water <2 m depth or in subhumid climate or tropics. |
| U | Urban | Applied value of 1. |
| Lake | Lagoons and lakes | Using FAO value for open water <2 m depth or in subhumid climate or tropics. |

### 1.3.8 Soil parameters

TOPKAPI-ETH was run with two soil layers, the upper up to 0.3 m depth, and the lower the remainder of the soil depth (up to 2 m). Ten soil classes were derived based on regions with similar hydraulic conductivities (see Table 1). For each of these regions and for each soil layer the mean sand, clay and soil organic carbon fractions from the SoilGrids 250 m v2.0 dataset (Poggio et al., 2021) was calculated. The soil hydraulic properties (specifically the horizontal and vertical hydraulic conductivity at saturation (Ks), the water content ratio residual at 1500 kPa ($\Theta_r$), the water content ratio residual at saturation ($\Theta_s$) and the slope of the logarithmic tension-moisture curve (L)) were calculated following soil water characteristic equations (e.g. Saxton and Rawls, 2006). The Brooks-Corey exponent for the permeability-

saturation curve (horizontal) (Exph) was approximated as 3+(2/L), while the Clapp-Hornberger exponent for the percolation equation (Expv) was given a value of 2.75.

### 1.3.9 Treatment of reservoirs and lakes

TOPKAPI-ETH includes a representation of reservoirs which we used to attenuate runoff in large reservoirs and lakes. Lake volumes (for lakes > 0.1 $km^2$) were available from the HydroLAKES database (Messager et al., 2016), with the relationship between lake volume and area for lakes <0.4 $km^2$ within the study catchment used to derive the volume for all lakes. Based on photographs and measurements of the weir downstream of Llaca Glacier lake we derived a level/discharge relationship based on the fully contracted rectangular weir equation of Francis (1883, cited by Bureau of Reclamation, 2001). The weir length applied in this equation was scaled from that measured at Llaca Glacier to the other lakes based on an estimate of the maximum hourly discharge for each lake. At the study catchment outlet both the maximum hourly discharge and mean monthly discharge was known, and this was used to calculate a multiplier that could be applied to the average monthly discharge available for all the lake outlet locations. This allowed the calculation of discharge values based on the weir equation for a given level for all the lakes. TOPKAPI-ETH also requires level/volume relationships and these were based on a linear scaling of the lake volume based on the change in level and related to the lake area. Once the level is above a set maximum additional runoff is presumed to flow over the spillway and not be controlled by a weir so it is then not controlled by the reservoir equations. Target reservoir levels are maintained in TOPKAPI-ETH, and we set those to be a constant minimum level. In total there were 192 lakes in the study catchment but we apply these reservoir equations to only those with an area >0.04 $km^2$ and with a mean monthly discharge >0.1 $m^3s^{-1}$ (28 in total). These thresholds were designed to remove the smallest lakes which do not form part of the TOPKAPI-ETH stream network (due to the grid resolution) and those with very small discharges where flow attenuation would have a minimal influence on downstream flows. Due to numerical problems (reservoir levels continually decreasing in an unrealistic way) we also switched off 6 lakes so that 22 were active in the final model.

## 1.4 Model confirmation datasets

### 1.4.1 Snow cover

Modelled snow cover was evaluated using the daily MODIS remote sensing product MOD10A1 V6.1 (Hall and Riggs, 2021), which has a spatial resolution of 500 m. Snow cover was identified in the images using a 'standard' NDSI threshold of 0.4, while uncertainties were estimated using values of 0.1 and 0.45 (Härer et al. 2018; Zhang et al., 2019), considering that the ideal NDSI threshold for a region depends on local factors such as the prevalence of vegetation. Low-quality or cloud-covered pixels were removed from all the images, and images were discarded if more than 80% of the catchment was cloud covered, or if less than 80% of pixels in the catchment had a quality assessment of 'good' or 'best'. Glacierised areas were excluded when comparing the MODIS data to model results, on the basis that MODIS struggles to distinguish between ice and snow because they tend to have similar NDSI values. Snowlines were determined by first calculating the fraction of the catchment in each image that was snow covered, then assuming that the snow preferentially covers higher rather than lower elevations (following Meier (1975)). We also tested using the method of Krajčí et al. (2014) to derive the snowline elevations, but the application of the Ps_min threshold (representing the proportion of the catchment which is snow-covered), removed the majority of the imagery. When averaging the snowline elevations for comparison modelled data were not included in the average if MODIS data were not available for that day. A modelled cell was defined as snow-covered if the snow water equivalent was > 10 mm w.e, with uncertainties estimated by varying this threshold

conservatively from >1 mm w.e. to > 20 mm w.e. This range is based on analysis by Parajka and Blöschl (2008) who found that the accuracy of snow cover mapping by MODIS was not sensitive to snow depth thresholds between 1 and 3 cm (which we equate to ≅ 6 to 18 mm w.e., applying a snow density of 0.6 g $cm^{-3}$, derived from the mean value across 24 snow pit measurements on Artesonraju Glacier (unpublished data)).

Snow cover was also compared against station measurements of albedo at two on-glacier sites (Shallap Glacier and Artesonraju Glacier) and one off-glacier site (Shallap Moraine New). Details of these stations and their locations can be found in Fyffe et al. (2021). The measured albedo was calculated as the accumulated albedo following van den Broeke et al. (2004). The surface was classified as snow-covered when the albedo at the on-glacier sites was >0.4. At the off-glacier site (Shallap Moraine) the surface was considered snow-covered if the albedo was greater than 1 standard deviation above the running median albedo over the surrounding week. The median albedo was calculated excluding values >0.2, to ensure no snow-covered periods were included.

### 1.4.2 Altitudinally-resolved glacier mass balance

We estimated the decadal-average altitudinal mass balance of glaciers in the Rio Santa Basin in the Cordillera Blanca following the Monte-Carlo mass-continuity method of Miles et al. (2021) applied to the results of Dussaillant et al. (2019) for the period 2015-2019. This method requires ice thicknesses, which were those derived by Farinotti et al. (2019), and ice velocities, which were those derived by Millan et al. (2022). We then used the continuity equation to model flux divergence with the ice thickness and velocity datasets, and to estimate volume-mass conversion factors. This followed the methods of Miles et al. (2021) but with the addition of a variable ice thickness-dependent distance for determination of the flux divergence (similar to Van Tricht et al., 2021), enabling fully-distributed estimates of flux divergence.

### 1.4.3 Stream discharge

*Supplementary Table 5 Details of gauging stations used to validate the modelled runoff. Runoff data originates from Mateo et al. (2022). Note there are gaps in the time series between the start and end period for some stations. We did not use the Cuchillacocha Glacier record for comparisons due to some uncertainties in the level measurements. Glacier areas are relevant to the areas used for modelling.*

| Name | Lat | Long | Elevation (m a.sl.) | Start | End | Catchment area ($km^2$) | Glacier area ($km^2$) | % glacier cover |
|---|---|---|---|---|---|---|---|---|
| Cuchillacocha | -9.41425 | -77.3542 | 4631 | 06/07/2008 14:15 | 18/02/2019 12:14 | 4.16 | 2.18 | 52.4 |
| Casa de Agua | -9.46469 | -77.3787 | 3948 | 11/07/2009 14:00 | 17/06/2019 09:00 | 67.21 | 14.89 | 22.2 |
| Pumapampa | -9.88167 | -77.2474 | 4287 | 10/07/2008 15:15 | 01/07/2016 14:45 | 57.81 | 4.12 | 7.1 |
| Pachacoto | -9.85241 | -77.4023 | 3738 | 03/07/2008 12:45 | 06/12/2018 14:30 | 207.72 | 8.27 | 4.0 |
| Llanganuco | -9.07859 | -77.6511 | 3850 | 06/11/2008 11:00 | 22/01/2019 16:30 | 87.14 | 23.61 | 27.1 |
| Querococha | -9.72639 | -77.3333 | 4005 | 30/06/2008 14:30 | 27/12/2018 09:30 | 63.95 | 0.68 | 1.1 |

## 1.5 Model sensitivity

To quantify the uncertainty of the TOPKAPI-ETH model outputs due to parameter selection we assess the model sensitivity to the calibrated snow and ice parameters by re-running the model while changing the value of one parameter at a time (Shaw et al., 2020) to either a likely maximum or minimum. The parameter ranges were taken as far as possible from the

range of values identified during the calibration process (Table 6), hence why we identify them as ‘likely’, rather than theoretical, ranges. For computational efficiency we ran the model for one hydrological year (2009) following the spin-up period and we did not conduct a full Monte-Carlo analysis of parameter uncertainty. For the sensitivity analysis we applied the air temperature reduction over glaciers uniformly over all catchments. Due to the high sensitivity of the modelled snow melt on the PrecSF parameter (the threshold temperature between snow and rain) we also conducted full model runs with the minimum and maximum calibrated values. This allowed us to assess the impact on the modelled quantity and fraction of snow and ice melt to total inputs, as well as the impact on the modelled snowlines used for validation.

*Supplementary Table 6 Range of model parameters used to assess model sensitivity. For a description of the parameters see Table 2. Note we did not modify the lag parameters of the DETI model (Carenzo et al., 2016) since the melt amounts are primarily controlled by the sub-debris temperature and shortwave radiation factors (TFd1/2 and SRFd1/2).*

| Parameter | Current | Min | Max | Notes |
|---|---|---|---|---|
| $B_a$ | 0.75 | 0.64 | 0.85 | Range across all snow-covered periods calibrated for Shallap, Artesonraju and Quisoquipina |
| $B_b$ | 0.115 | 0.1 | 0.17 | Range across all snow-covered periods calibrated for Shallap, Artesonraju and Quisoquipina |
| PT | 1 | 0.4 | 2.9 | Range across all snow-covered periods calibrated for Shallap, Artesonraju and Quisoquipina |
| $\alpha_{debris}$ | 0.18 | 0.126 | 0.234 | 30% difference |
| $\alpha_{glacier}$ | 0.3007 | 0.2314 | 0.3186 | Range of mean measured ice albedo values between Artesonraju, Shallap, Cuchillacocha and Quisoquipina. |
| SRF | 0.0067 | 0.0044 | 0.0067 | Range of the calibrated values for the full time series of Artesonraju, Shallap, Cuchillacocha and Quisoquipina. |
| TF | 0.0181 | 0.0181 | 0.1824 | Range of the calibrated values for the full time series of Artesonraju, Shallap, Cuchillacocha and Quisoquipina. |
| PrecSF | 2.2 | 1.2 | 4.9 | Range across WRF points in the catchment calibrated against Ding et al. (2014) method. |
| TT | 1.2547 | -0.1445 | 1.6103 | Range of the calibrated values for the full time series of Artesonraju, Shallap, Cuchillacocha and Quisoquipina. |
| Tmod | 1 | 0.25 | 3.46 | Range of measured values found across six South American glaciers (unpublished analysis). |
| Tmod Debris | 0.3 | 0 | 2 | Shaw et al. (2020) |
| TFd1 | 0.017 | 0.0119 | 0.0221 | Shaw et al. (2020) |
| TFd2 | 0.4043 | 0.283 | 0.5255 | Shaw et al. (2020) |
| SRFd1 | 0.0114 | 0.0079 | 0.0148 | Shaw et al. (2020) |
| SRFd2 | 44.2 | 30.95 | 57.45 | Shaw et al. (2020) |
| $SGR_a$ | 0.17245 | 0.12 | 0.224 | Shaw et al. (2020) |
| $SGR_C$ | 250 | 175 | 325 | Shaw et al. (2020) |
| $k_g$ | 33.86 | 1 | 2000 | Range used in the calibration process. |
| $k_s$ | 1184.2 | 1 | 2000 | Range used in the calibration process. |

## 1.6 Deriving snow affected area across Peru

To understand the areas where ephemeral snow may be important across Peru we found the relationship between wet season mean air temperature and modelled wet season

percentage snow cover. The percentage snow cover in this regard is the percentage of hourly time steps with snow cover at that point location. These data are from a collection of modelled points where data were saved with an hourly temporal resolution at transects across the catchment, and on both the Blanca and Negra sides. The relationship was derived for points which had wet season snow cover percentages >=10% and <=90% to avoid points with little or continuous snow cover from influencing the gradient of the relationship (shown in Figure 5b of the main paper). This relationship was then applied to the average wet season non-bias corrected ERA5-Land Reanalysis 2 m air temperatures (Muñoz-Sabater, 2019) (where the average was applied over data from 2008-2018, matching the modelled period), to give the wet season percentage snow cover over all of Peru. We then classified this map of percentage snow covers into regions with 10-50% and 50-90% snow cover for visualisation purposes. We understand this method has limitations, especially as it does not take into account the precipitation patterns and differences in wet season length across the country.

We preferred to derive the snow-effected areas from the ERA5-Land air temperatures since in general we would expect ERA5-Land temperature patterns to be more robust than precipitation (e.g. Khadka et al., 2022). We also expect air temperature to be a good proxy for snow cover given its links to the precipitation phase and melt processes. We show in Figure 5 in the main paper the wet season percentage snow cover directly from ERA5-Land for comparison, which demonstrates a similar area coverage of snow cover compared to that derived from air temperature.

To identify the catchments which may be influenced by areas with partial snow cover we then selected all the HydroSHEDS level 7 catchments (Lehner et al., 2008) which intersected the snow-covered areas. We chose level seven catchments as the modelled upper Rio Santa catchment is the same scale. We then used the HydroSHEDS void-filled digital elevation model to calculate the hypsometry of all the selected catchments, as well as the modelled upper Rio Santa catchment (shown in Figure 5c of the main paper).

# 2 Supplementary Notes

## 2.1 Model performance

### 2.1.1 Glacier mass balances

To assess the ability of TOPKAPI-ETH to replicate the glacier mass balance we compare it against the altitudinally resolved glacier mass balances since they were not used in the calibration (Section 1.4.2). All glacier mass balance comparisons were conducted over glacier cells common to TOPKAPI-ETH (which uses the INAIGEM (2018) glacier outlines) and the remote sensing datasets (Randolph Glacier Inventory (RGI) version 6.0 outlines for both datasets (RGI Consortium, 2017)) and restricted to glaciers with surface area >2 km$^2$ for the altitudinally resolved mass balances. The comparison of the mass balance profile across the whole catchment is shown in Figure 1c in the main paper. We compare glacier mass balances at the catchment or sub-catchment scale since this increases the glacier area, reducing the uncertainty in the remote sensing data. The mean difference in mass balance across the sub-catchments with sufficient remote sensing data is -0.16 m w.e. a$^{-1}$ and the range is -1.36 m w.e. a$^{-1}$ to 0.81 m w.e. a$^{-1}$ (Figures 7 and 8a). The tendency for negative bias (model underestimation) is expected since the calibration of Tmod was performed over the model years 2010-2018, whereas the validation period is in the latter half (model years 2015-2018). We also show the comparison of the modelled sub-catchment mass balances against the Hugonnet et al. (2021) data (Figure 8b to d), noting that the 2010-2020 data were used in model calibration. At the glacier scale modelled ablation closely matches the stake and remote sensing data at all three studied glaciers (Figure 9),

however in the accumulation zone the model matches the remote sensing data well but underestimates accumulation compared to the snow pit measurements at Shallap and Gueshgue glaciers.

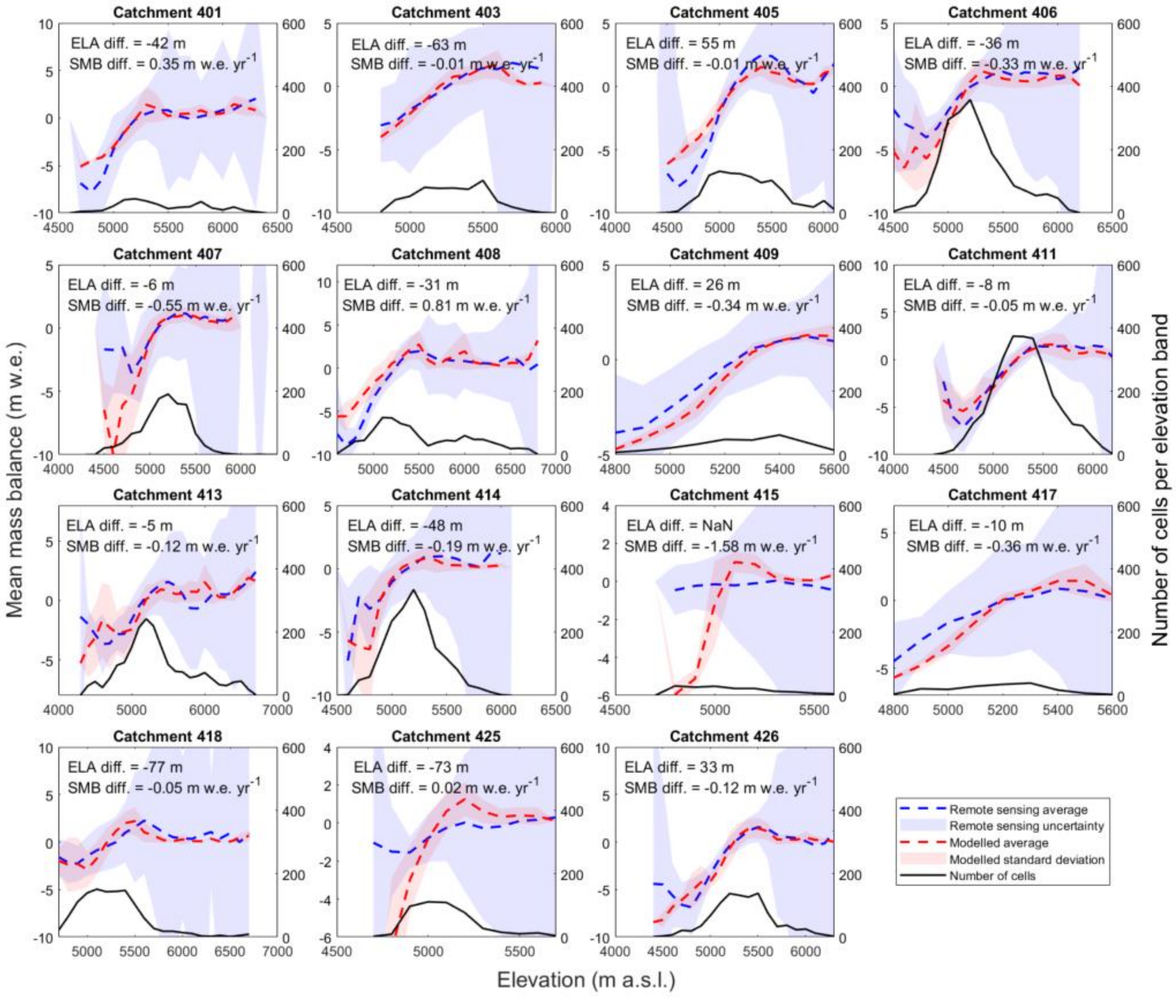


*Supplementary Figure 7 Comparison of modelled (2015-2018) and remote sensing (2015-2019) derived altitudinal glacier mass balance profile for each glaciated sub-catchment within the upper Rio Santa. Note we do not show the glaciated sub-catchments 410, 412 and 416 since there were either no or very few (<=17) overlapping glacier cells between the model outputs and remote sensing dataset. The dashed lines are the average mass balance for all the cells in each 100 m elevation band. The shading for the model results represents +/- the standard deviation of the mass balance per elevation band, whereas the shading for the remote sensing data represents the uncertainty of the average. The right hand axis shows the number of glaciated cells per elevation band which were used for the comparisons. The ELA was not calculated for catchment 415 since there were too few cells for a robust calculation.*

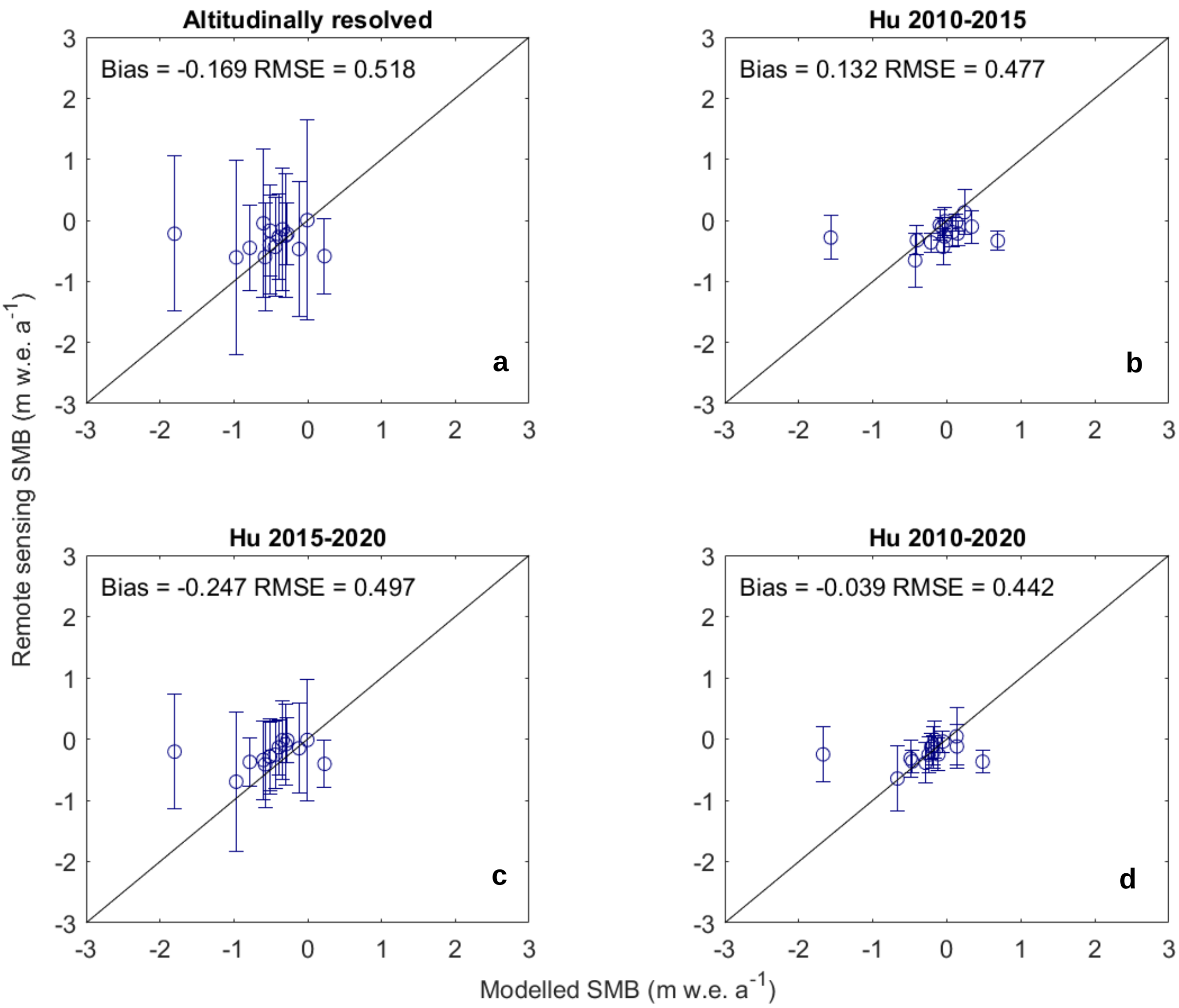


*Supplementary Figure 8 Comparison of sub-catchment average glacier mass balances (calculated as a simple average of all the compared cells) between the model and remote sensing dataset. Note we do not show the glaciated sub-catchments 410, 412 and 416 since there were either no or very few (<=17) overlapping glacier cells between the model outputs and remote sensing dataset. Error bars represent the uncertainty of the remote sensing dataset. The altitudinally resolved mass balances were those derived following the method of Miles et al. (2021) and are for the period 2015-2019 (model years 2015-2018), those labelled 'Hu' are from Hugonnet et al. (2021) for the years 2010-2015 (same model years), 2015-2020 (model years 2015-2018) and 2010-2020 (model years 2010-2018). Note that the Hu 2010-2020 data were used for calibration.*

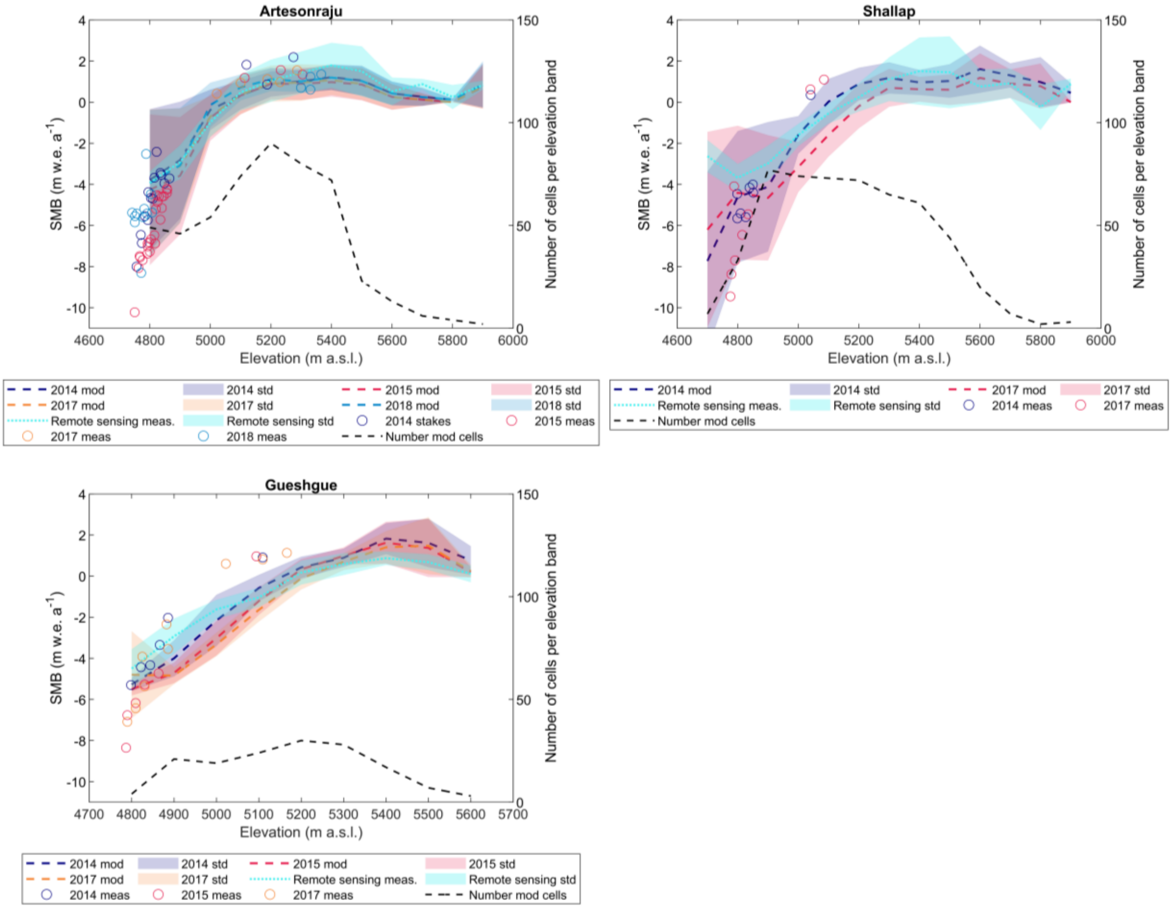


*Supplementary Figure 9 Comparison of modelled and measured glacier mass balances for three glaciers (Artesonraju, Shallap and Gueshgue). Here the circles are data from ablation stakes and snow pits and the turquoise line and shading is from the remote sensing derived mass balances, as explained in Section 1.4.2. We show the model results for the same years as the stake and snow pit data. We estimate the error associated with the ablation stake data to be +/- 5 cm (Reid et al., 2012), but these errors are not shown as they are very small relative to the y-axis scale.*

### 2.1.2 River discharge

Discharge data were only used to calibrate the glacier reservoir parameters, so runoff records provide an independent assessment of model skill. Comparisons of modelled and measured discharge (Figure 10) show good Nash-Sutcliffe (NS) and Kling-Gupta Efficiency (KGE) values across three of the five sub-catchments: Pumapampa, Llanganuco and Querococha (NS 0.48-0.69, KGE 0.68-0.73 for monthly runoff). At Pachacoto the NS is good (0.73) but the KGE is relatively low (0.38) as a result of model overestimation of wet season discharges, which is explained by the diversion of flows for irrigation into a neighbouring catchment and does not indicate model deficiency. At Casa de Aqua the NS values are low (-0.11), despite reasonable KGE values (0.47), with overall flow magnitudes well replicated but the seasonal variations not always well modelled. In catchments without large reservoirs the modelled runoff is more flashy than measured, likely because the model cannot replicate some local water storage mechanisms (e.g. smaller lakes and complex surface-groundwater interactions).

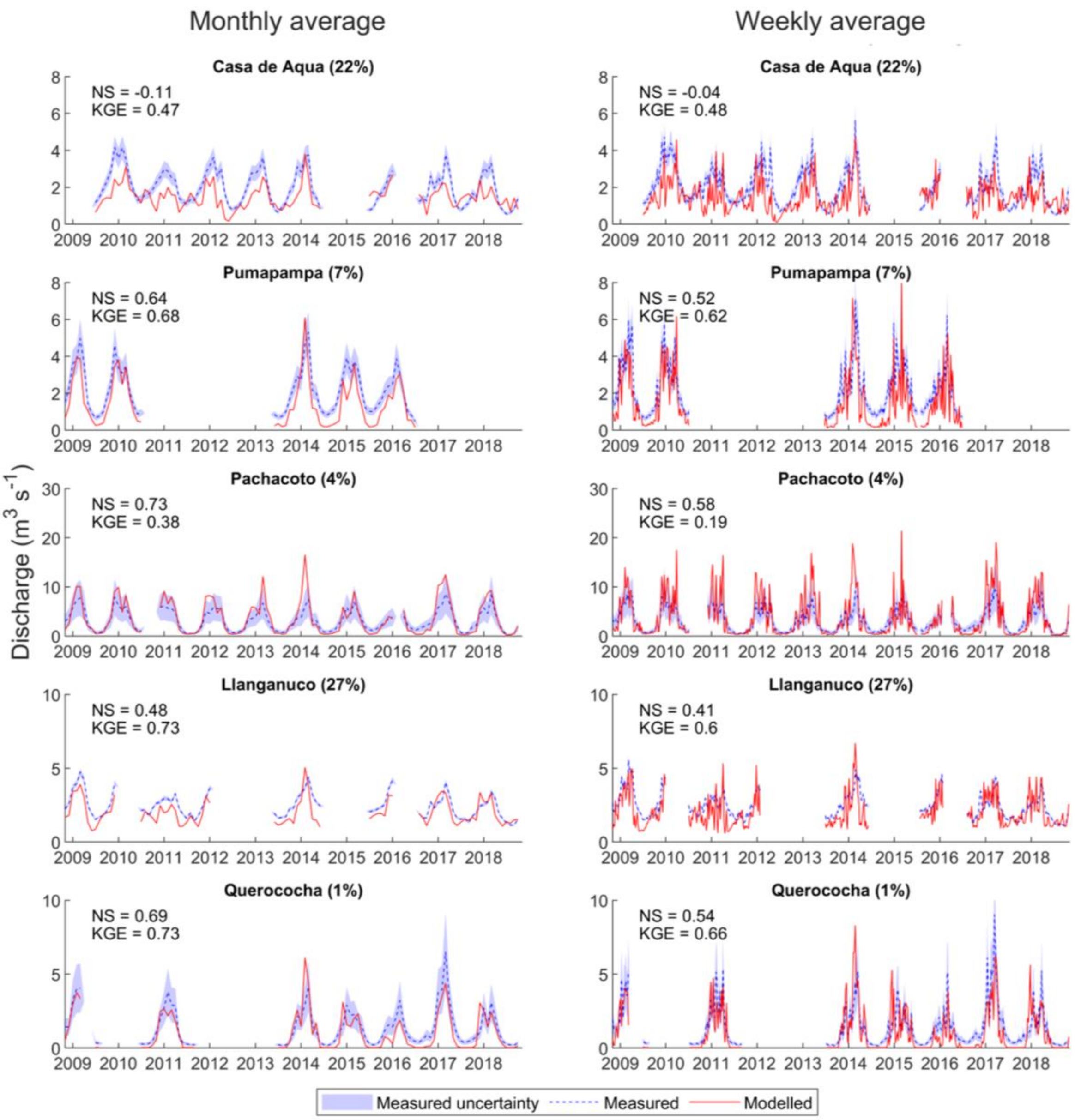


*Supplementary Figure 10 Comparison of modelled and measured discharge from stations within the upper Rio Santa, both monthly averaged (left panels) and weekly averaged (right panels). NS is the Nash-Sutcliffe efficiency criterion and KGE is the Kling Gupta Efficiency criteria. The uncertainty of the measurements are calculated from the mean absolute percentage error of the measured gauged discharges against the rating curve for each gauging station. The percentage values in the titles are the percent glacier area of the catchments.*

### 2.1.3 Snow cover

Monthly snowline elevations from TOPAKPI-ETH compare well with those derived from MODIS (Figure 1d in the main paper), with a small bias of 24.3 m and an RMSE of 140 m. We also conducted a sensitivity test to determine the impact of varying the PrecSF parameter between the calibrated ranges of 1.2˚C and 4.9˚C (see Section 1.3.1) (Figure 11). This shows that the snowline elevation is relatively insensitive to this threshold temperature in the dry season, as the snowline evolution is controlled by melt processes, but in the wet season, the higher threshold temperature gives lower modelled snowline elevations. The applied PrecSF of 2.2°C gives a good balance of low bias and RMSE between the values of the other thresholds, giving confidence that this threshold is suitable for the catchment.

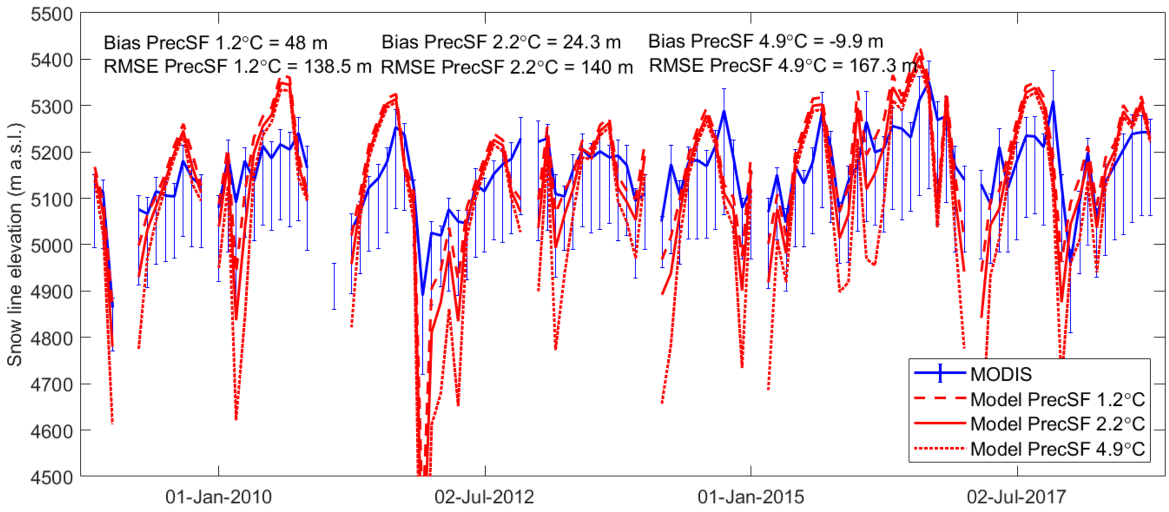


*Supplementary Figure 11 Comparison of monthly averaged snowline elevations between MODIS and the model, calculated using an approach following Meier (1975), and with the different modelled estimates a result of changing the PrecSF threshold. For all modelled snowlines cells were defined as snow-covered when SWE is > 10 mm w.e,*

Comparison of modelled with measured snow cover at point locations shows snow cover overestimation at Artesonraju Glacier and Shallap Moraine (off-glacier), but little bias at Shallap Glacier (Figure 12 and Table 7). Snow cover patterns are well replicated at Shallap Glacier and Shallap Moraine, although there are some wet season periods at Artesonraju Glacier where modelled snow cover is more continuous than measured.

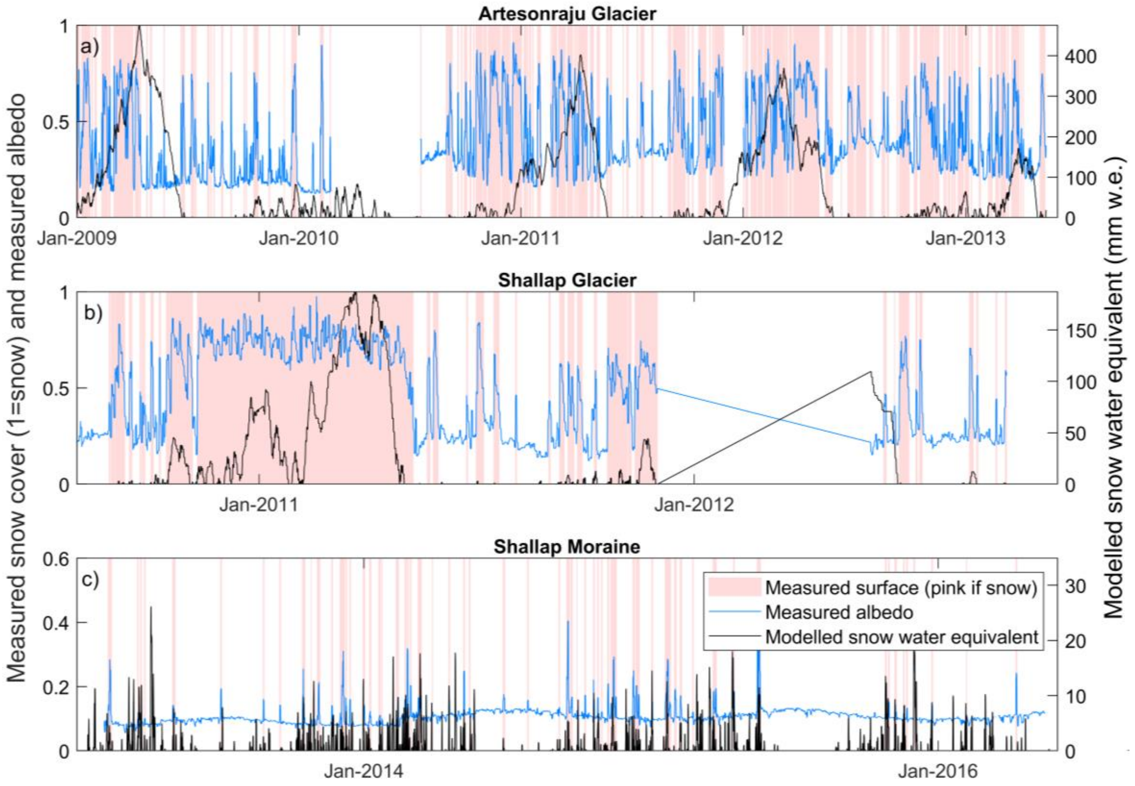


*Supplementary Figure 12 Comparison of modelled and measured snow cover at three point locations. Each panel represents snow cover as determined from albedo measurements at the point scale, with the measured albedo shown in blue and derived snow cover shown as pink bars. The modelled snow water equivalent is shown as a black line.*

*Supplementary Table 7 Statistics of the comparison of modelled and measured snow cover at three point locations. For details of the calculation of snow cover from the albedo measurements see Section 1.4.1.*

| Point | Correct ice/ground (%) | Correct snow (%) | Incorrect ice/ground (%) | Incorrect snow (%) |
|---|---|---|---|---|
| Artesonraju Glacier | 26.7 | 32.6 | 6.9 | 33.8 |
| Shallap Glacier | 40.2 | 38.3 | 12.8 | 8.8 |
| Shallap Moraine | 72.8 | 5.2 | 2.9 | 19.1 |

### 2.1.4 Model sensitivity

The sensitivity of the Rio Santa discharge and ice and snow melt amounts to the varied parameters (given in Table 6) is shown in Figure 13. The variations in most of the parameters do not change the discharge by more than 10%, with the only exceptions PrecSF (the threshold temperature between snow and rain), resulting in a 13% change between the maximum and minimum parameter values, TT (the threshold temperature to allow melt) with the change in discharge up to 20% and Tmod (the temperature decrease over clean glacier ice) with the change in discharge up to 23%.

As for discharge, snow melt is most sensitive to changes in PrecSF (112%), TT (15%) and Tmod (15%). The large impact of PrecSF is due to this parameter controlling how much snow is falling compared to rain, and due to its large influence, we conducted full model runs with the maximum and minimum calibrated parameters. Its impact on the amount and fractional contribution of melt and rain to catchment inputs is discussed in the main paper and shown in Figure 14, and its impact on the modelled snowlines used for validation is discussed in Section 2.1.3 and shown in Figure 11. Changes in snowmelt are also sensitive to changes in the albedo and melt parameters, but the differences are generally small (8.9% for $BP_a$ (albedo parameter), 6.4% for SRF (shortwave radiation factor) and 5.0% for TF (temperature factor). The avalanche parameter $SGR_a$ is also important, causing a 5.2% change in snow melt across the range of possible values.

Ice melt values are particularly sensitive to TT and Tmod, with values changing by 113% and 127%, respectively, across the range of values. Ice melt is also sensitive to PrecSF, but by much less than snow melt, with PrecSF variations resulting in a 21% change between the parameter range. Unsurprisingly, the other parameters which change ice melt by more than 10% are related to the calculation of albedo and melt: with $B_a$, $B_b$ and PT (used to calculate snow albedo, see equation 2) influencing the amount of ice melt by 63%, 12% and 13% respectively; and SRF and TF (the shortwave radiation and temperature factors used to calculate melt, see equation 1) influencing the amount of melt by 52% and 61%, respectively.

It is expected that changes in PrecSF would change the snowmelt since higher values would increase the air temperature at which precipitation falls as snow, and since the air temperature is high the snow would melt relatively quickly, increasing snow melt amounts. The upper PrecSF value used in the sensitivity experiments (4.9˚C) is relevant for the highest elevations in the catchment (see Figure 5), and so applying it to the entire catchment as here likely significantly overestimates snowfall. The lower threshold (1.2˚C) conversely will decrease the precipitation that falls as snow, reducing the snow melt amounts, although this value is relevant to the lowest elevations at which snowfall is expected. The median

value used for the main model runs (2.2˚C) is suitable for elevations close to the lower limit of snowfall (~4500 m a.s.l.) so should provide snowfall amounts which are correct at these elevations and slightly lower than expected (conservative) at higher elevations.

The sensitivity of snow and especially ice melt to the TT value is because this determines the temperature at which melt occurs. The standard run value is relatively high (1.3˚C) within the range (-0.14 to 1.6 ˚C). However, in reality, calibration of TT to a lower value would be accompanied by a reduction in the SRF and TF values, to give the same overall amount of melt, although here we change each parameter separately. The melt parameter calibration processes carried out to derive the standard values allowed all three parameters to vary (see Section 1.3.2).

The sensitivity of discharge and especially ice melt amounts to Tmod is because by controlling the air temperature over glacier areas it affects the amount and frequency of melt. In these sensitivity runs it was held constant over all glacier areas, but in the model set-up used in the paper this parameter was calibrated for each sub-catchment (see Section 1.3.6). Tmod essentially parameterises the influence of glacier winds to cool near-surface air temperatures over glaciers relative to the surrounding areas. Given the influence of this parameter on melt rates we would welcome further work to allow improved estimates of the magnitude of glacier cooling effects, and how they relate to the local climate, valley and glacier characteristics (e.g. Shaw et al., 2024).

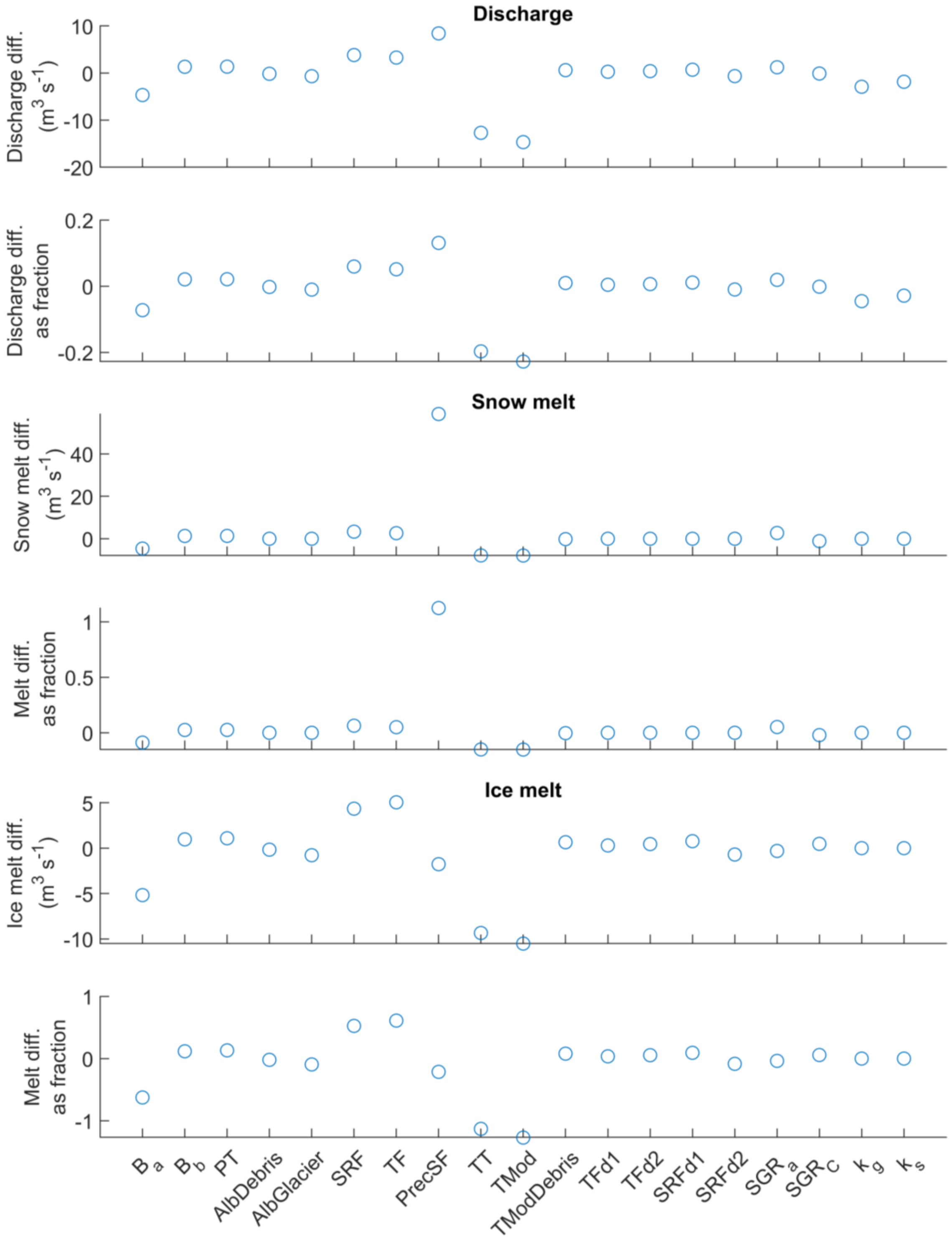


*Supplementary Figure 13 Change to the Rio Santa discharge and total catchment snow and ice melt due to variations in the parameters, as set out in Table 6. The differences are calculated as the value for the run with the maximum parameter value minus the value for the run using the minimum parameter value. The differences are also calculated as a fraction of the value for the standard run, which had a Tmod parameter value of 1˚C for all glacier cells.*

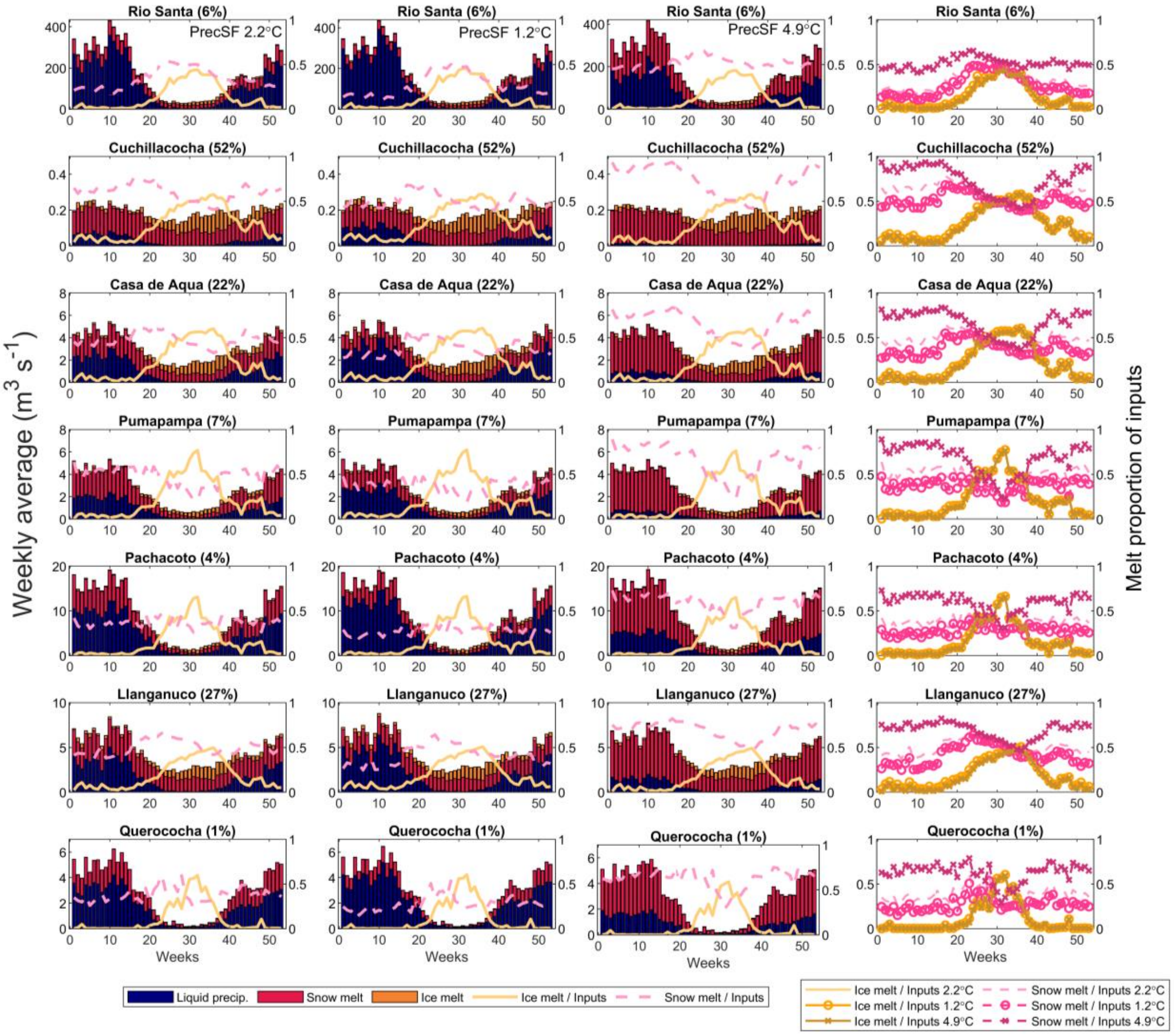


*Supplementary Figure 14 Sensitivity assessment of the amount and fractional contribution of liquid precipitation, snow melt and ice melt contributions to total inputs (composed of the sum of rain, snow melt and ice melt) into the catchment to a change in the temperature threshold between snow and rain (PrecSF). The first column contains the model results with the threshold as applied in the main paper (2.2°C), the second column is with a threshold of 1.2°C and the third column with a threshold on 4.9°C, the lowest and highest values represent the range of calibrated temperature thresholds across the catchment (see Section 1.3.1 for details of their calibration). The fourth column contains a comparison of the fractions of ice and snowmelt of total inputs for the three temperature thresholds.*

## 2.2 Snow dynamics

We present in this section additional figures to illustrate the snowfall and melt dynamics. Figure 15 shows the short-term variability in the snow water equivalent for a series of points at different elevations; Figure 16 illustrates the variations in snow water equivalent and duration of snow cover, through the analysis of snow-covered periods, Figure 17 illustrates the daily elevation of the snow line elevation, to give a seasonal perspective of snow cover change and Figure 18 shows the classification of snow cover in the catchment.

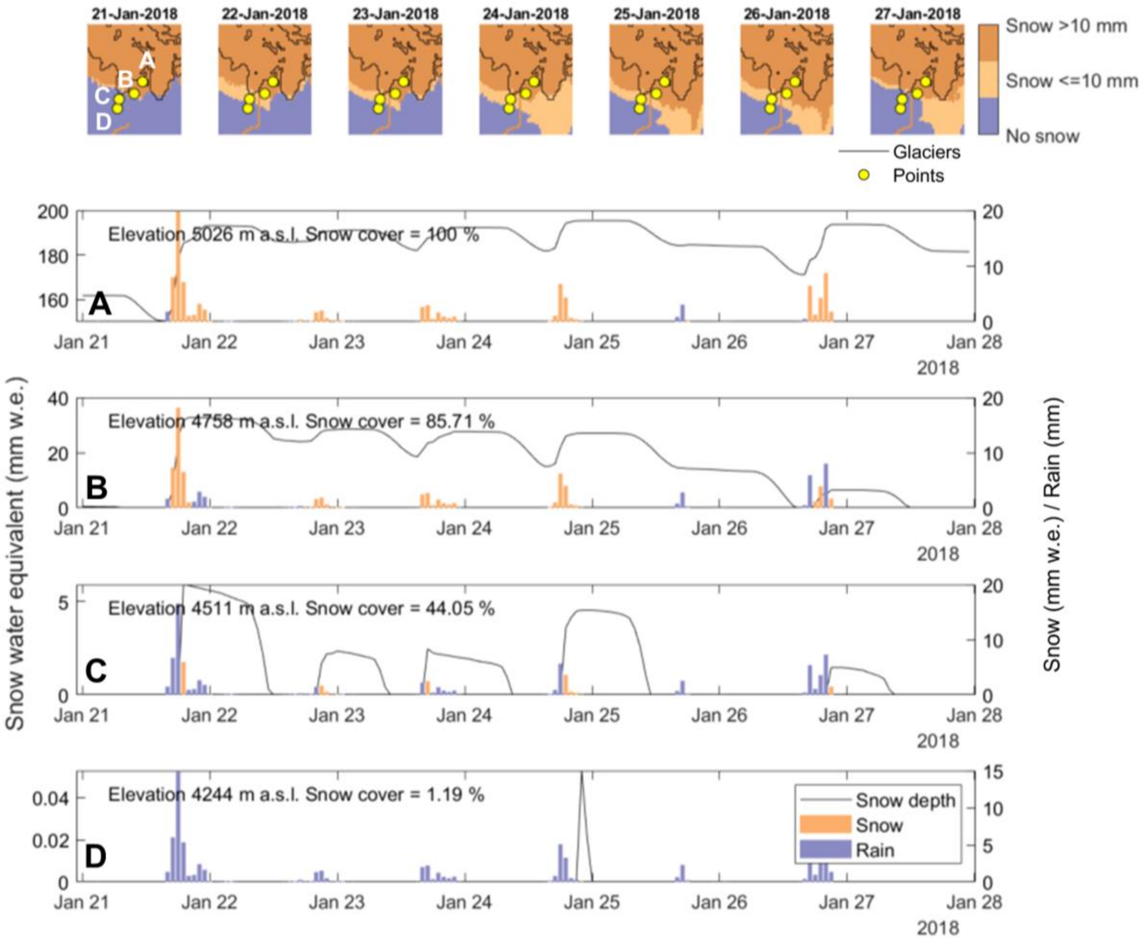


*Supplementary Figure 15 Short term snow dynamics shown with modelled data over an example time period and sub-section of the upper Rio Santa catchment: i) modelled average daily snow cover, classified into no snow (<1 mm w.e.), thin snow (1 to <=10 mm w.e.) and thick snow (>10 mm w.e.) (classification is only for illustration), with glacier outlines shown in black, ii) modelled hourly snow water equivalent and precipitation conditions at four off-glacier point locations (shown in the maps above as yellow points) at a range of elevations. The chosen points were displayed as they show the discontinuous nature of snow cover characteristic of these elevations.*

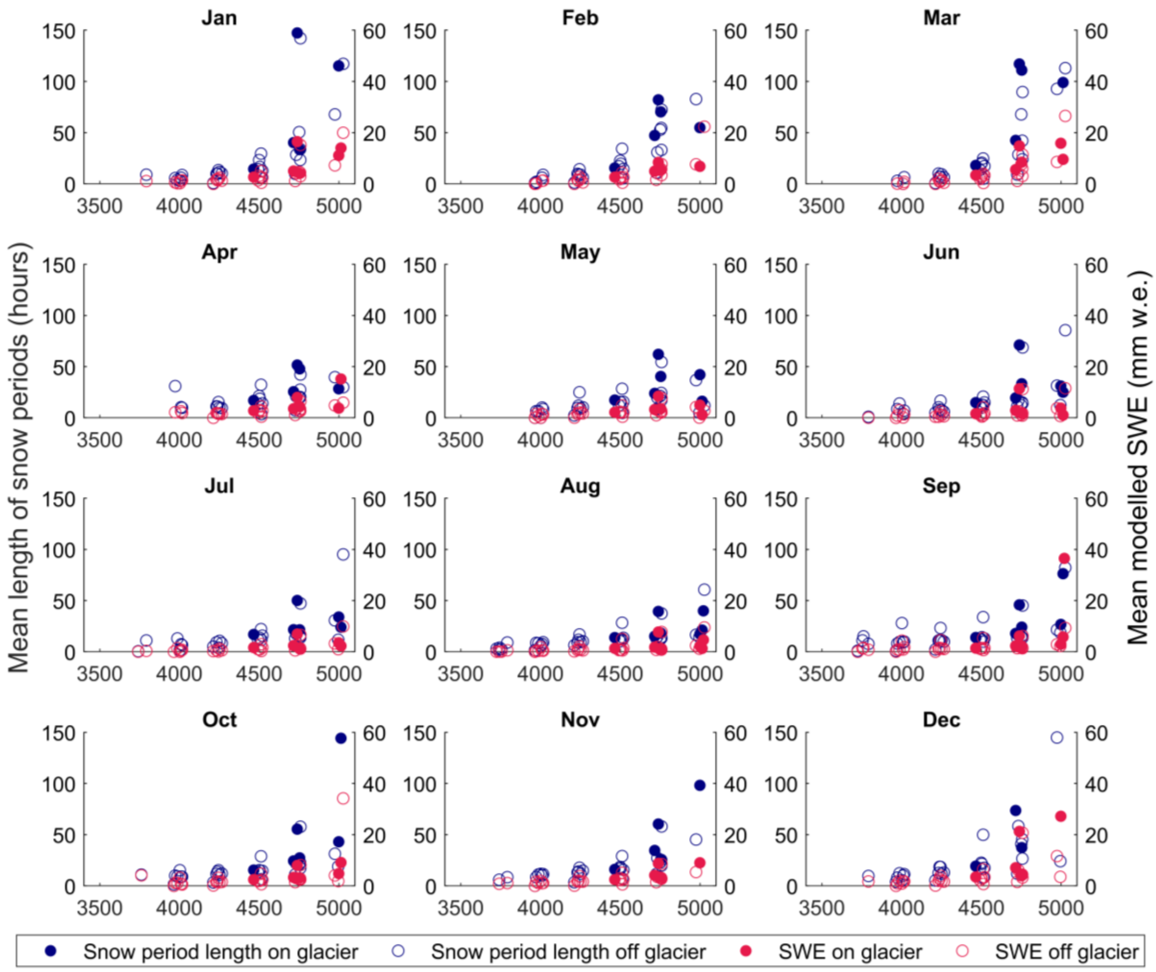


*Supplementary Figure 16 Snow dynamics from hourly data of 84 points taken from transects on both the Blanca and Negra sides of the catchment. Note that the y-axes were limited for visualisation purposes. For each point the mean length of snow periods (so the time from the start to end of a period of snow cover) and the mean snow water equivalent (during the period of snow cover) was calculated. Solid dots are from points on-glacier and hollow dots are from those off-glacier.*

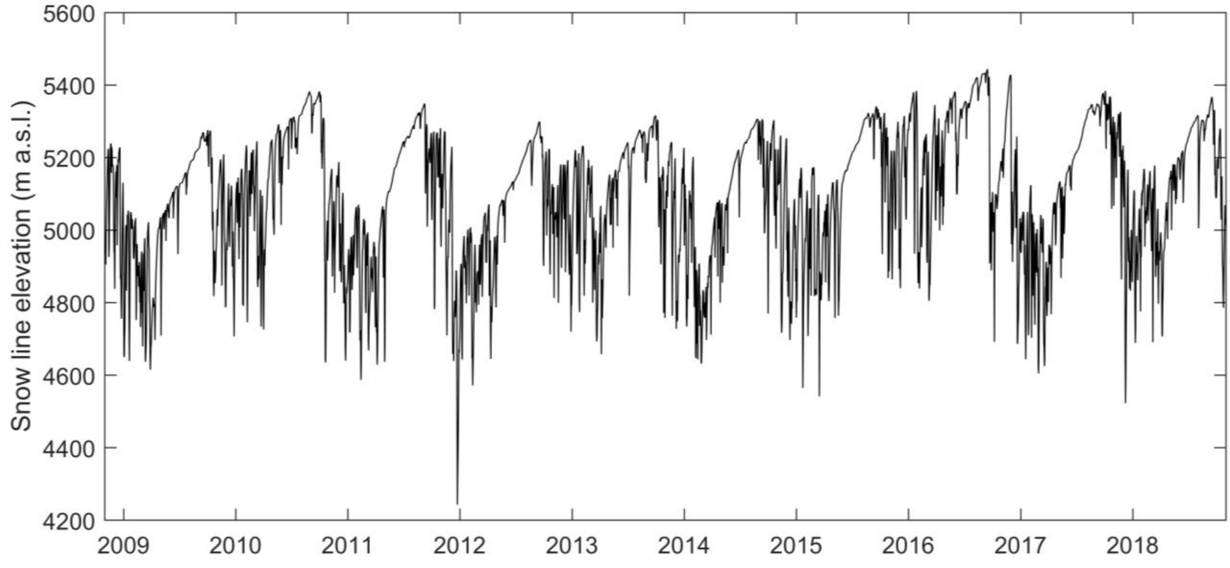


*Supplementary Figure 17 Daily modelled snow line elevation derived for the whole catchment.*

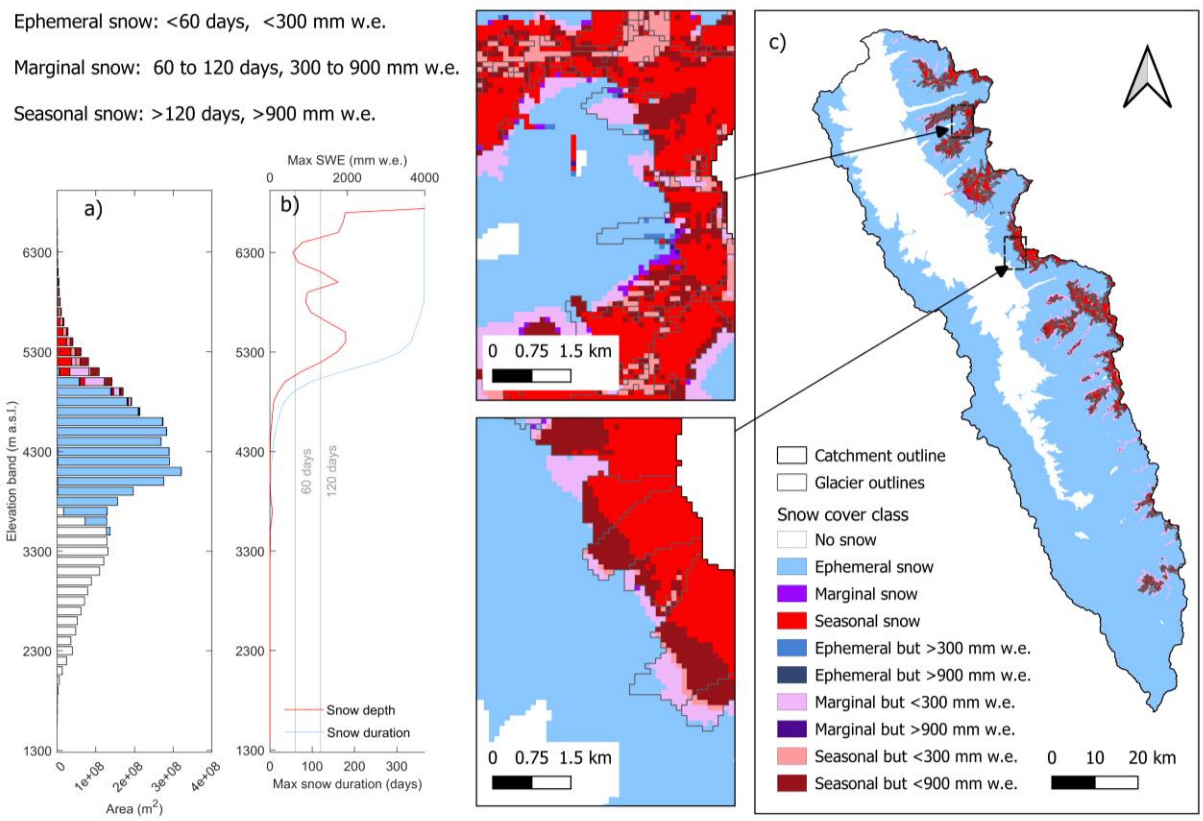


*Supplementary Figure 18 Classification of daily snow maps into ephemeral, marginal and seasonal snow. The classification was based on the average of the modelled maximum hydrological year snow water equivalent and continuous daily snow duration, applying the thresholds of Nadal-Romero and Lopez-Moreno (2024) and Lopez-Moreno et al. (2024). Note we apply the thresholds to values of SWE although the original values are for snow depths (converted from 0.5 m to 300 mm w.e. and 1.5 m to 900 mm w.e., based on the mean density of 24 snow pit measurements on Artesonraju Glacier of 0.6 g cm$^{-3}$). We additionally classify the snow cover if it is ephemeral, marginal or seasonal in duration but has a lower or higher SWE. a) shows the elevation distribution of the snow classification, b) the elevation distribution of the snow water equivalent and snow duration and c) the map of the classification for the Rio Santa catchment with inset maps giving details at two selected locations.*

# 3 Supplementary References


Allen, R.G., Pereira, L.S., Raes, D. and Smith, M. (1998) Crop evapotranspiration — guidelines for computing crop water requirements. *FAO Irrigation and drainage paper 56*. Food and Agriculture Organization, Rome.

Ayala, A., Pellicciotti, F., MacDonell, S., McPhee, J., Vivero, S., Campos, C. and Egli, P. (2016) Modelling the hydrological response of debris-free and debris-covered glaciers to present climatic conditions in the semiarid Andes of central Chile, *Hydrological Processes*, 30, 4036–4058, https://doi.org/10.1002/hyp.10971

Ayala, A., Pellicciotti, F., MacDonell, S., McPhee, J. and Burlando, P. (2017) Patterns of glacier ablation across North-Central Chile: identifying the limits of empirical melt models under sublimation-favorable conditions, *Water Resources Research*, 53, https://doi.org/10.1002/2016WR020126.

Bernhardt, M. and Schulz, K. (2010) SnowSlide: A simple routine for calculating gravitational snow transport, *Geophysical Research Letters*, 37(L11502), https://doi.org/10.1029/2010GL043086

Brock, B. W., Willis, I. C. & Sharp, M. J. (2000). Measurement and parameterisation of albedo variations at Haut Glacier d'Arolla, Switzerland. *Journal of Glaciology*, 46(155), 675-688. https://doi.org/10.3189/172756500781832675

Brock, B. W., Mihalcea, C., Kirkbride, M .P., Diolaiuti, G., Cutler, M. E. J. and Smiraglia, C. (2010) Meteorology and surface energy fluxes in the 2005–2007 ablation seasons at the Miage debris-covered glacier, Mont Blanc Massif, Italian Alps*, J. Geophys. Res.*, 115, D09106, doi:10.1029/2009JD013224

Bureau of Reclamation (2001) *Water Measurement Manual*, US Department of the Interior, Washington

Carenzo, M., Pellicciotti, F., Mabillard, J., Reid, T. and Brock, B. W. (2016) An enhanced temperature index model for debris-covered glaciers accounting for thickness effect, *Advances in Water Resources*, 94, 457-469, http://dx.doi.org/10.1016/j.advwatres.2016.05.001

Carturan, L., Cazorzi, F., De Blasi, F. and Dalla Fontana, G. (2015) Air temperature variability over three glaciers in the Ortles–Cevedale (Italian Alps): effects of glacier fragmentation, comparison of calculation methods, and impacts on mass balance modeling, *The Cryosphere*, 9, 1129-1146, https://doi.org/10.5194/tc-9-1129-2015

Chimner, R. A., Bourgeau-Chavez, L., Grelik, S., Hribljan, J. A., Clarke, A. M. P., Polk, M. H., Lilleskov, E. A. and Fuentealba, B. (2019) Mapping mountain peatlands and wet meadows using multi-date, multi-sensor remote sensing in the Cordillera Blanca, Peru, *Wetlands*, 39, 1057–1067

Ding, B., Yang, K., Qin, J., Wang, L., Chen, Y. & He, X. (2014). The dependence of precipitation types on surface elevation and meteorological conditions and its parameterisation*. Journal of Hydrology*, 513, 154-163. http://doi.org/10.1016/j.jhydrol.2014.03.038

Dussaillant, I., Berthier, E., Brun F., Masiokas, M., Hugonnet, R., Favier, V., Rabatel, A., Pitte, P. and Ruiz, L. (2019) Two decades of glacier mass loss along the Andes, *Nature Geoscience*, 12, 802-808, https://doi.org/10.1038/s41561-019-0432-5

Farinotti, D., Huss, M., Fürst, J. J., Landmann, J., Machguth, H., Maussion, F. and Pandit, A. (2019) A consensus estimate for the ice thickness distribution of all glaciers on Earth, *Nature Geoscience*, 12, 168-173

Fyffe, C. L., Potter, E., Fugger, S., Orr, A., Fatichi, S., Loarte, E., Medina, K., Hellstrom, R. Å., Bernat, M., Aubry-Wake, C., Gurgiser, W., Perry, L. B., Suarez, W., Quincey, D. J., and Pellicciotti, F. (2021) The mass and energy balance of Peruvian glaciers, *Journal of Geophysical Research: Atmospheres*, 126, e2021JD034911. https://doi.org/10.1029/2021JD034911

Greuell, W. and Böhm, R. (1998) 2m temperatures along melting mid-latitude glaciers, and implications for the sensitivity of the mass balance to variations in temperature, *Journal of Glaciology*, 44(146), 9-20, https://doi.org/10.3189/S0022143000002306

Gurgiser, W., June, I., Singer, K., Neuburger, M., Schauwecker, S., Hofer, M. and Kaser, G. (2016) Comparing peasants' perceptions of precipitation change with precipitation records in the tropical Callejón de Huaylas, Peru, *Earth System Dynamics*, 7, 499-515, https://doi.org/10.5194/esd-7-499-2016

Hall, D. K. and Riggs, G. A. (2021) *MODIS/Terra Snow Cover Daily L3 Global 500m SIN Grid, Version 61*, National Snow and Ice Data Centre, https://doi.org/10.5067/MODIS/MOD10A1.061

Härer, S., Bernhardt, M., Siebers, M., and Schulz, K. (2018) On the need for a time- and location-dependent estimation of the NDSI threshold value for reducing existing uncertainties in snow cover maps at different scales, *The Cryosphere*, 12, 1629–1642, https://doi.org/10.5194/tc-12-1629-2018, 2018.

Hellström, R. Å., Fernández, A., Mark, B. G., Covert, J. M., Cochachín, A. and Gomez, R. J. (2017) Incorporating Autonomous Sensors and Climate Modeling to Gain Insight into Seasonal Hydrometeorological Processes within a Tropical Glacierized Valley, *Annals of the American Association of Geographers*, 107(2), 260-273, https://doi.org/10.1080/24694452.2016.1232615

Hugonnet, R., McNabb, R., Berthier, E., Menounos, B., Nuth, C., Girod, L., Farinotti, D., Huss, M., Dussaillant, I., Brun, F. and Kääb, A. (2021). Accelerated global glacier mass loss in the early twenty-first century, *Nature*, 592(7856), 726-731, https://doi.org/10.1038/s41586-021-03436-z

Huss, M., Jouvet, G., Farinotti, D. and Bauder, A. (2010) Future high-mountain hydrology: a new parameterization of glacier retreat, *Hydrology and Earth System Sciences*, 14, 815-829, https://doi.org/10.5194/hess-14-815-2010

INAIGEM (2018) *National Glacier Inventory 2018*, INAIGEM, Huaraz, Peru, https://visor.inaigem.gob.pe/

Khadka, A., Wagnon, P., Brun, F., Shrestha, D., Lejeune, Y. and Arnaud, Y. (2022) Evaluation of ERA5-Land and HARv2 Reanalysis Data at High Elevation in the Upper Dudh Koshi Basin (Everest Region, Nepal), *Journal of Applied Meteorology and Climatology*, 61(8) 931-954

Krajčí, P., Holko, L., Perdigão, R. A. P. and Parajka, J. (2014) Estimation of regional snowline elevation (RSLE) from MODIS images for seasonally snow covered mountain basins*, Journal of Hydrology*, 519, 1769-1778

Lehner, B., Verdin, K. and Jarvis, A. (2008) New Global Hydrography Derived From Spaceborne Elevation Data. *Eos Trans. Am. Geophys. Union,* 89, 93–94

Liu, C., Sun, G., McNulty, S. G., Noormets, A. and Fang, Y. (2017) Environmental controls on seasonal ecosystem evapotranspiration/potential evapotranspiration ratio as determined

by the global eddy flux measurements, *Hydrology and Earth System Sciences*, 21, 311-322, https://doi.org/10.5194/hess-21-311-2017

López-Moreno, J. I., Callow, N., McGowan, H., Webb, R., Schwartz, A., Bilish, S., Revuelto, J., Gascoin, S., Deschamps-Berger, C. and Alonso-González, E. (2024) Marginal snowpacks: The basis for a global definition and existing research needs. *Earth-Sci. Rev.* 252, 104751, https://doi.org/10.1016/j.earscirev.2024.104751

Ministerio del Ambiente (MINAM) (2015) *Mapa Nacional de Cobertura Vegetal*, MINAM, Lima, Peru

Mateo, E. I., Mark, B. G., Hellström, R. A., Baraer, M., McKenzie, J. M., Condom, T., Cochachín, A., Gonzales, G., Quijano, J. and Crúz, R. C. (2022) High-temporal-resolution hydrometeorological data collected in the tropical Cordillera Blanca, Peru (2004–2020), *Earth System Science Data*, 14, 2865–2882, https://doi.org/10.5194/essd-14-2865-2022

Meier, M. F. (1975) Application of remote sensing techniques to the study of seasonal snow cover, *Journal of Glaciology*, 15(73) 251-265

Messager, M. L., Lehner, B., Grill, G., Nedeva, I. and Schmitt, O. (2016) Estimating the volume and age of water stored in global lakes using a geo-statistical approach, *Nature Communications*, 7(13603), https://doi.org/10.1038/ncomms13603

Millan, R., Mouginot, J., Rabatel, A. and Morlighem, M. (2022) Ice velocity and thickness of the world's glaciers, *Nature Geoscience*, 15, 124-129, https://doi.org/10.1038/s41561-021-00885-z

Miles, E., McCarthy, M., Dehecq, A., Kneib, M., Fugger, S., and Pellicciotti, F. (2021). Health and sustainability of glaciers in High Mountain Asia. *Nature Communications*, 12(1), 1-10, https://doi.org/10.1038/s41467-021-23073-4

Muñoz Sabater, J. (2019) ERA5-Land hourly data from 1950 to present. *Copernicus Climate Change Service (C3S) Climate Data Store (CDS)*. https://doi.org/10.24381/cds.e2161bac (Accessed on 26-Sep-2024)

Nadal-Romero, E. and López-Moreno, J. I. (2024) The hydrological response of melting ephemeral snowpacks compared to winter rainfall events in a mid-mountainous Pyrenean catchment. *Mediterr. Geosci. Rev.,* https://doi.org/10.1007/s42990-024-00120-y.

Pappas, C., Fatichi, S. and Burlando, P. (2016) Modeling terrestrial carbon and water dynamics across climatic gradients: does plant trait diversity matter?, *New Phytologist*, 209, 137-151, https://doi.org/10.1111/nph.13590

Parajka, J. and G. Blöschl (2008) Spatio-temporal combination of MODIS images – potential for snow cover mapping. *Water Resour. Res.*, 44, W03406, https://doi.org/10.1029/2007WR006204

Pellicciotti, F., Brock, B. W., Strasser, U., Burlando, P., Funk, M., and Corripio, J. (2005). An enhanced temperature-index glacier melt model including the shortwave radiation balance: development and testing for Haut Glacier d'Arolla, Switzerland. *Journal of Glaciology*, 51(175), 573-587, https://doi.org/10.3189/172756505781829124

Poggio, L., de Sousa, L. M., Batjes, N. H., Heuvelink, G. B. M., Kempen, B., Ribeiro, E., and Rossiter, D. (2021) SoilGrids 2.0: producing soil information for the globe with quantified spatial uncertainty, *SOIL*, 7, 217–240, https://doi.org/10.5194/soil-7-217-2021

Ragettli, S., Pellicciotti, F., Immerzeel, W. W., Miles, E. S., Petersen, L., Heynen, M., Shea, J. M., Stumm, D., Joshi, S. and Shrestha, A. (2015) Unraveling the hydrology of a Himalayan catchment through integration of high resolution in situ data and remote sensing with an advanced simulation model, *Advances in Water Resources*, 78, 94-111, http://dx.doi.org/10.1016/j.advwatres.2015.01.013

Ragettli, S., Cortés, G., McPhee, J. and Pellicciotti, F. (2014) An evaluation of approaches for modelling hydrological processes in high-elevation, glacierized Andean watersheds, *Hydrological Processes*, 28, 5674-5695, https://doi.org/10.1002/hyp.10055

Reid, T. D., Carenzo, M., Pellicciotti, F. and Brock, B. W. (2012) Including debris cover effects in a distributed model of glacier ablation, *Journal of Geophysical Research*, 117, D18105, https://doi.org/0.1029/2012JD017795

RGI Consortium (2017)*. Randolph Glacier Inventory - A Dataset of Global Glacier Outlines*, Version 6. Boulder, Colorado USA. NSIDC: National Snow and Ice Data Center, https://doi.org/10.7265/4m1f-gd79

Rounce, D. R. and McKinney, D. C. (2014) Debris thickness of glaciers in the Everest area (Nepal Himalaya) derived from satellite imagery using a nonlinear energy balance model, *The Cryosphere*, 8, 1317–1329, https://doi.org/10.5194/tc-8-1317-2014.

Sanabria, J. and Lhomme, J. P. (2013) Climate change and potato cropping in the Peruvian Altiplano, *Theoretical and Applied Climatology*, 112, 683-695, https://doi.org/10.1007/s00704-012-0764-1

Saxton, K. E. and Rawls, W. J. (2006) Soil Water Characteristic Estimates by Texture and Organic Matter for Hydrologic Solutions, *Soil Science Society of America Journal*, 70, 1569–1578, https://doi.org/10.2136/sssaj2005.0117

Shaw, T. E., Brock, B. W., Ayala, Á., Rutter, N. and Pellicciotti, F. (2017) Centreline and cross-glacier air temperature variability on an Alpine glacier: assessing temperature distribution methods and their influence on melt model calculations, *Journal of Glaciology*, 63(242), 973–988, https://doi.org/10.1017/jog.2017.65

Shaw, T. E., Buri, P., McCarthy, M., Miles, S. and Pellicciotti, F. (2024) Local controls on near-surface glacier cooling under warm atmospheric conditions, *JGR Atmospheres*, 129(2), e2023JD040214, https://doi.org/10.1029/2023JD040214

Shaw, T. E., Caro, A., Mendoza, P., Ayala, Á., Pellicciotti, F., Gascoin, S. and McPhee, J. (2020) The Utility of Optical Satellite Winter Snow Depths for Initializing a Glacio-Hydrological Model of a High-Elevation, Andean Catchment, *Water Resources Research*, 56, e2020WR027188. https://doi.org/10.1029/2020WR027188

van den Broeke, M., van As, D., Reijmer, C., van de Wal, R. (2004) Assessing and Improving the Quality of Unattended Radiation Observations in Antarctica, *Journal of*

*Atmospheric and Oceanic Technology*, 21, 1417-1431, https://doi.org/10.1175/1520-0426(2004)021<1417:AAITQO>2.0.CO;2

Van Tricht, L., Huybrechts, P., Van Breedam, J., Vanhulle, A., Van Oost, K., Zekollari, H. (2021) Estimating surface mass balance patterns from unoccupied aerial vehicle measurements in the ablation area of the Morteratsch–Pers glacier complex (Switzerland), *The Cryosphere*, 15, 4445–4464, https://doi.org/10.5194/tc-15-4445-2021

Zhang, H., Zhang, F., Zhang, G., Che,T., Yan, W., Ye, M. and Ma, N. (2019) Ground-based evaluation of MODIS snow cover product V6 across China: Implications for the selection of NDSI threshold, *Science of the Total Environment*, 651, 2712-2726, https://doi.org/10.1016/j.scitotenv.2018.10.128
