# Supplementary material for: Thin and ephemeral snow shapes melt and runoff dynamics in the Peruvian Andes
Source: Commun Earth Environ. 2025 Jun 5;6(1):434. doi: 10.1038/s43247-025-02379-x (PMC12141031; doi:10.1038/s43247-025-02379-x)
Supplement: Supplementary file 1 — Transparent Peer Review file [file 43247_2025_2379_MOESM1_ESM.pdf]

nature portfolio

Peer Review File

Thin and ephemeral snow shapes melt and runoff dynamics in the Peruvian Andes

Corresponding Author: Dr Catriona Fyffe

This file contains all editorial decision letters in order by version, followed by all author rebuttals in order by version.

Attachments originally included by the reviewers as part of their assessment can be found at the end of this file.

Version 0:

Decision Letter:

** Please ensure you delete the link to your author home page in this e-mail if you wish to forward it to your coauthors **

Dear Dr Fyffe,

Your manuscript titled "Thin and ephemeral snow shapes melt and runoff dynamics in the Peruvian Andes" has now been seen by 2 reviewers, and we include their comments at the end of this message. They find your work of interest, but some important points are raised. We are interested in the possibility of publishing your study in Communications Earth & Environment, but would like to consider your responses to these concerns and assess a revised manuscript before we make a final decision on publication. Specifically, we ask you to:

1. Clearly acknowledge the limitations of the model, justify key assumptions (such as snowline elevation, snow/rain partitioning), and evaluate the sensitivity of these assumptions, especially in relation to snowline elevation and the impact of thin snow on downstream hydrology.

2. Explicitly acknowledge the uncertainty in the study, particularly given the limited data and potential issues with validation, and attempt to better quantify the uncertainty in the results.

We therefore invite you to revise and resubmit your manuscript, along with a point-by-point response that takes into account the points raised. Please highlight all changes in the manuscript text file.

Please submit your point-by-point responses as a separate file, distinct from your cover letter where you can add responses to the Editors' comments that you do not want to be made available to the reviewers. Word files are preferred. We recommend that any figures, tables or graphs that are included in the response to reviewers are also included in the main article or Supplementary Information.

We are committed to providing a fair and constructive peer-review process. Please don't hesitate to contact us if you wish to discuss the revision in more detail.

Please use the following link to submit your revised manuscript, point-by-point response to the referees' comments (which should be in a separate document to any cover letter), a tracked-changes version of the manuscript (as a PDF file) and the completed checklist:
Link Redacted
** This url links to your confidential home page and associated information about manuscripts you may have submitted or be reviewing for us. If you wish to forward this email to co-authors, please delete the link to your homepage first **

We hope to receive your revised paper within six weeks; please let us know if you aren't able to submit it within this time so that we can discuss how best to proceed. If we don't hear from you, and the revision process takes significantly longer, we may close your file. In this event, we will still be happy to reconsider your paper at a later date, as long as nothing similar has been accepted for publication at Communications Earth & Environment or published elsewhere in the meantime.

Please do not hesitate to contact us if you have any questions or would like to discuss these revisions further. We look

forward to seeing the revised manuscript and thank you for the opportunity to review your work.

Best regards,

Alireza Bahadori, PhD
Associate Editor
Communications Earth & Environment

Claudia Zoccarato, PhD
Editorial Board Member
Communications Earth & Environment
orcid.org/0000-0002-3199-8681

EDITORIAL POLICIES AND FORMATTING

We ask that you ensure your manuscript complies with our editorial policies. Please ensure that the following formatting requirements are met, and any checklist relevant to your research is completed and uploaded as a Related Manuscript file type with the revised article.

Editorial Policy: Policy requirements
(Download the link to your computer as a PDF.)

For Manuscripts that fall into the following fields:
• Behavioural and social science
• Ecological, evolutionary & environmental sciences
• Life sciences
An updated and completed version of our Reporting Summary must be uploaded with the revised manuscript
You can download the form here:
https://www.nature.com/documents/nr-reporting-summary.zip

Furthermore, please align your manuscript with our format requirements, which are summarized on the following checklist:
Communications Earth & Environment formatting checklist

and also in our style and formatting guide Communications Earth & Environment formatting guide .

*** DATA: Communications Earth & Environment endorses the principles of the Enabling FAIR data project (http://www.copdess.org/enabling-fair-data-project/). We ask authors to make the data that support their conclusions available in permanent, publically accessible data repositories. (Please contact the editor if you are unable to make your data available).

All Communications Earth & Environment manuscripts must include a section titled "Data Availability" at the end of the Methods section or main text (if no Methods). More information on this policy, is available at http://www.nature.com/authors/policies/data/data-availability-statements-data-citations.pdf.

In particular, the Data availability statement should include:
- Unique identifiers (such as DOIs and hyperlinks for datasets in public repositories)
- Accession codes where appropriate
- If applicable, a statement regarding data available with restrictions
- If a dataset has a Digital Object Identifier (DOI) as its unique identifier, we strongly encourage including this in the Reference list and citing the dataset in the Data Availability Statement.

DATA SOURCES: All new data associated with the paper should be placed in a persistent repository where they can be freely and enduringly accessed. We recommend submitting the data to discipline-specific, community-recognized repositories, where possible and a list of recommended repositories is provided at http://www.nature.com/sdata/policies/repositories.

If a community resource is unavailable, data can be submitted to generalist repositories such as figshare or Dryad Digital Repository. Please provide a unique identifier for the data (for example a DOI or a permanent URL) in the data availability statement, if possible. If the repository does not provide identifiers, we encourage authors to supply the search terms that will return the data. For data that have been obtained from publically available sources, please provide a URL and the specific data product name in the data availability statement. Data with a DOI should be further cited in the methods reference section.

Please refer to our data policies at http://www.nature.com/authors/policies/availability.html.

REVIEWER COMMENTS:

Reviewer #1 (Remarks to the Author):

What are the major claims of the paper?

- snow melt contribution to runoff is substantial in the Peruvian Andes
- ice melt is crucial to runoff in the dry season

Are they novel and will they be of interest to others in the community and the wider field?

- Many studies in the international scientific literature emphasize the role of glaciers as water resources but the role of seasonal snow is often overlooked. This paper nicely shows that the seasonal snow is an important driver of the regional water cycle and that it should be better studied.

Is the work convincing, and if not, what further evidence would be required to strengthen the conclusions?

- The work is convincing because it deals with a large region and the model is well calibrated against river flow datasets and remote sensing products for snow and glaciers. The model outputs are very well presented.

Do you feel that the paper will influence thinking in the field?

- Yes, the field of snow and glacier hydrology in tropical regions

The ability of a researcher to reproduce the work, given the level of detail provided.

- The model parameters are well described. The TOPKAPI hydrological model is a closed source software but it may be possible to reproduce the work with a trial license. More specifically I am not sure if the TOPKAPI-ETH model that was used by the authors is the same model or a different version, and if it can be obtained online? There is no information about this in the paper. Ideally the model code (or indications on how to obtain it) and data should be shared along with the paper.

General comments

This study used a semi-empirical snow model, which does not account for processes like sublimation or energy balance. This is not obvious in the main manuscript, as the methodological details are given in a very long supplementary. The limitations of the model could be better acknowledged and discussed. Lejeune et al. 2007 studied the "physical processes involved in the melting and disappearance of transient snow cover in nonglacierized tropical areas" and demonstrated that horizontal energy transfers play a key role. As far as I understood the TOPKAPI model does not account for energy storage in the snowpack hence melt starts as soon as the air temperature exceeds a calibrated threshold (TT). This may work well in areas where the snowpack is isothermal but may not work as well at higher elevations where the "cold content" of the snowpack is large.

A point that should be clarified throughout the manuscript is the definition of "contribution". Here the authors used the ratio of snow melt / liquid water inputs to characterize the snow contribution (liquid water inputs: rainfall, snow melt, ice melt). Therefore it should not be confused with a snow melt contribution to river flow or runoff, because a fraction of these liquid water inputs are subject to evaporation and groundwater also contributes to river discharge. For example, the caption of Fig. 4 is not correct in my opinion "pie charts showing the proportional contribution of rain, snow melt and ice melt to the runoff" I suspect that the authors meant "liquid water inputs" instead of "runoff". See L252 too.

The snowline elevation was computed based on the assumption that "the snow preferentially covers higher rather than lower elevation". This assumption should be justified/discussed. It amounts to neglecting processes like avalanches which are actually simulated by the model (the snowline elevation is usually determined using the method of Krajčí et al., 2014). Additionally, the threshold of 10 mm we to convert SWE to snow cover should be discussed since it is higher than the "thin snow" definition threshold. It means that the simulated thin snow was actually not observed, whereas it forms the core of the paper concept. Another key parameter is the snow/rain partition temperature threshold. The sensitivity of the modelled snowline elevation to these parameters could be evaluated.

About the significance of thin snow in the Upper Rio Santa from a water resource perspective in a changing climate: the authors showed that thin snow can represent a significant amount of water, but they also showed that it melts within a few hours. Therefore, how much does it matter in downstream regions if it is snow or if it is rain? Other catchment processes like subsurface flow can smooth rainfall runoff at similar timescales. The hydrological signature of thin snowmelt could become indistinguishable in catchments with large concentration time (> 1 day). The authors could use their model to investigate how far downstream such snowmelt signature is actually significant.

Minor comments

L92: What were the conclusions of these previous studies? What were their limitations?

L198: "demonstrating" is strong. Both regions have different elevation ranges therefore the discharge differences could also be due to differences in seasonal snow storage.

L278: I did not understand the rationale of using ERA5-Land temperature (without downscaling) to characterize the snow across the Peruvian Andes since ERA5-Land snow water equivalent is available?

Supp Eq 1: revise the equation

Throughout the manuscript: snowcover vs. snow cover

Reviewer #2 (Remarks to the Author):

Dear authors,
I have read with interest your manuscript and I think is an interesting and novel contribution aiming to quantify the role of ephemeral snowpacks on the hydrology of Cordillera Blanca. Snow in these regions is a part of the water balance often neglected and that the work demonstrate that it is an important one. The manuscript is well written and structured and the overall quality of the presentation is good. My main concern is the very limited data available to perform a correct evaluation of the simulations. Validation is not very robust and prevent to offer ranges of uncertainty. The use of MODIS images to validate the model is also a bit uncertain as the high cloudiness during the wet season also introduce important uncertainty in the estimations. Said that, I think this should not be a major objection to accept the publication of the work, but perhaps uncertainty should be at least recognised in the discussion, and if possible a bit better quantified. The rest of my comments are mostly minor and authors can consider to take them into account when preparing a revised version.

- Line 8, abstract: instead of saying "substantial" I would provide numbers for the wet and dry season.
- As recognised in SI, model gives nin-negligible errors when estimating snow line elevation. This should be stated and discussed.

- The title of the the 1st subsection of results is a bit contradictory with the stated in the second sentence (lines 131-132). Title: "wet season snowpack confined to high elevation"; sentence:"at low elevation snow is ephemeral and confined to wet season". I would rephrase to avid the small conclusion.
- I really like figure 2, may be it would be good to indicate the mean elevation of ELA of glaciers in the region to better appreciate the processes in the ablation and accumulation areas.
- line 167: (from on and off glacier areas respectively).
- I am not very convinced with figure 4 as is. Maybe it would be better to show only the pie charts for C. Blanca and Negra for wet and dry seasons, having more space to enlarge the plots to represent the fraction of inputs according to distance from the outlet. The maps could me moved as SI.
- I try to avoid to recommend publications from my group when acting as reviewer but just consider if these ones serves to support the uncertainties on runoff contribution of shallow and dynamic snowpacks (1), and evidence of the role of ephemeral snowpacks to shape hydrographs (2).:
1-https://doi.org/10.1016/j.earscirev.2024.104751
2-https://doi.org/10.1007/s42990-024-00120-y

This other reference (3) also inform about the hydrological relevance of ephemera snowpacks:
3-https://doi.org/10.5194/hess-22-4891-2018

- Figure SI14: Part of the y-axis of the left plots is missing.

Hoping my comments will help,

Ignacio López-Moreno

** Visit Nature Research's author and referees' website at www.nature.com/authors for information about policies, services and author benefits**

Communications Earth & Environment is committed to improving transparency in authorship. As part of our efforts in this direction, we are now requesting that all authors identified as ‘corresponding author’ create and link their Open Researcher and Contributor Identifier (ORCID) with their account on the Manuscript Tracking System prior to acceptance. ORCID helps the scientific community achieve unambiguous attribution of all scholarly contributions. You can create and link your ORCID from the home page of the Manuscript Tracking System by clicking on ‘Modify my Springer Nature account’ and following the instructions in the link below. Please also inform all co-authors that they can add their ORCIDs to their accounts and that they must do so prior to acceptance.

https://www.springernature.com/gp/researchers/orcid/orcid-for-nature-research

For more information please visit http://www.springernature.com/orcid

If you experience problems in linking your ORCID, please contact the Platform Support Helpdesk.

Version 1:

Decision Letter:

** Please ensure you delete the link to your author home page in this e-mail if you wish to forward it to your coauthors **

Dear Dr Fyffe,

Your revised manuscript titled "Thin and ephemeral snow shapes melt and runoff dynamics in the Peruvian Andes" has now been seen by our reviewers, whose comments appear below. In light of their advice we are delighted to say that we are happy, in principle, to publish a suitably revised version in Communications Earth & Environment.

We therefore invite you to revise your paper one last time to comply with our format requirements and to maximise the accessibility and therefore the impact of your work.

EDITORIAL REQUESTS:

Please review our specific editorial comments and requests regarding your manuscript in the attached "Editorial Requests Table".

*****Please take care to match our formatting and policy requirements. We will check revised manuscript and return manuscripts that do not comply. Such requests will lead to delays. *****

Please outline your response to each request in the right hand column. Please upload the completed table with your manuscript files as a Related Manuscript file.

If you have any questions or concerns about any of our requests, please do not hesitate to contact me.

SUBMISSION INFORMATION:

In order to accept your paper, we require the files listed at the end of the Editorial Requests Table; the list of required files is also available at https://www.nature.com/documents/commsj-file-checklist.pdf .

OPEN ACCESS:

Communications Earth & Environment is a fully open access journal. Articles are made freely accessible on publication. For further information about article processing charges, open access funding, and advice and support from Nature Research, please visit https://www.nature.com/commsenv/open-access

At acceptance, you will be provided with instructions for completing the open access licence agreement on behalf of all authors. This grants us the necessary permissions to publish your paper. Additionally, you will be asked to declare that all required third party permissions have been obtained, and to provide billing information in order to pay the article-processing charge (APC).

Please use the following link to submit the above items:
Link Redacted
** This url links to your confidential home page and associated information about manuscripts you may have submitted or be reviewing for us. If you wish to forward this email to co-authors, please delete the link to your homepage first **

We hope to hear from you within two weeks; please let us know if you need more time.

Best regards,

Claudia Zoccarato, PhD
Editorial Board Member
Communications Earth & Environment
orcid.org/0000-0002-3199-8681

Alireza Bahadori, PhD

Associate Editor
Communications Earth & Environment
Consulting Editor
Communications Sustainability

REVIEWERS' COMMENTS:

Reviewer #1 (Remarks to the Author):

The authors have done a great job to answer my comments. I found their responses very convincing. The additional analyses strengthen their conclusions and the discussion is more balanced with respect to the model uncertainties.

Reviewer #2 (Remarks to the Author):

Dear authors,
I have read the revised manuscript and in my opinion, my main concerns and suggestions have been adequately addressed and the manuscript has gained in robustness and better estimation of uncertainties. Thus, I am happy to suggest the acceptance in its current form.

Best regards,
Ignacio López

** Visit Nature Research's author and referees' website at www.nature.com/authors for information about policies, services and author benefits**

Open Access This Peer Review File is licensed under a Creative Commons Attribution 4.0 International License, which permits use, sharing, adaptation, distribution and reproduction in any medium or format, as long as you give appropriate credit to the original author(s) and the source, provide a link to the Creative Commons license, and indicate if changes were made.
In cases where reviewers are anonymous, credit should be given to 'Anonymous Referee' and the source.
The images or other third party material in this Peer Review File are included in the article’s Creative Commons license, unless indicated otherwise in a credit line to the material. If material is not included in the article’s Creative Commons license and your intended use is not permitted by statutory regulation or exceeds the permitted use, you will need to obtain permission directly from the copyright holder.
To view a copy of this license, visit https://creativecommons.org/licenses/by/4.0/

Revision Statement of Paper 'Thin and ephemeral snow shapes melt and runoff dynamics in the Peruvian Andes' by Fyffe et al. for submission to Communications Earth and Environment

RESPONSE TO REVIEWERS AND GENERAL REVISION

Dear Reviewers

We would like to thank the reviewers for their thorough and useful reviews. Both recognise the novelty and contribution of the paper to the understanding of the hydrology of the Peruvian Andes. The reviewers' comments have contributed to greatly improve and strengthen the manuscript, and we are very grateful for their constructive comments.

We summarise here the main concerns of both reviewers and the changes to the revised manuscript we have taken in response:

1) **Need to better quantify and discuss model uncertainty and limitations** (raised by both reviewers, see R1C1 and R2C2). We added a new Discussion section 'Opportunities and challenges in modelling the tropical cryosphere' which includes a discussion of the impact of the precipitation threshold temperature (which leverages new model runs and the new SI Figure 14) on the fractional contribution of melt inputs and a discussion on the model limitations, specifically in the calculation of melt and influence of sublimation, cold content and other patchy snow processes.

2) **Need to better quantify the error in the derivation of modelled snowline elevations** (raised by both reviewers in R1C3 and R2C4). We added error bars to the modelled snowlines in Figure 1d representing the range due to variations in the threshold to define a cell as snow-covered, and we included an additional SI Figure 11 showing the influence of varying the threshold temperature between snow and rain on the modelled snowlines. In addition we added text in the main paper in the section on 'Model skill in simulating snow and glacier processes'.

3) **Better discussion of the hydrological impact of ephemeral snow** (raised by reviewer 1 in R1C4). Here we added text in the discussion section ' The importance of ephemeral snow in the tropical Andean water cycle' which mentions the potential ecohydrological impact of ephemeral snow and the difficulties in separating snow and rain events in our catchment with such a large elevation range.

4) **Improved clarity in the derivation of snow cover across Peru** (raised by reviewer 1 in R1C7). We included ERA-5 Land wet season percentage snow cover derived from the SWE data into Figure 5 in the main text, and additional explanation of our reasoning for deriving the percentage snow cover from air temperature in SI section 8.

5) **Inclusion of additional literature dealing with classification of snow types** (raised by reviewer 2 in R2C9). We included additional text in the discussion section 'The importance of ephemeral snow in the tropical Andean water cycle' which assesses the snow cover in the catchment in relation to the classification of ephemeral, marginal and seasonal snow, informed by new SI Figure 18.

We have also made numerous other minor changes to the text and Figures in response to the reviewers comments, which are detailed below. We would also like to mention that we found a

mistake in the implementation of the Ding et al. (2014) precipitation partition, which resulted in a change in our calibrated precipitation partition temperature threshold (from 1.9°C to 2.2°C). We have updated the paper to take account of this correction throughout, but we note that the influence on the results is minor (changes in the melt contribution to inputs are around 1-2%) and none of the major results and conclusions change as a result.

We would like to extend our thanks again to the reviewers for their very helpful and constructive comments.

With kind regards,

Catriona Fyffe

(on behalf of all co-authors)

Please note that the reviewer responses are in black and our responses are in blue.

REVIEWER COMMENTS:

Reviewer #1 (Remarks to the Author):

What are the major claims of the paper?

- snow melt contribution to runoff is substantial in the Peruvian Andes
- ice melt is crucial to runoff in the dry season

Are they novel and will they be of interest to others in the community and the wider field?

- Many studies in the international scientific literature emphasize the role of glaciers as water resources but the role of seasonal snow is often overlooked. This paper nicely shows that the seasonal snow is an important driver of the regional water cycle and that it should be better studied.

Is the work convincing, and if not, what further evidence would be required to strengthen the conclusions?

- The work is convincing because it deals with a large region and the model is well calibrated against river flow datasets and remote sensing products for snow and glaciers. The model outputs are very well presented.

Do you feel that the paper will influence thinking in the field?

- Yes, the field of snow and glacier hydrology in tropical regions

The ability of a researcher to reproduce the work, given the level of detail provided.

- The model parameters are well described. The TOPKAPI hydrological model is a closed

source software but it may be possible to reproduce the work with a trial license. More specifically I am not sure if the TOPKAPI-ETH model that was used by the authors is the same model or a different version, and if it can be obtained online? There is no information about this in the paper. Ideally the model code (or indications on how to obtain it) and data should be shared along with the paper.

We thank the reviewer again for their useful and constructive review! We appreciate very much that the reviewer believes our work is convincing and will be a novel contribution to the field of tropical glacier hydrology.

As for the last point regarding the availability of software and model code, we want to clarify here that, upon publication we will make the TOPKAPI-ETH software used to conduct the modelling, alongside all the necessary analysis code and model outputs freely available within the NERC Environmental Information Data Centre (EIDC). This is mentioned in the Code and Data Availability sections of the manuscript.

General comments

R1C1: This study used a semi-empirical snow model, which does not account for processes like sublimation or energy balance. This is not obvious in the main manuscript, as the methodological details are given in a very long supplementary. The limitations of the model could be better acknowledged and discussed. Lejeune et al. 2007 studied the "physical processes involved in the melting and disappearance of transient snow cover in nonglacierized tropical areas" and demonstrated that horizontal energy transfers play a key role. As far as I understood the TOPKAPI model does not account for energy storage in the snowpack hence melt starts as soon as the air temperature exceeds a calibrated threshold (TT). This may work well in areas where the snowpack is isothermal but may not work as well at higher elevations where the "cold content" of the snowpack is large.

We thank the reviewer for this point. We do state in the Overview section of the Methodology that the model uses an enhanced Temperature index (ETI) melt model approach. We also would like to mention that this approach is recognised to be in between EB and simple empirical approaches (such as a degree day model). It includes the shortwave radiation balance, which has been demonstrated to be the largest energy flux for melt in most climates, but does not indeed calculate sublimation nor the cold content of the snow or ice. We do understand the limitations of not using a full energy balance approach, but we made significant efforts to calibrate the ETI model carefully against energy balance modelling at several sites at a range of elevations at the point scale (SI section 3.2). At the higher sites, and especially in the dry season, the snow or ice pack can develop cold content, which influences the timing of melt. However, since these processes were captured in the energy balance modelling they were implicitly accounted for during the ETI calibration processes. Importantly, the intermediate nature of the model has allowed us to conduct high temporal and spatial resolution modelling across the upper Rio Santa - so far full energy balance approaches have only been applied at the glacier or small catchment scale due to their computational constraints. In order to make clearer the main model limitations, we added a new section ‘Opportunities and challenges in modelling the tropical cryosphere’ into the discussion. This now clearly states the type of melt model and mentions that we cannot quantify sublimation nor the variable impact of the cold content explicitly, nor processes like the ground heat flux and horizontal energy transfers (lines

330-344).

R1C2: A point that should be clarified throughout the manuscript is the definition of "contribution". Here the authors used the ratio of snow melt / liquid water inputs to characterize the snow contribution (liquid water inputs: rainfall, snow melt, ice melt). Therefore it should not be confused with a snow melt contribution to river flow or runoff, because a fraction of these liquid water inputs are subject to evaporation and groundwater also contributes to river discharge. For example, the caption of Fig. 4 is not correct in my opinion "pie charts showing the proportional contribution of rain, snow melt and ice melt to the runoff" I suspect that the authors meant "liquid water inputs" instead of "runoff". See L252 too.

This is a very fair point and we did not intend to cause any confusion. The reviewer is correct that we define the contribution of snow melt/ice melt and rain as a proportion of the total of these three liquid inputs, which we regard as the input to the catchment, or hydrological system - not to river runoff.

We have thoroughly checked and modified the manuscript to ensure the text is more precise in this regard. See L38 and 40 (Abstract), L122 (Introduction), L159, L169, L173, L184, L189, L200, L210 (Results), L220 (Fig. 3 caption), L231 and L233 (Figure 4 caption), L260, L293 (Discussion), L397, L406, L410 (Conclusion) and L431 (Methodology).

Note that in some places for brevity, and when talking more generally (e.g. in the introduction), we have kept the phrase ‘contribution of melt to runoff’, but when giving results from our modelling we have used the more precise terminology of ‘melt contribution to [liquid water] inputs’ or similar.

R1C3: The snowline elevation was computed based on the assumption that "the snow preferentially covers higher rather than lower elevation". This assumption should be justified/discussed. It amounts to neglecting processes like avalanches which are actually simulated by the model (the snowline elevation is usually determined using the method of Krajčí et al., 2014). Additionally, the threshold of 10 mm we to convert SWE to snow cover should be discussed since it is higher than the "thin snow" definition threshold. It means that the simulated thin snow was actually not observed, whereas it forms the core of the paper concept. Another key parameter is the snow/rain partition temperature threshold. The sensitivity of the modelled snowline elevation to these parameters could be evaluated.

We thank the reviewer for the comment, which is very useful, and to address which we carried out a number of additional simulations and considerations. Before presenting them, we first want to clarify a couple of points in regards to the computation of the snowline elevation. We will discuss each point in turn for clarity:

1. Calculation of snowline elevation and snow cover outputs

We want to make clear first of all that the way the snowline elevation is calculated does not influence the modelled snow cover, as shown in Figure 1d, and it is only important for the comparison of the modelled snowcover with that derived from MODIS imagery. The model outputs SWE in a gridded way on a daily timescale, and at the point scale at an hourly timescale. Therefore in the analysis in the results section of the paper we use the modelled SWE as directly output by the model – the calculation of the snowline is not needed nor relevant

for the operation or calibration of the model itself, and is only acting as an independent evaluation of snow variability with elevation in the catchment.

2. Method used to compute the snowline elevation

We thank the reviewer for mentioning that we did not give a source for our method to compute the snowline elevation in the comparison between the MODIS and modelled snowcover. The method used is based on that mentioned by Meier (1975) who suggested to use '*the area measurement [of snow cover] and relate this to an area/altitude of a drainage basin or mountain and from that determining the equivalent snow-line altitude (ESA)*'. We have now added that citation into SI section 4.1 (lines 339-352). When initially comparing MODIS and modelled snowcover we also applied the Krajčí et al. (2014) method. This however requires setting a minimum snow cover threshold (Ps_min) (usually 5%) below which images for a day are discarded. Given that we already excluded glacier areas from the comparison, and that snow cover in the dry season off-glacier can be very low, including this threshold resulted in the removal of a large proportion of the images. As demonstrated by Fig. 6 in Krajčí et al. (2014) a reduction of this threshold tended to reduce the accuracy of the snowline elevation, and therefore this method is not well suited to catchment areas with small proportional snow-covered areas. The Meier (1975) method seemed therefore more appropriate for our work. For comparison we show the Krajčí et al. (2014) method without any Ps_min filter below (Figure R1). This shows a lower RMSE and higher bias than with the Meier (1975) method but the model is still able to replicate the seasonal and annual patterns in snowline elevation, mainly within the classification error of the imagery.

3. 10 mm threshold

Firstly, the 10 mm w.e. threshold to determine whether a cell was snow-covered or not also only influences the comparison of the modelled and MODIS snowcover and does not influence the modelled snowcover shown in the paper. Note also that the thin snow threshold of 10 mm w.e. that we apply in SI Figure 15 is only to simplify the snowcover maps shown in part i)- it is therefore only for illustrative purposes. We apply the 10 mm w.e. threshold to the modelled SWE data since we presume that very thin snow may be patchy in reality and therefore may not be classified as snow-covered in the classification of the MODIS imagery (as the snow might not sufficiently influence the spectral signature so that it is classified as snow). We chose the 10 mm w.e. threshold based on Parajka and Blöschl (2008) who found that the biases when comparing measured snow depths with MODIS snow cover were smallest with a 1 cm snow depth threshold, but that the MODIS accuracy was not sensitive to thresholds between 1 and 3 cm (which we equate to ≅ 6 to 18 mm w.e., applying a snow density of 0.6 g cm^{-3}, derived from the mean value across 24 snow pit measurements on Artesonraju Glacier (unpublished data)).

However, we also conducted additional analysis to understand the impact of the threshold on the comparison with the MODIS snowcover data (Figure R2). We applied a conservative range of the threshold of 1 to 20 mm w.e. to cover the range of snow depths mentioned by Parajka and Blöschl (2008). There is little difference in the calculated snowline (see Figure R2) between applying a 10 or 20 mm w.e. threshold but the modelled snow line elevation can be lower than MODIS when applying a 1 mm w.e. threshold. These differences are most pronounced during particular months in the wet season, and are much smaller in the dry season when the snowline is gradually rising. Note the magnitude of the snowline variability is similar to that of the MODIS data uncertainty due to variations in the NDSI threshold. We would not expect MODIS to be able

to capture the dynamics of the thinnest snow cover variations due to both its spatial and temporal resolution. For this reason we also compare against snow cover at three points from albedo data (SI Figure 12). Here we allow any depth of snow to count as snow-covered since the scale of comparison is much smaller and patchiness is less of a problem.

In addition we added in the discussion the need to better observe the dynamics of the ephemeral snow, e.g. using trail cameras and temperature sensors (lines 255-258).

To summarise, we agree with the reviewer that the 10 mm w.e. threshold can influence the modelled snowlines, but we have shown above why this threshold is appropriate, ascertained the impact of varying it on the snowlines, and included this analysis within Figure 1d.

4. Sensitivity to snow/rain partition threshold

In order to investigate the influence of the rain/snow partition threshold on the modelled snowline elevations we conducted **additional full model runs** using the minimum and maximum calibrated temperature thresholds (1.2°C and 4.9°C, see SI section 3.1). This has allowed us to assess the variation in the snowline caused by varying this modelled parameter, see Figure R3. This shows that the snowline elevation is relatively insensitive to this threshold temperature in the dry season, as the snowline evolution is controlled by melt processes. There are differences, as expected, in the wet season, when the higher threshold temperature gives lower modelled snowline elevations. Using the Meier (1975) snowline method, the PrecSF value of 1.2°C gives the lowest RMSE while the bias is lowest with a PrecSF of 4.9°C but we think this may be due to the very slightly lower dry season snowlines reducing the model overestimation at this time of year, and compensating for the model underestimation of snowlines in the wet season. The applied PrecSF of 2.2°C gives a good balance of low bias and RMSE between the values of the other thresholds. We also analysed how varying the precipitation partition temperature varied the fraction of ice and snow melt of total inputs, see R2C2 for a full response. Overall, we agree with the reviewer that the PrecSF value can alter the modelled snowlines, but we have now quantified this and added the analysis into SI Figure 11.

Resulting changes to the paper in relation to R1C3

As a result of the comprehensive analyses described above we applied the following changes to the paper:

- included error bars representing the variation in the threshold used to define snow-covered cells in Figure 1d. Note that the snowline range due to PrecSF changes is within this range.
- included in the caption of Figure 1d that we use the Meier (1975) method and explain the error bars included
- added a new Figure 11 into the SI which shows the comparison of the snowlines with the variations in the PrecSF value, along with mention in SI section 6.3 of this sensitivity analysis.
- added text in the section 'Model skill in simulating snow and glacier processes' section in the main paper (in Methodology) which mentions that the modelled snowlines are influenced by the choice of SWE threshold and signposts to the PrecSF analysis in the SI: *'The modelled snowlines are verified through good correspondence with MODIS data, with a small bias of 24.3 m and an RMSE of 140 m (Figure 1d and SI Section 6.3). The model does tend to overestimate the snowline elevation in the dry season and*

underestimate it in the wet season, but it replicates the multiannual patterns of snowline evolution well. Modelled snowlines are influenced by the choice of SWE threshold used to define a cell as snow-covered (represented by the error bars in Figure 1d), but the magnitude of the uncertainty is similar to that in the MODIS data itself (defined by the NDSI threshold). We tested the influence of the threshold temperature between snow and rain on the modelled snowlines (SI Figure 11), finding that differences were significant only in wet season months and that the chosen threshold of 2.2˚C performed best. ' (lines 475-484)

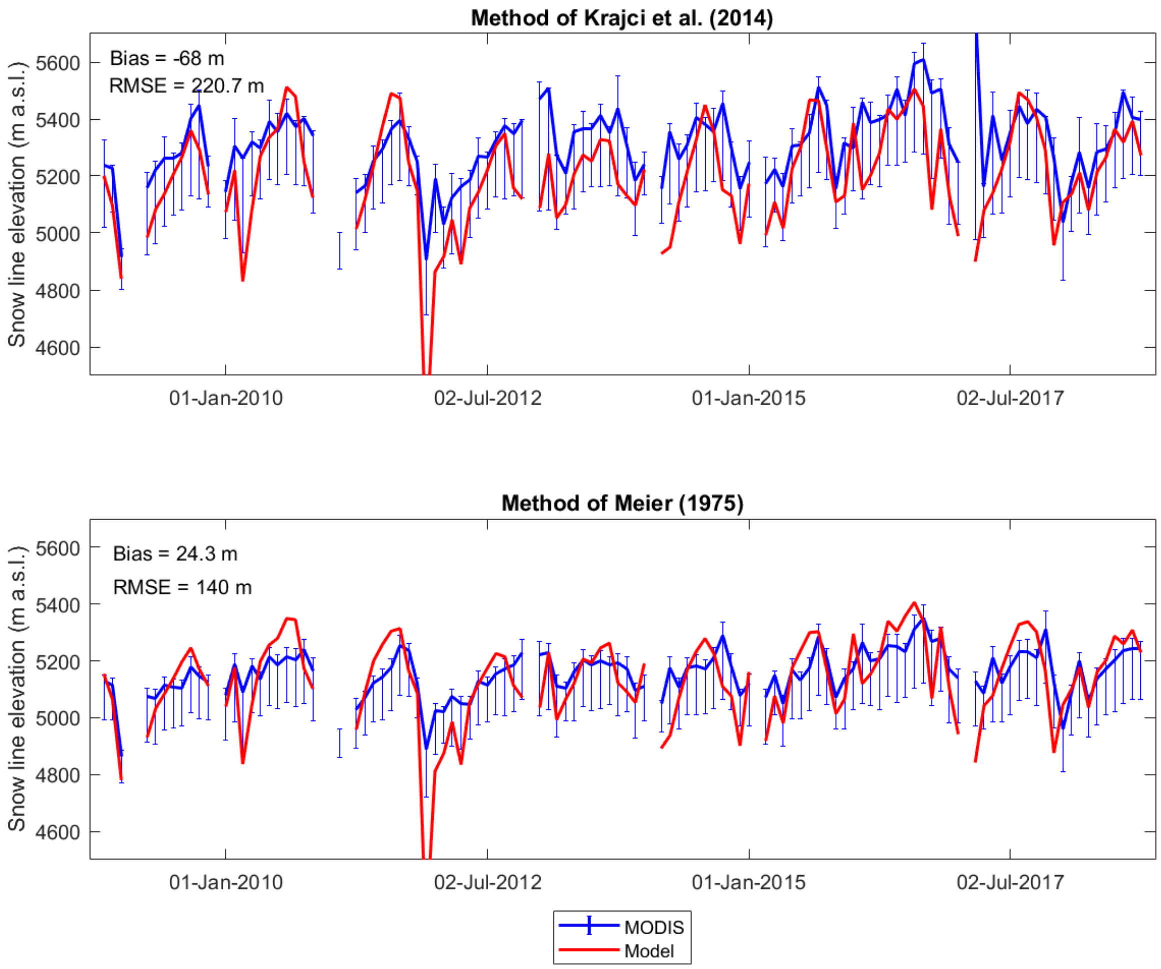

Figure R1 Comparison of modelled and MODIS snowline elevations derived from different methods. Top panel uses the method of Krajčí et al. (2014) (without applying a Ps_min filter) and the lower panel shows the method of Meier (1975). The MODIS error bars represent the snowline derived using alternate NDSI threshold values of 0.1 (minimum) and 0.45 (maximum).

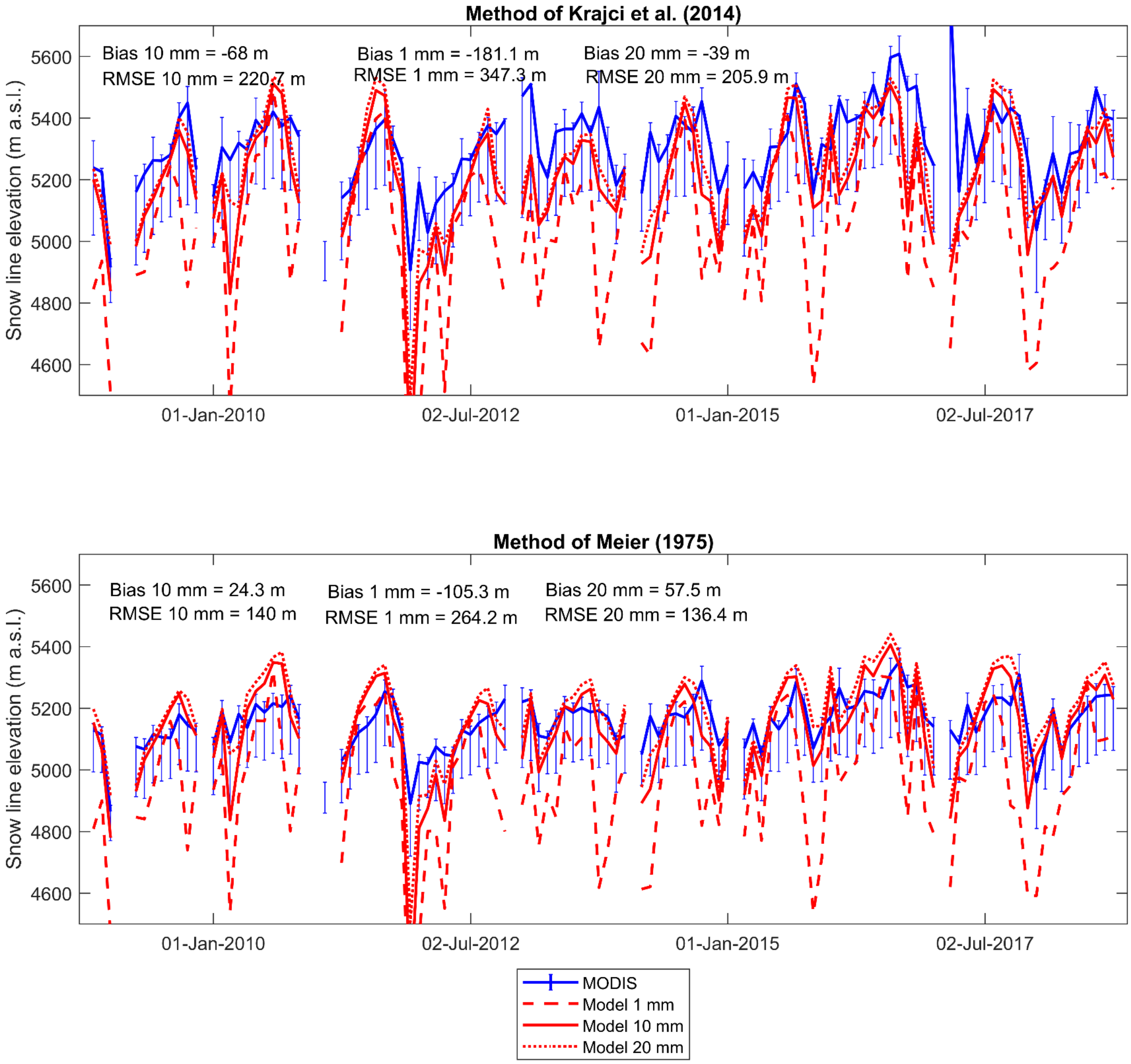

Figure R2 Comparison of modelled and MODIS snowline elevations derived from different methods. Top panel uses the method of Krajčí et al. (2014) (without applying a Ps_min filter) and the lower panel shows the method of Meier (1975). The MODIS error bars represent the snowline derived using alternate NDSI threshold values of 0.1 (minimum) and 0.45 (maximum). The different model estimates are derived by using varying thresholds (in mm w.e.) to define a cell as snow-covered.

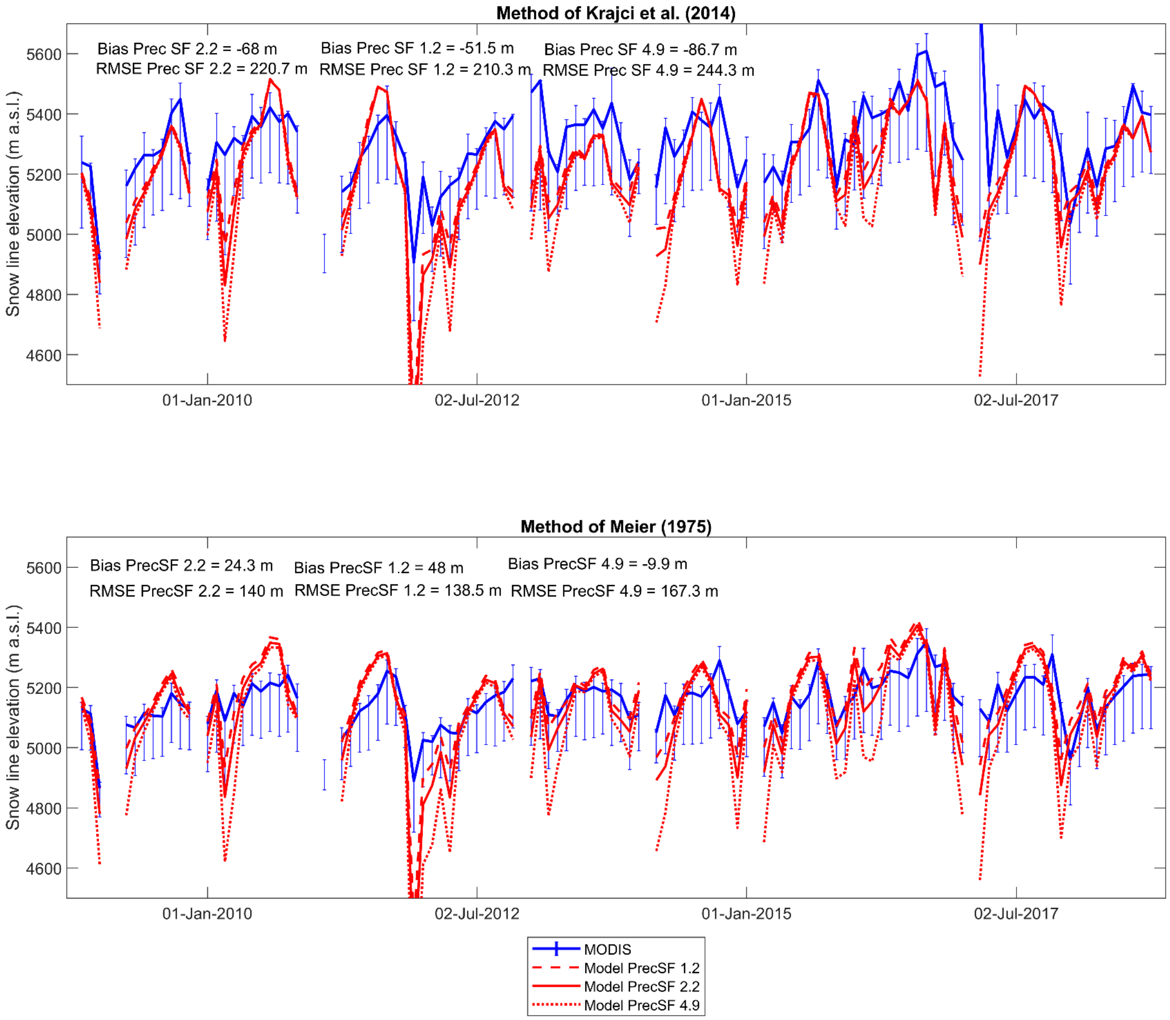

Figure R3 Comparison of modelled snowline elevations with variations in the threshold temperature between snow and rain (PrecSF).

R1C4: About the significance of thin snow in the Upper Rio Santa from a water resource perspective in a changing climate: the authors showed that thin snow can represent a significant amount of water, but they also showed that it melts within a few hours. Therefore, how much does it matter in downstream regions if it is snow or if it is rain? Other catchment processes like subsurface flow can smooth rainfall runoff at similar timescales. The hydrological signature of thin snowmelt could become indistinguishable in catchments with large concentration time (> 1 day). The authors could use their model to investigate how far downstream such snowmelt signature is actually significant.

We really appreciate the author's comments here, as this is a great consideration and one that we have also been pondering for a while. But although it would seem that we could easily determine with the model the hydrological significance of the melting of the thin snow as opposed to rain, it is actually very difficult to disentangle. This is because there is a very steep elevation gradient in the catchment, which means that there are rarely events which are either

snow or rain, to allow a comparison of flow pathways and responses, and even if an event is mainly rain, there is often still snow high in the catchment which can melt. As an example we show model outputs for December 2009 (Figure R4) where most precipitation events have both snow and rain components, and there is still snow melt on days of only rain. There is usually a very strong diurnal pattern in the wet season of daytime snow (and sometimes ice) melt, followed by precipitation in the afternoon which will be rain at lower elevations and snow higher up. Looking higher in the catchment just makes it more difficult to isolate rain only events. So we cannot easily split rain and snow melt events by time and separately analyse their hydrological signal. This is in contrast to studies such as Nadal-Romero and Lopez-Moreno (2024) where winter rain events could be distinguished from those with a snow melt component. It is therefore very challenging to a) separate the runoff signal of only snow melt versus rain and b) even more tricky to separate specifically the influence of the melt of thin snow compared to seasonal snow on the runoff signal. We think this is **a very fascinating area** of further study but one which is difficult to address well under the current climate/elevation characteristics of our catchment. We do however have confidence that the ephemeral snow would impact **the glacier albedo** (and hence glacier melt) and in general influence the catchment energy budget, even if we cannot explicitly distinguish the impacts of the thin snow melt on the hydrograph in this study.

In order to address this comment we therefore changed the discussion section ‘The importance of ephemeral snow in the tropical Andean water cycle’ to a) include the difficulty of separating hydrographs from rain and snow in our modelling and b) added reference to the literature on existing studies on the impact of ephemeral snow on soil moisture infiltration and runoff (lines 269-284)

Differences in infiltration of snow melt compared to rain can also lead to impacts on soil moisture and hence on the partitioning of water into runoff and evapotranspiration[43], as well as their subsequent hydrological pathways. Previous work has suggested that snow melt is more effective at recharging groundwater than rainfall[44,45], but understanding of snowmelt recharge is concentrated in regions with seasonal snowpacks. Current findings specific to ephemeral snow are contradictory: in a Mediterranean climate Nadal-Romero and López-Moreno[36] found infiltration of ephemeral snow events was lower than from rain, with shorter flood hydrograph lag times, related to frozen soil and impermeable substrates; whereas in Arizona, Dwivedi et al.[46] found increased soil percolation at ephemeral snow sites, compared to seasonal sites, due to melt occurring in mid-winter when evapotranspiration is lower. Disentangling the impact of ephemeral snow on the hydrograph from our modelling in the Rio Santa catchment is complicated, since the large catchment elevation range results in precipitation events being composed of both snow and rain simultaneously at different elevations. Understanding the impact of snow melt on soil moisture and recharge in regions of intermittent snowpacks and warm-season snow accumulation is lacking[47] and this calls for more research to investigate these processes.

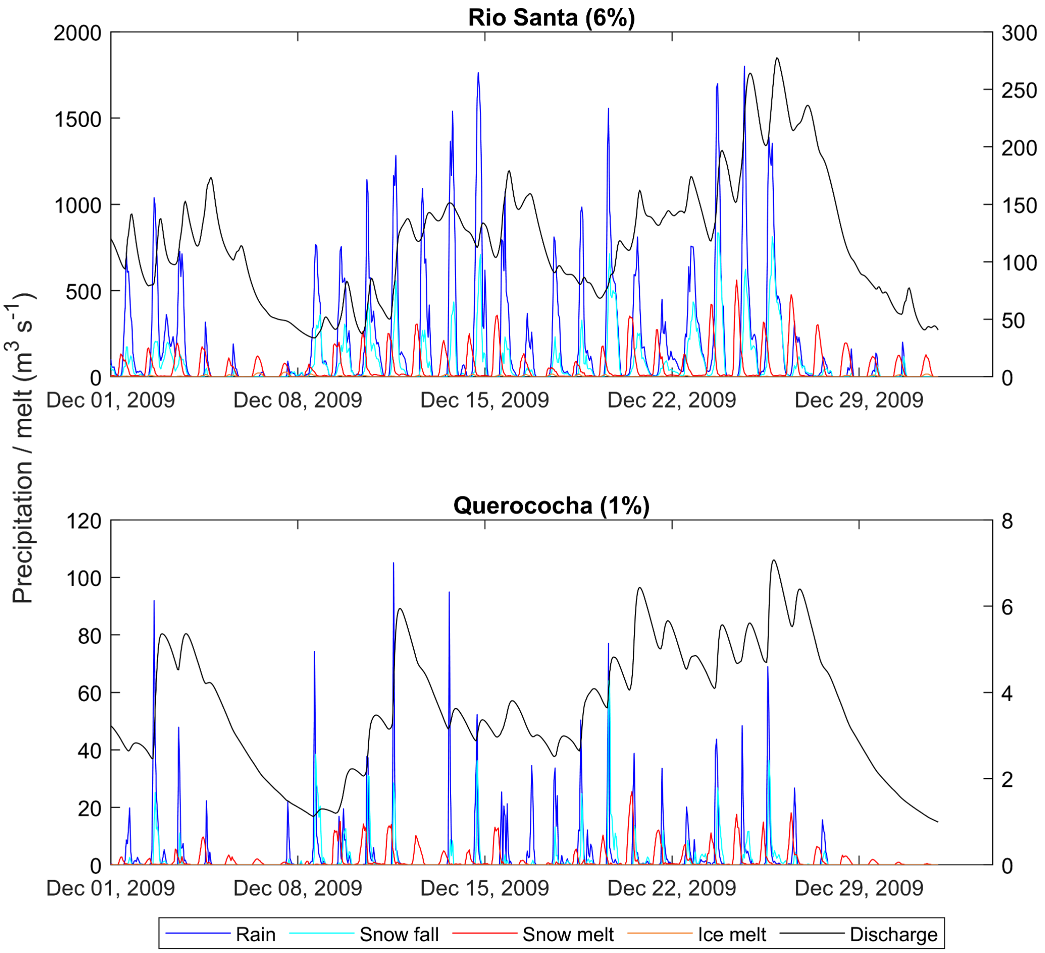

Figure R4 Hourly modelled outputs for the upper Rio Santa (upper plot) and Querococha subcatchment (lower plot) for December 2009. These show that most precipitation events are composed of a mixture of rain and snowfall, and that catchment inputs in a given day are also composed of a mixture or rain and snow and ice melt. It would therefore be difficult to characterise hydrographs as being composed of mainly rain or snow melt signatures.

Minor comments

R1C5: L92: What were the conclusions of these previous studies? What were their limitations?

We have added more details here about the conclusions and limitations of Condom et al. (2012) and Aubry-Wake et al. (2024), see lines 95-103.

R1C6: L198: "demonstrating" is strong. Both regions have different elevation ranges therefore the discharge differences could also be due to differences in seasonal snow storage.
In this section talking about the runoff from both sides of the catchment, our view is that the larger dry season specific discharges on the Blanca side are a result of both snow and ice melt (so we agree with the reviewer that the difference could be due to seasonal snow storage, not just ice melt). We have therefore kept the 'demonstrating' but been more specific to include ice and snow: *'In the dry season, however, there is a clear divide between the lack of runoff on the non-glacierised Negra side and the much higher contribution from the glacierised Blanca side*

(Figure 4), demonstrating that snow and ice melt from high elevations is vital for dry season discharge.'

R1C7: L278: I did not understand the rationale of using ERA5-Land temperature (without downscaling) to characterize the snow across the Peruvian Andes since ERA5-Land snow water equivalent is available?
We decided to use the ERA5-Land temperature (linked to our results) rather than the ERA5-Land SWE since in general we would expect that ERA5-Land temperature patterns would be more robustly represented than precipitation (e.g. Khadka et al. (2022), and this is also our experience from using ERA5-Land as model forcing in other projects). By using our model results we were therefore able to use the link between air temperature and percentage of time with wet season snow cover to do this. However, we have now also included the wet season percentage snow cover directly from ERA5-Land in Figure 5 for comparison and included text in SI section 8 which explains the rationale for deriving the snow cover from the ERA-5 Land air temperature. The direct ERA5-Land snow cover shows a larger area with >10% snow cover, but there are no areas with >50% snow cover. We also show here for your interest (Figure R5) the comparison with the ERA5-Land mean wet season SWE. It can be seen that there is a very large area with very thin mean snow depths (<1 mm w.e.) but that the areas of thicker mean SWE also correspond with those derived from the ERA5 air temperatures.

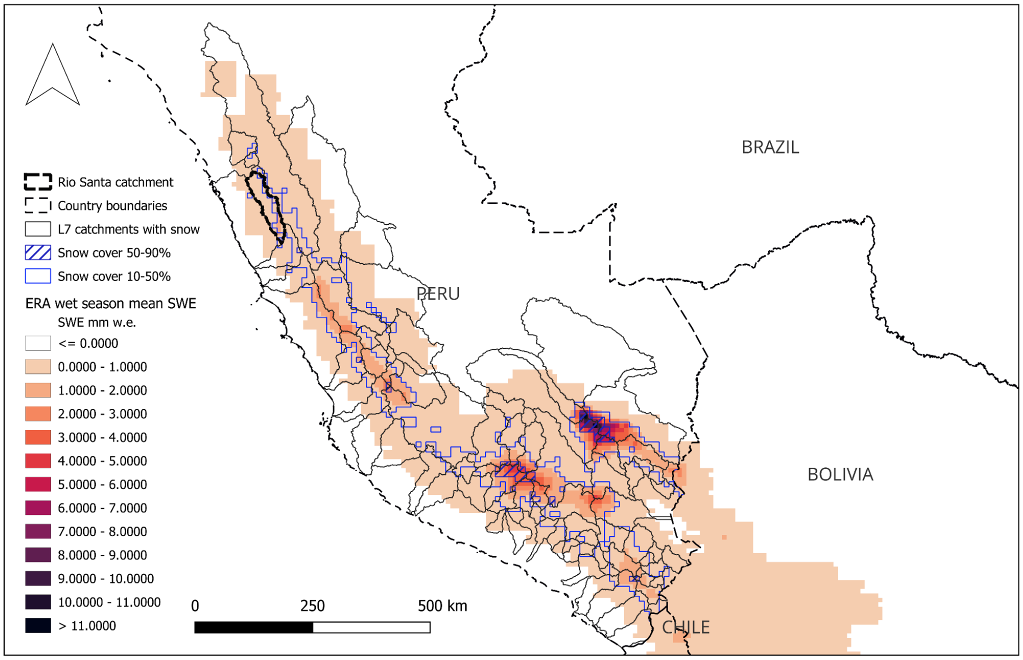

Figure R5 Comparison of ERA-5 Land mean wet season SWE with the snow cover areas derived from the ERA5-Land air temperatures and the relationship developed between the modelled air temperatures and percentage wet season snow cover.

R1C8: Supp Eq 1: revise the equation
Thanks for spotting the error, this has been fixed.

R1C9: Throughout the manuscript: snowcover vs. snow cover
Thanks for pointing this out, we changed to 'snow cover' throughout the manuscript and SI.

References

Khadka, A., Wagnon, P., Brun, F., Shrestha, D., Lejeune, Y. and Arnaud, Y. (2022) Evaluation of ERA5-Land and HARv2 Reanalysis Data at High Elevation in the Upper Dudh Koshi Basin (Everest Region, Nepal), *Journal of Applied Meteorology and Climatology*, 61(8) 931-954

Krajčí, P., Holko, L., Perdigão, R. A. P. and Parajka, J. (2014) Estimation of regional snowline elevation (RSLE) from MODIS images for seasonally snow covered mountain basins, *Journal of Hydrology*, 519, 1769-1778

Meier, M .F. (1975) Application of remote sensing techniques to the study of seasonal snow cover, *Journal of Glaciology*, 15(73) 251-265

Parajka, J., and G. Blöschl (2008), Spatio-temporal combination of MODIS images – potential for snow cover mapping, *Water Resour. Res.*, 44, W03406, doi:10.1029/2007WR006204.

Reviewer #2 (Remarks to the Author):

Dear authors,
R2C1: I have read with interest your manuscript and I think is an interesting and novel contribution aiming to quantify the role of ephemeral snowpacks on the hydrology of Cordillera Blanca. Snow in these regions is a part of the water balance often neglected and that the work demonstrate that it is an important one. The manuscript is well written and structured and the overall quality of the presentation is good.

We thank the reviewer for their kind comment on the contribution of the manuscript.

R2C2: My main concern is the very limited data available to perform a correct evaluation of the simulations. Validation is not very robust and prevent to offer ranges of uncertainty. The use of MODIS images to validate the model is also a bit uncertain as the high cloudiness during the wet season also introduce important uncertainty in the estimations. Said that, I think this should not be a major objection to accept the publication of the work, but perhaps uncertainty should be at least recognised in the discussion, and if possible a bit better quantified. The rest of my comments are mostly minor and authors can consider to take them into account when preparing a revised version.

1. Validation of the model

We thank the reviewer for their important points here. In regards to the use of the MODIS imagery we would refer to our detailed answer in response to R1C3 (above). In addition, we understand the limitations of MODIS imagery in the wet season and did remove images marked with a high cloudiness (>0.8), and only compared modelled data with MODIS when there was suitable MODIS imagery available.

We also want to mention here however that we included a large range of independent validation methods to understand the performance of the model, and we would not entirely agree that we used “very limited data” for validation. Alongside the MODIS imagery we also compared **point-based snow cover derived from albedo** measurements (SI Figure 12 and Table 7). We also compared the model against **altitudinally resolved glacier mass balances at the full catchment and sub-catchment scale** (Figure 1c and SI Figure 7), noting that these mass

balance data are novel in themselves and were derived for this study. This is something that has never been done previously in this region - and for very few regions in general. We include an estimate of the remote sensing uncertainty in these figures, following the method of Miles et al. (2021). In addition we compare against **measured stake** and **snow pit measurements at three glaciers** (SI Figure 9), as well as **river discharge at five sites** (Figure 10). We feel this is a very comprehensive evaluation of the model, especially considering that none of these data sets were used for calibration.

Nevertheless we realised that we did not include a measurement of the uncertainty of the discharge measurements so we have included an estimate of the discharge measurement uncertainty in SI Figure 10 (based on the rating curve mean absolute percentage errors). We also added a statement about the uncertainty of the ablation stake measurements in the caption of SI Figure 9, but did not show these as error bars as they are very small relative to the y-axis scale.

2. Uncertainty of the model

We think it's a useful point made by the reviewer to better discuss the model uncertainty. In the submitted manuscript we included the results of a parameter sensitivity assessment within the methodology and within the SI (section 6.4). Due to computational demands of the model it is unfortunately not possible to run a full Monte Carlo uncertainty analysis. However, from our sensitivity analysis the two key parameters that influenced the results were TT (the threshold temperature to allow melt) and PrecSF (the threshold temperature between snow and rain). Although TT is quite influential we note that it was calibrated alongside the other ETI model parameters against energy balance melt modelling (see SI section 3.2), so we have confidence that they are suitable for this region and climate characteristics. The PrecSF value was calibrated against a more advanced precipitation phase method (Ding et al. 2014) but the range in suitable threshold temperatures is quite large due to the large elevation range in the catchment. We are also aware that the amount of snow modelled is quite important to the findings of our study. For this reason we conducted **additional full model runs** with the minimum and maximum calibrated temperature thresholds (1.2°C and 4.9°C, respectively). This has allowed us to fully assess the impact of this parameter range on the results of the fractions of ice and snow melt of total inputs. We have added a discussion of this into the new section 'Opportunities and challenges in modelling the tropical cryosphere' in the discussion of the main paper.

The results of this analysis (shown in new SI Figure 14) show that although with the lower threshold applied there is a reduction in the wet season fraction of snow melt of total inputs, it is by only -6% on average and the difference in the fraction of snowmelt decreases into the dry season, since the amount of snowmelt is more dependant on melt rates themselves than the precipitation fraction of snow or rain. Conversely, at the higher temperature threshold the % snow melt in the wet season could be much larger (by on average +18%), although we note that the elevations that this temperature threshold are suitable for (>5100 m a.s.l.) include a relatively small proportion of the catchment (albeit an important elevation range for glacier processes). The proportion of ice melt is also rather insensitive to changes in the precipitation threshold. This is a little surprising, but is because although there is a substantial increase in ice melt in the wet season under the lower temperature threshold (due to less snow), at this time of year the fractional contribution of ice melt is very small relative to that from snow or rain, and in

the dry season the amount of ice melt barely changes since the precipitation phase is less important (as the ice melt rates are more important).

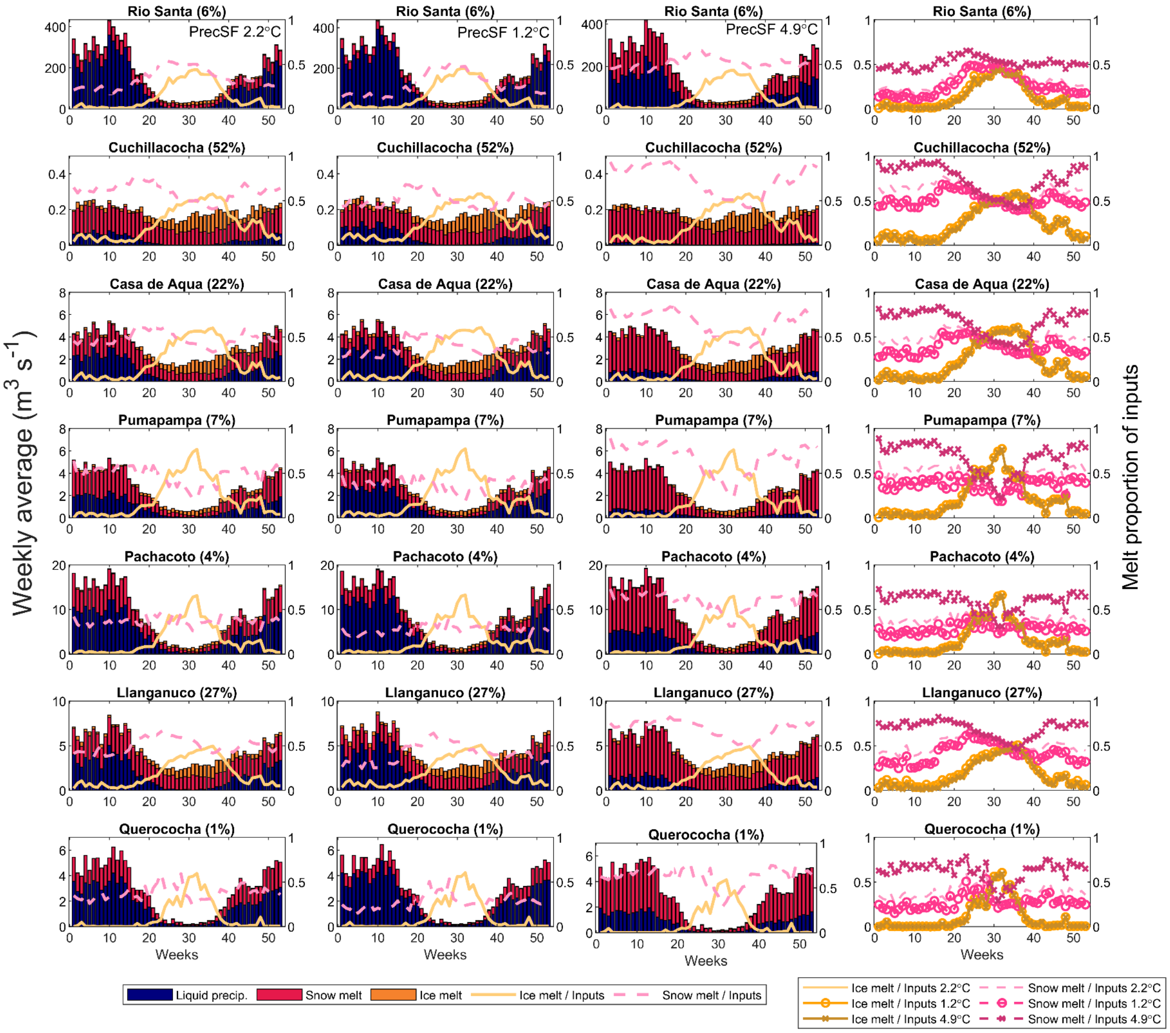

SI Figure 14 Sensitivity assessment of the amount and fractional contribution of liquid precipitation, snow melt and ice melt contributions to total inputs into the catchment to a change in the temperature threshold between snow and rain (PrecSF). The first column contains the model results with the threshold as applied in the main paper (2.2°C), the second column is with a threshold of 1.2°C and the third column with a threshold on 4.9°C (see SI section 3.1 for details of the calibration of these thresholds). The fourth column contains a comparison of the fractions of ice and snowmelt of total inputs for the three temperature thresholds.

R2C3: - Line 8, abstract: instead of saying "substantial" I would provide numbers for the wet and dry season.

We have added the wet and dry season average % values for the contribution of ice melt, and

for consistency also the maximum % contribution of ice melt in the next sentence (Lines 38 and 40).

R2C4: - As recognised in SI, model gives nin-negligible errors when estimating snow line elevation. This should be stated and discussed.

We discuss in detail the comparison of the modelled and MODIS snow line elevations in response to R1C3. Specifically in relation to this point we added additional text in the section 'Model skill in simulating snow and glacier processes' (lines 475-478)which mentions the model overestimation in the dry season and underestimation in the wet season compared to MODIS.

R2C5: - The title of the the 1st subsection of results is a bit contradictory with the stated in the second sentence (lines 131-132). Title: "wet season snowpack confined to high elevation"; sentence:"at low elevation snow is ephemeral and confined to wet season". I would rephrase to avid the small conclusion.
This is a good point about the potential confusion here and we have renamed the results section to focus on the elevation difference in the snow cover only, to simplify the message: 'Lower elevation snow cover is thin and ephemeral with seasonal snowpacks confined to high elevations'

R2C6: - I really like figure 2, may be it would be good to indicate the mean elevation of ELA of glaciers in the region to better appreciate the processes in the ablation and accumulation areas.

We are glad that the reviewer likes this figure overall. We already included the mean ELA in panel b) but we have added this to the other panels to help with the interpretation.

R2C7: - line 167: (from on and off glacier areas respectively).

Here we do mean that the snowpack contributes melt from both the on and off-glacier areas together, however, to reduce any confusion we have rephrased the sentence to *'The snow dynamics in the Peruvian Andes are therefore characterised by i) a highly ephemeral, thin snowpack below 5000 m a.s.l., which contributes melt in the wet season from both on and off-glacier areas, ii) and a higher elevation snowpack, mostly confined to glaciers, which provides a contribution to catchment inputs in the dry season.'* See lines 169-173.

R2C8: - I am not very convinced with figure 4 as is. Maybe it would be better to show only the pie charts for C. Blanca and Negra for wet and dry seasons, having more space to enlarge the plots to represent the fraction of inputs according to distance from the outlet. The maps could me moved as SI.

We understand here that the fraction of the input sub-plots were quite small, but we did want to keep the maps and their pie charts in the main paper. For this reason we adjusted the plot layout for Figure 4 to allow the line plots to be larger.

R2C9: - I try to avoid to recommend publications from my group when acting as reviewer but just consider if these ones serves to support the uncertainties on runoff contribution of shallow and dynamic snowpacks (1), and evidence of the role of ephemeral snowpacks to shape hydrographs (2).:
1-https://doi.org/10.1016/j.earscirev.2024.104751
2-https://doi.org/10.1007/s42990-024-00120-y

R2C10: This other reference (3) also inform about the hydrological relevance of ephemera snowpacks:
3-https://doi.org/10.5194/hess-22-4891-2018

We are very grateful to the reviewer here for pointing out these references. We found them very interesting and they inspired us to conduct some additional analysis. We used the daily modelled outputs of the snow water equivalent to classify the snowcover into ephemeral, marginal and seasonal snow, following the definitions in Nadal-Romero and Lopez-Moreno (2024) and Lopez-Moreno et al. (2024) and using the average of the modelled maximum hydrological year snow water equivalent and continuous daily snow duration. Note we apply the thresholds to values of SWE, although the original values are for snow depths, converting using a mean snow density value from snow pits on Artesonraju Glacier. We created a new SI Figure 18 and added a mention of this into the discussion (lines 246-255).

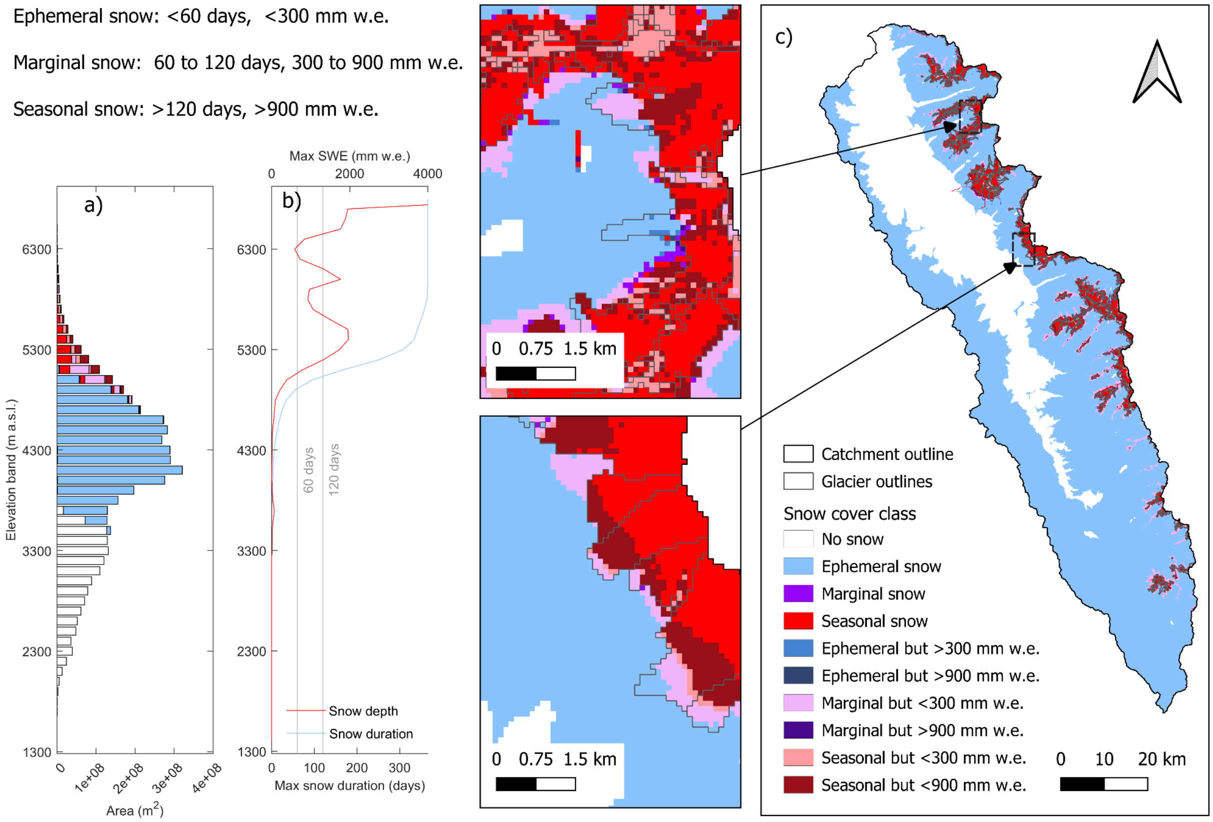

SI Figure 18 Classification of daily snow maps into ephemeral, marginal and seasonal snow. The classification was based on the average of the modelled maximum hydrological year snow water equivalent and continuous daily snow duration, applying the thresholds of Nadal-Romero and Lopez-Moreno (2024) and Lopez-Moreno et al. (2024). Note we apply the thresholds to values of SWE although the original values are for snow depths (converted from 0.5 m to 300 mm w.e. and 1.5 m to 900 mm w.e., based on the mean density of 24 snow pit measurements on Artesonraju Glacier of 0.6 g cm^{-3}). We additionally classify the snow cover if it is ephemeral, marginal or seasonal in duration but has a lower or higher SWE. a) shows the elevation distribution of the snow classification, b) the elevation distribution of the snow water equivalent

and snow duration and c) the map of the classification for the Rio Santa catchment with inset maps giving details at two selected locations.

R2C11: - Figure SI14: Part of the y-axis of the left plots is missing.

Thank you for spotting this mistake! SI Figure 16 (new numbering) has been fixed.

Hoping my comments will help,

Ignacio López-Moreno
